# Synthesis of pyrrolidinedione-fused hexahydropyrrolo[2,1-*a*]isoquinolines via three-component [3 + 2] cycloaddition followed by one-pot *N*-allylation and intramolecular Heck reactions

**DOI:** 10.3762/bjoc.16.106

**Published:** 2020-06-04

**Authors:** Xiaoming Ma, Suzhi Meng, Xiaofeng Zhang, Qiang Zhang, Shenghu Yan, Yue Zhang, Wei Zhang

**Affiliations:** 1 School of Pharmaceutical Engineering and Life Science, Changzhou University, Changzhou 213164, China; 2 Center for Green Chemistry and Department of Chemistry, University of Massachusetts Boston, 100 Morrissey Boulevard, Boston, MA 02125, USA; 3 Department of Cancer Biology, Dana-Farber Cancer Institute, Department of Medicine, Harvard Medical School, Boston, MA 02215, USA; 4 School of Chemistry, Biology and Materials Engineering, Suzhou University of Science and Technology, Suzhou 215009, China

**Keywords:** [3 + 2] cycloaddition, Heck reaction, hexahydropyrrolo[2,1-*a*]isoquinoline, one-pot reactions

## Abstract

Two kinds of [3 + 2] cycloaddition intermediates generated from the three-component reactions of 2-bromobenzaldehydes and maleimides with amino esters or amino acids were used for a one-pot *N*-allylation and intramolecular Heck reactions to form pyrrolidinedione-fused hexahydropyrrolo[2,1-*a*]isoquinolines. The multicomponent reaction was combined with one-pot reactions to make a synthetic method with good pot, atom and step economy. MeCN was used as a preferable green solvent for the reactions.

## Introduction

**Figure 1:**
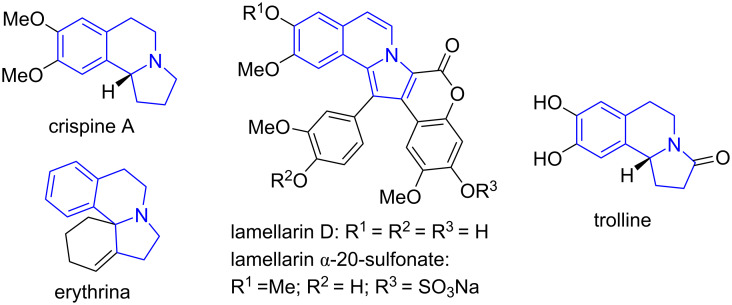
Bioactive pyrrolo[2,1-*a*]isoquinolines and hexahydropyrrolo[2,1-*a*]isoquinolines.

Pyrrolo[2,1-*a*]isoquinoline and hexahydropyrrolo[2,1-*a*]isoquinoline are privileged heterocyclic rings existing in many natural products and synthetic compounds possessing antitumor, antibacterial, antiviral, antioxidizing, and other biological activities (Figure 1) [1–2]. For example, the alkaloid crispine A isolated from *Carduus crispus L* has antitumor activity [3]. Erythrina alkaloids have curare-like neuromuscular blocking activities [4], and also antioxidant activity against DPPH free radicals [5]. Lamellarins isolated from marine invertebrates [6] are inhibitors for HIV-1 integrase and also have immuno modulatory activity [7–8]. Trolline has inhibitory activity against Gram-negative and Gram-positive bacteria [9], also as free radical scavenger in rat brain [10]. Organic chemists have been continuously interested in the development of methods for the synthesis of pyrrolo[2,1-*a*]isoquinolines and related ring systems [11–15], while medicinal chemists have also been interested in making related compounds for biological screening and drug development [16–17].

**Scheme 1:**
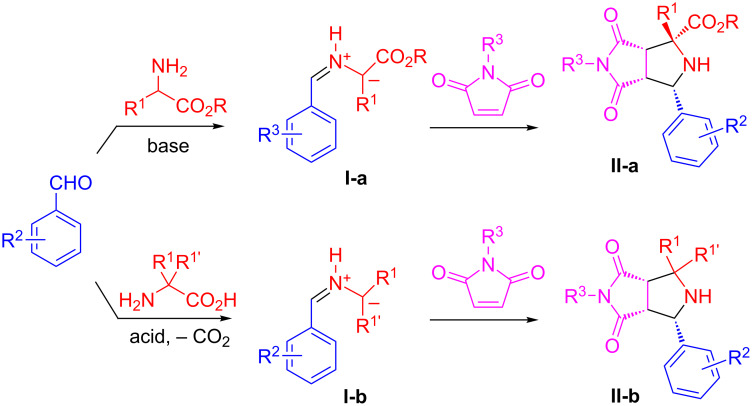
[3 + 2] Cycloaddition with amino esters or amino acids.

Multicomponent reactions (MCRs) have been developed as highly efficient tools for assembling heterocyclic scaffolds related to natural products [18–20]. Among the well-established MCRs, three-component 1,3-dipolar cycloadditions of benzaldehydes, maleimides, and amino esters have been developed for making *N*-containing 5-membered heterocycles (Scheme 1) [21–22]. The [3 + 2] cycloadditions of maleimides with stabilized azomethine ylides **I-a** generated from the condensation of aldehydes and amino esters for making pyrrolidines **II-a** have been well-reported [23–26], while the [3 + 2] cycloaddition of the less stable azomethine ylides **I-b** generated from the reaction of aldehydes and amino acids for pyrrolidines **II-b** was less explored [27–29].

**Scheme 2:**
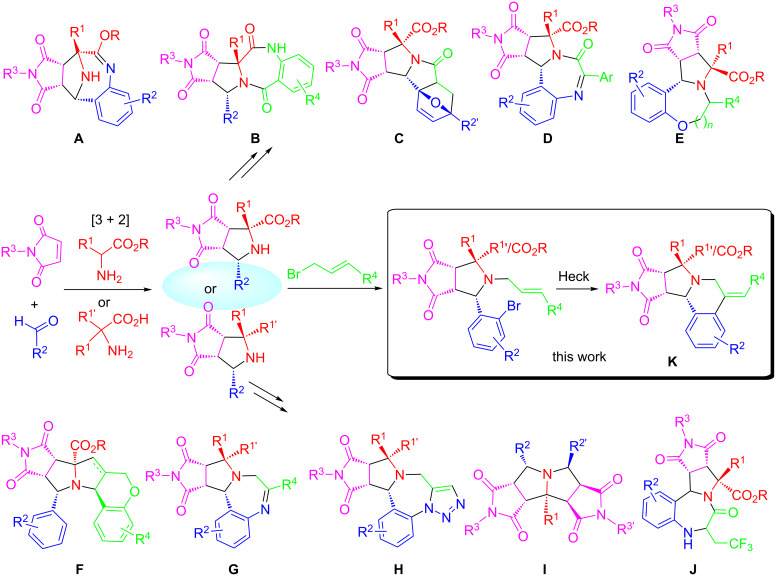
Scaffolds derived from the initial [3 + 2] adducts.

In recent years, our lab has reported a series of 1,3-dipolar cycloaddition-initiated methods for the synthesis of diverse heterocycles **A**–**J** bearing fused polycyclic rings such as tetrahydroepiminobenzo[*b*]azocines, tetrahydropyrrolobenzodiazepinones, triazolobenzodiazepines and tetrahydrochromeno[3,4-*b*]pyrrolizine (Scheme 2) [30–39]. Many of these scaffolds were synthesized through the combination of MCR and one-pot synthesis. A literature search indicated that a [3 + 2] cycloaddition-initiated method has also been used for the synthesis of hexahydropyrrolo[2,1-*a*]isoquinolines by employing stable 1,3-dilpolar compounds generated from amino esters [15,40] or isoquinolines [41–49]. We like to report in this paper our effort on the synthesis of pyrrolidinedione-fused hexahydropyrrolo[2,1-*a*]isoquinolines via sequential 1,3-dipolar cycloaddition, *N*-allylation, and intramolecular Heck cyclization reactions [50–54] (Scheme 2, **K**). Both stabilized and non-stabilized azomethine ylides could be used for the initial [3 + 2] cycloaddition. A multicomponent reaction was combined with one-pot reactions to make it a green synthetic method with pot, atom and step economy (PASE) [55–56].

## Results and Discussion

**Scheme 3:**
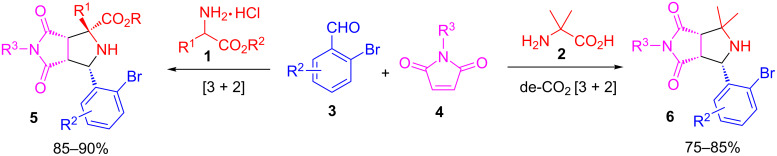
[3 + 2] Cycloaddition with amino esters or amino acids. Conditions: **1**:**3**:**4** (1.2:1:1.1), Et_3_N (1.5 equiv), EtOH (3 mL), 110 °C for 6 h; **2**:**3**:**4** (1.2:1:1), AcOH (0.3 equiv), MeCN (3 mL), 110 °C for 6 h.

Following the reported procedures for amino ester- and amino acid-based [3 + 2] cycloaddition reactions, pyrrolidine adducts **5** and **6** were synthesized by a three-component reaction of **1** or **2** with 2-bromobenzaldehydes **3** and maleimides **4** (Scheme 3) [30,37]. The cycloaddition reactions were diastereoselective (>20:1 dr for adducts **5** and >6:1 dr for adducts **6**). The major diastereomers of **5** and **6** were isolated for following *N*-allylation and intramolecular Heck reactions.

Adduct **5a** generated from [3 + 2] cycloaddition was used as a model compound to develop the reaction conditions for the one-pot *N*-allylation and intramolecular Heck reactions (Table 1). *N*-Allylation of **5a** with 3-bromopropene (**7**) for **8a** was accomplished by heating the reaction mixtures in MeCN at 105 °C for 4 h. After evaporating unreacted 3-bromopropene (**7**) from the reaction mixture, crude product **8a** was used for developing the intramolecular Heck reaction by screening Pd(II) catalysts, ligands, bases, additives, solvents, temperatures and reaction time (Table 1). The initial intramolecular Heck reactions were carried out using 10 mol % of Pd(OAc)_2_ or 10 mol % of PdCl_2_ with 20 mol % of PPh_3_ as a ligand and 2 equiv of K_2_CO_3_ in MeCN at 80 °C for 10 h without additive to give 6-*exo*-cyclized product **9a** in 32% and 18% yields, respectively (Table 1, entries 1 and 2). Addition of NaOAc increased the yield of **9a** to 71% (Table 1, entry 3). Other attempts to improve the Heck reaction using different ligands, such as (P(*o*-tol)_3_, PCy_3_ and dppm, were not successful (Table 1, entries 4–6). The reaction at 105 °C in MeCN gave **9a** in 78% yield (Table 1, entry 7), while at 120 °C in DMF gave **9a** in 77% yield (Table 1, entry 11). Reducing the amount of Pd(OAc)_2_ to 5 mol % or the reaction temperature to 40 °C gave lower product yields (Table 1, entries 8 and 10). Double the amount of Pd(OAc)_2_ to 20 mol% gave **9a** in 79% yield, just 1% increase than that of using 10 mol % of catalyst (Table 1, entry 9). Besides K_2_CO_3_, other bases including Na_2_CO_3_, Cs_2_CO_3_ and Et_3_N were also used for the Heck reaction, but none of them improved the product yield (Table 1, entries 12–14). A base-free control reaction gave **9a** in 10% yield (Table 1, entry 15). Thus, the optimized conditions for the Heck reaction was to use 10 mol % of Pd(OAc)_2_, 20 mol % of PPh_3_, 2 equiv of K_2_CO_3_ and 1 equiv of NaOAc in 3 mL of MeCN at 105 °C for 3 h which give **9a** in 78% yield (Table 1, entry 7). It is worth noting that there was no **9ab** observed as a byproduct because 6*-exo* cyclization is more favorable [50–51].

**Table 1:** Optimization of the one-pot reaction conditions.^a^

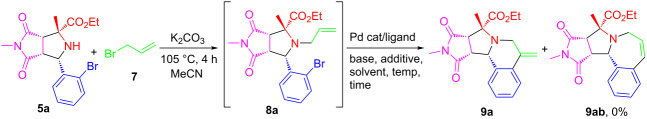								

entry	Pd Cat.	ligand	base	additive	solvent	temp [°C]	time [h]	yield [%]^b^

1	Pd(OAc)_2_	PPh_3_	K_2_CO_3_	–	MeCN	80	10	32
2	PdCl_2_	PPh_3_	K_2_CO_3_	–	MeCN	80	10	18
3	Pd(OAc)_2_	PPh_3_	K_2_CO_3_	NaOAc	MeCN	80	6	71
4	Pd(OAc)_2_	P(*o*-tol)_3_	K_2_CO_3_	NaOAc	MeCN	80	6	61
5	Pd(OAc)_2_	PCy_3_	K_2_CO_3_	NaOAc	MeCN	80	6	45
6	Pd(OAc)_2_	dppm	K_2_CO_3_	NaOAc	MeCN	80	6	58
**7**	**Pd(OAc)_2_**	**PPh_3_**	**K_2_CO_3_**	**NaOAc**	**MeCN**	**105**	**3**	**78**
8^c^	Pd(OAc)_2_	PPh_3_	K_2_CO_3_	NaOAc	MeCN	105	3	28
9^d^	Pd(OAc)_2_	PPh_3_	K_2_CO_3_	NaOAc	MeCN	105	3	79
10	Pd(OAc)_2_	PPh_3_	K_2_CO_3_	NaOAc	MeCN	40	12	13
11	Pd(OAc)_2_	PPh_3_	K_2_CO_3_	NaOAc	DMF	120	3	77
12	Pd(OAc)_2_	PPh_3_	Na_2_CO_3_	NaOAc	MeCN	105	6	19
13	Pd(OAc)_2_	PPh_3_	Cs_2_CO_3_	NaOAc	MeCN	105	6	34
14	Pd(OAc)_2_	PPh_3_	Et_3_N	NaOAc	MeCN	105	6	11
15	Pd(OAc)_2_	PPh_3_	–	NaOAc	MeCN	105	6	10

^a^Reaction conditions: 0.5 mmol **5a** in 3 mL MeCN, **7** (3 equiv), K_2_CO_3_ (2 equiv) for *N*-allylation; Pd catalyst (10 mol %), ligand (20 mol %), base (2 equiv) and NaOAc (1 equiv) in 3 mL solvent under nitrogen for the Heck reaction; P(*o*-tol)_3_ = tri(*o*-tolyl)phosphine, dppm = 1,1-bis(diphenylphosphino)methane. ^b^Isolated yield. ^c^Pd(OAc)_2_ 5 mol %, PPh_3_ 10 mol %. ^d^Pd(OAc)_2_ 20 mol %, PPh_3_ 40 mol %.

**Scheme 4:**
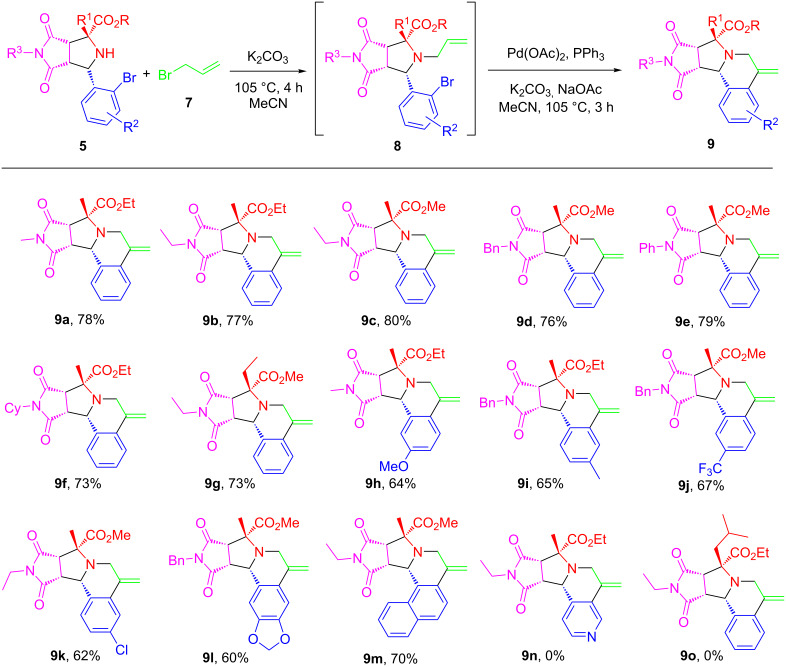
Synthesis of pyrrolo[2,1-*a*]isoquinolines **9**. Reaction conditions: **5** (0.5 mmol, 1 equiv), **7** (3 equiv) and K_2_CO_3_ (2 equiv) in MeCN (3 mL) for *N*-allylation; then Pd(OAc)_2_ (10 mol %), PPh_3_ (20 mol %), K_2_CO_3_ (2 equiv) and NaOAc (1 equiv) in MeCN (3 mL) under nitrogen for the Heck reaction. Isolated yield.

The optimized reaction conditions were then employed for the synthesis of analogs of **9** (Scheme 4). A variety of [3 + 2] cycloaddition adducts **5** bearing different R, R^1^, R^2^ and R^3^ groups, derived from amino esters **1**, 2-bromobenzaldehydes **3** and maleimides **4**, were subjected to *N*-allylation followed by intramolecular Heck reaction to pyrrolidinedione-fused hexahydropyrrolo[2,1-*a*]isoquinoline compounds **9a**–**o** in moderate to good yields as a single isomers which were confirmed by ^1^H NMR analysis of the crude reaction mixtures. The substitution groups R^3^ (Me, Et, Ph, Bn, *c*-C_6_H_11_) on maleimide have no significant influence on the product yields to afford **9a**–**f** in 73–80% yields. The substituent groups R^2^ including electron-donating (Me, OMe, -OCH_2_O) or -withdrawing groups (CF_3_, Cl) on the benzene ring have a little effect on the yield of products **9h**–**l**. Product **9m** bearing a naphthyl group was produced in 70% yield. Product **9n** containing a pyridine ring was not obtained due to the low yield at the *N*-allylation step. Same result happened to **9o** in which hindered iBu blocked the *N*-allylation.

**Scheme 5:**
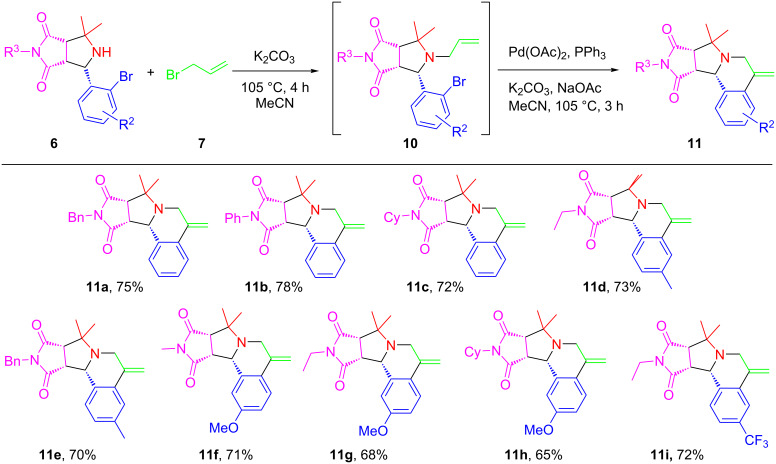
Synthesis of pyrrolo[2,1-*a*]isoquinolines **11**. Reaction conditions: **6** (0.5 mmol, 1 equiv), **7** (3 equiv) and K_2_CO_3_ (2 equiv) in MeCN (3 mL) for the *N*-allylation; then Pd(OAc)_2_ (10 mol %), PPh_3_ (20 mol %), K_2_CO_3_ (2 equiv) and NaOAc (1 equiv) in MeCN (3 mL) under nitrogen for the Heck reaction. Isolated yield.

We next employed intermediated **6** prepared from the decarboxylative [3+2] cycloaddition of amino acids for one-pot *N*-allylation and intramolecular Heck reactions under the same optimized conditions developed in Table 1. Pyrrolidinedione-fused hexahydropyrrolo[2,1-*a*]isoquinoline **11a**–**i** were produced in 65–78% yields also as single isomers (Scheme 5).

**Scheme 6:**
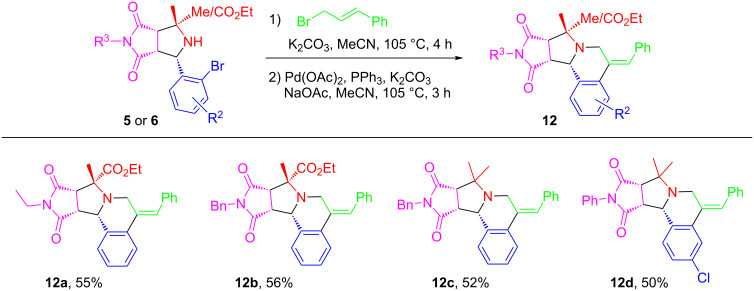
Synthesis of pyrrolo[2,1-*a*]isoquinolines **12**. Reaction conditions: **5** or **6** (0.5 mmol, 1 equiv), cinnamyl bromide (3 equiv) and K_2_CO_3_ (2 equiv) in MeCN (3 mL) for the *N*-allylation; then Pd(OAc)_2_ (10 mol %), PPh_3_ (20 mol %), K_2_CO_3_ (2 equiv) and NaOAc (1 equiv) in MeCN (3 mL) under nitrogen for the Heck reaction. Isolated yield.

Allylation of [3 + 2] adducts **5** or **6** with cinnamyl bromide were also conducted and the intermediates were used for the Heck reaction for making products **12a–d** (Scheme 6). Even the allylated intermediates were not terminal alkenes, the Heck reaction gave the *Z*-products exclusively [52].

**Scheme 7:**
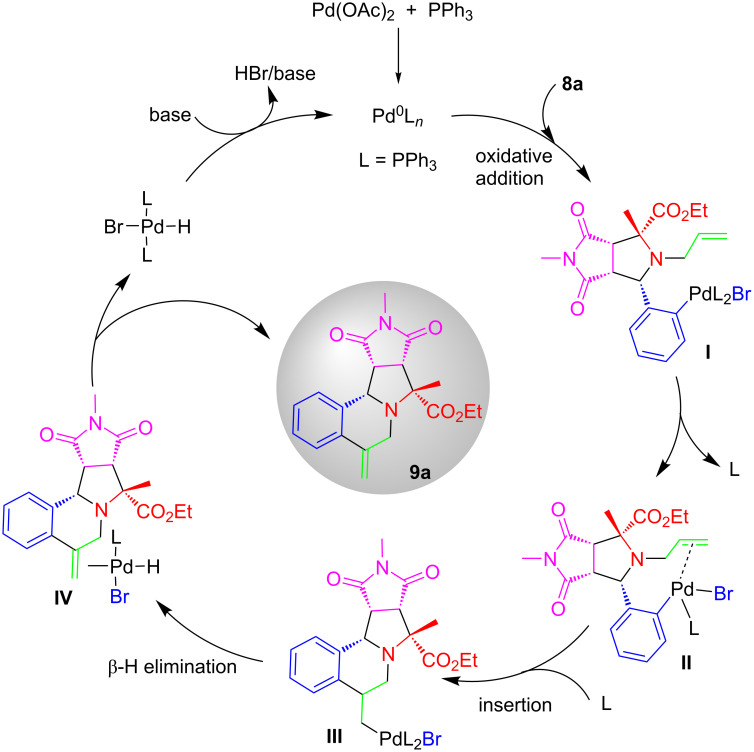
Plausible mechanism for the synthesis of **9a**.

A general mechanism for Pd-catalyzed intramolecular Heck reaction of **8a** for the synthesis of pyrrolo[2,1-*a*]isoquinoline **9a** is shown in Scheme 7. The oxidative addition of the Pd(0) species to alkene intermediate **8a** leads to Pd-complex **I**. Intramolecular coordination of Pd-complex **I** with the C–C double bond forms complex **II** which is followed by the *syn* insertion of alkene to give complex **III** [50–51]. Subsequent β-hydride elimination of **III** gives complex **IV** which undergoes dissociation to afford product **9a**. The hydridopalladium(II) halide is converted to the catalytically active Pd(0) with a base.

## Conclusion

In summary, we have developed an efficient method through a three-component [3 + 2] cycloaddition followed by a one-pot *N*-allylation and an intramolecular Heck reaction for the synthesis of pyrrolidinedione-fused hexahydropyrrolo[2,1-*a*]isoquinolines. Two different kinds of [3 + 2] adducts generated from the reactions of amino esters or amino acids were used as the key intermediates for sequential transformations. A high synthetic efficiency was achieved by the combination of a three-component reaction with one-pot reactions. This synthetic sequence is a new addition of our [3 + 2] cycloaddition-initiated reactions for making diverse cyclic scaffolds.

## Experimental

### General procedure for the synthesis of pyrrolidine adducts **5**

A solution of amino ester **1** (1.2 mmol), 2-bromobenzaldehyde **3** (1 mmol) and maleimide **4** (1.1 mmol) in EtOH (3 mL) with Et_3_N (1.5 mmol) was heated at 110 °C for 6 h in a sealed vial. The concentrated reaction mixture was isolated by column chromatography on silica gel to afford adduct **5** in 85–90% yield.

### General procedure for the synthesis of pyrrolidine adducts **6**

A solution of 2-aminoisobutyric acid (**2**, 1.2 mmol), 2-bromobenzaldehyde **3** (1 mmol) and maleimide **4** (1 mmol) in MeCN (3 mL) with AcOH (0.3 mmol) was heated at 110 °C for 6 h in a sealed vial. The concentrated reaction mixture was isolated by column chromatography on silica gel to afford adduct **6** in 75–85% yield.

### General procedure for the synthesis of pyrrolo[2,1-*a*]isoquinolines **9** or **11**

To a solution of pyrrolidine adduct **5** or **6** (0.5 mmol), 3-bromopropene (**7**, 1.5 mmol) in MeCN (3 mL) was added K_2_CO_3_ (1 mmol), the mixture was heated at 105 °C for 4 h in a sealed vial. Upon the completion of reaction as monitored by HPLC or LC–MS, the mixture was evaporated under vacuum to remove unreacted 3-bromopropene to give crude *N*-allylation intermediate **8** or **10**. Without further purification, it was used for the Heck reaction with Pd(OAc)_2_ (0.05 mmol), PPh_3_ (0.1 mmol), K_2_CO_3_ (1 mmol) and NaOAc (0.5 mmol) in MeCN (3 mL) at 105 °C for 3 h under nitrogen atmosphere. After aqueous work up, the crude product was purified by flash chromatography to afford product **9** or **11**.

### General procedure for the synthesis of pyrrolo[2,1-*a*]isoquinolines **12**

To a solution of pyrrolidine adduct **5** or **6** (0.5 mmol), cinnamyl bromide (1.5 mmol) in MeCN (3 mL) was added K_2_CO_3_ (1 mmol), the mixture was heated at 105 °C for 4 h in a sealed vial. Upon the completion of reaction as monitored by HPLC or LC–MS, the mixture was evaporated and the unreacted cinnamyl bromide was isolated to give *N*-allylation intermediate which was then used for the Heck reaction with Pd(OAc)_2_ (10 mol %), PPh_3_ (20 mol %), K_2_CO_3_ (2 equiv) and NaOAc (1 equiv) in MeCN (3 mL) at 105 °C for 3 h under nitrogen atmosphere. After aqueous work-up, the crude product was purified by flash chromatography to afford product **12**.

## Supporting Information

File 1 General reaction procedures, compound characterization data, and copies of NMR spectra.
