# Supplementary material for: Synthesis of pyrrolidinedione-fused hexahydropyrrolo[2,1-a]isoquinolines via three-component [3 + 2] cycloaddition followed by one-pot N-allylation and intramolecular Heck reactions
Source: Beilstein J Org Chem. 2020 Jun 4;16:1225–33. doi: 10.3762/bjoc.16.106 (PMC7277624; doi:10.3762/bjoc.16.106)
Supplement: File 1 — General reaction procedures, compound characterization data, and copies of NMR spectra. [file Beilstein_J_Org_Chem-16-1225-s001.pdf]

BEILSTEIN JOURNAL OF ORGANIC CHEMISTRY

# Supporting Information

for

# Synthesis of pyrrolidinedione-fused hexahydropyrrolo[2,1-*a*]isoquinolines via three-component [3 + 2] cycloaddition followed by one-pot *N*-allylation and intramolecular Heck reactions

Xiaoming Ma, Suzhi Meng, Xiaofeng Zhang, Qiang Zhang, Shenghu Yan, Yue Zhang and Wei Zhang

*Beilstein J. Org. Chem.* **2020,** *16,* 1225–1233. doi:10.3762/bjoc.16.106

# General reaction procedures, compound characterization data, and copies of NMR spectra

License and Terms: This is a supporting information file under the terms of the Creative Commons Attribution License (http://creativecommons.org/licenses/by/4.0). Please note that the reuse, redistribution and reproduction in particular requires that the authors and source are credited.
The license is subject to the *Beilstein Journal of Organic Chemistry* terms and conditions: (https://www.beilstein-journals.org/bjoc)

## Table of Contents


1. General information……………………………………………………………….….S1
2. General procedure for the synthesis of pyrrolidine adduct **5**…………………………………S2
3. General procedure for the synthesis of pyrrolidine adduct **6**…………………………………S2
4. General procedure for the synthesis of products **9** or **11**……………………………………..S2
5. General procedure for the synthesis of of products **12**……………………………………….S3
6. Characterization of selected pyrrolidine adduct **5** and **6**…………………………………...…S3
7. Characterization of products **9**, **11** and **12**……………………………………………………S5
8. $^{1}$H NMR and $^{13}$C NMR of selected pyrrolidine adduct **5** and **6** …………………………….S12
9. $^{1}$H NMR, $^{13}$C NMR and $^{19}$F NMR of products **9, 11** and **12**……………………………..…S20


### 1. General information

Chemicals and solvents were purchased from commercial sources and were used without further purification. $^{1}$H (300 or 400 MHz), $^{13}$C NMR spectra (75 MHz) and $^{19}$F NMR (282 MHz) were recorded on Bruker NMR spectrometers. LC–MS were performed on an Agilent 2100 system with C18 column (5.0 µm, 6.0 × 50 mm). The mobile phases were MeOH and $H_2O$ both containing 0.05% trifluoroacetic acid. A linear gradient was used to increase from 25:75 v/v MeOH/$H_2O$ to 100% MeOH in 7.0 min at a flow rate of 0.7 mL/min. UV detections were conducted at 210 nm, 254 nm and 365 nm. Low resolution mass spectra were recorded in APCI (atmospheric pressure chemical ionization). HRMS were performed on Agilent 6540 Q-TOF mass spectrometer (ESI). Flash column chromatography was performed using silica gel (200–300 mesh).

## 2. General procedures for the synthesis of pyrrolidine adducts 5

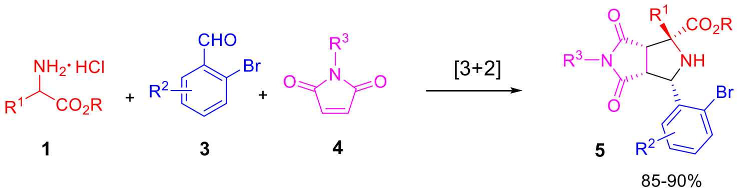


A solution of amino ester **1** (1.2 mmol), 2-bromobenzaldehyde **3** (1 mmol) and maleimide **4** (1.1 mmol) in EtOH (3 mL) with $Et_3N$ (1.5 mmol) was heated at 110 °C for 6 h in a sealed vial. The concentrated reaction mixture was isolated by column chromatography eluented with 15:85 ethyl acetate:petroleumn ether on silica gel to afford adduct **5** in 85–90% yield.

## 3. General procedures for the synthesis of pyrrolidine adducts 6

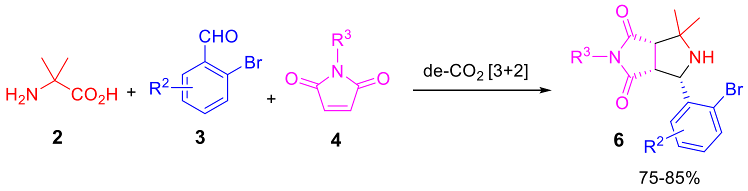


A solution of 2-aminoisobutyric acid **2** (1.2 mmol), 2-bromobenzaldehyde **3** (1 mmol) and maleimide **4** (1 mmol) in MeCN (3 mL) with AcOH (0.3 mmol) was heated at 110 °C for 6 h in a sealed vial. The concentrated reaction mixture was isolated by column chromatography eluented with 15:85 ethyl acetate:petroleumn ether on silica gel to afford adduct **6** in 75%-85% yield. Selected compound characterization data for adduct **6** are listed below.

## 4. General procedures for the synthesis of products 9 or 11

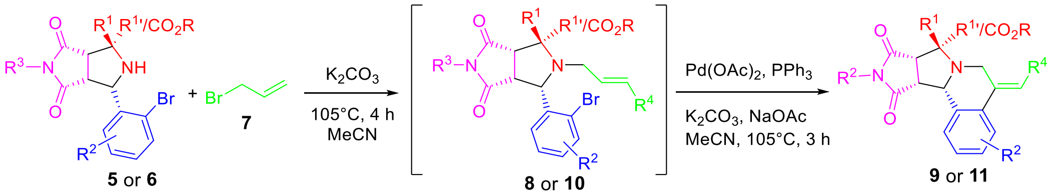


To a solution of pyrrolidine adduct **5** or **6** (0.5 mmol), 3-bromopropene (**7**, 1.5 mmol) in MeCN (3 mL) was added $K_2CO_3$ (1 mmol), the mixture was heated at 105 °C for 4 h in a sealed vial. Upon the completion of reaction as monitored by HPLC or LC–MS, the mixture was evaporated under vacuum to remove unreacted 3-bromopropene to give crude *N*-allylation intermediate **8**. Without further purification, it was used for the Heck reaction with $Pd(OAc)_2$ (0.05 mmol), $PPh_3$ (0.1 mmol), $K_2CO_3$ (1 mmol) and NaOAc (0.5 mmol) in MeCN (3 mL) at 105 °C for 3 h under nitrogen atmosphere. After aqueous work up, the crude product was purified by flash chromatography eluented with 20:80 ethyl acetate:petroleumn ether to afford product **9** or **11**.

Control experiments for the synthesis of **9a**

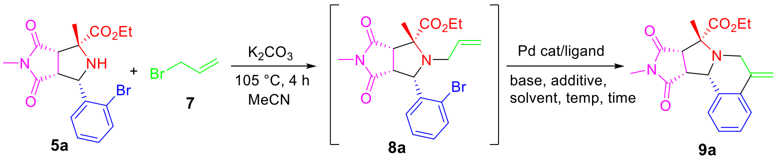

| Entry | Pd Cat. | Ligand | Base | Additive | Solvent | Temp/°C | Time/h | **9a**Yield/%[d] |
|---|---|---|---|---|---|---|---|---|
| 1 | $Pd(OAc)_2$ | - | $K_2CO_3$ | NaOAc | MeCN | 105 | 6 | Trace |
| 2 | $PdCl_2$ | - | $K_2CO_3$ | NaOAc | MeCN | 105 | 6 | Trace |
| 3 | - | $PPh_3$ | $K_2CO_3$ | NaOAc | MeCN | 105 | 6 | 0 |
| 4 | - | - | $K_2CO_3$ | NaOAc | MeCN | 105 | 6 | 0 |

[a] Reaction conditions: 0.5 mmol **5a** in 3 mL MeCN, **7** (3 equiv), $K_2CO_3$ (2 equiv) for *N*-allylation; Pd catalyst (10 mol %), ligand (20 mol %), base (2 euqiv) and NaOAc (1 equiv) in 3 mL solvent under nitrogen for the Heck reaction;

## 5. General procedures for the synthesis of products 12

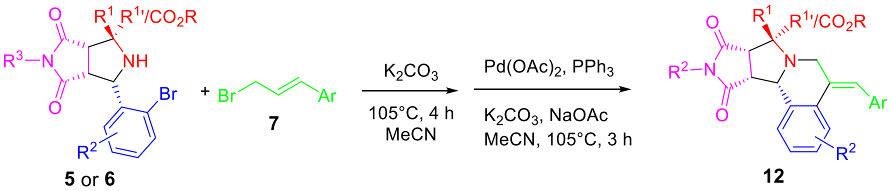

To a solution of pyrrolidine adduct **5** or **6** (0.5 mmol), cinnamyl bromide (1.5 mmol) in MeCN (3 mL) was added $K_2CO_3$ (1 mmol), the mixture was heated at 105 °C for 4 h in a sealed vial. Upon the completion of reaction as monitored by HPLC or LC–MS, the mixture was evaporated and the unreacted cinnamyl bromide was isolated to give *N*-allylation intermediate which was then used for the Heck reaction with $Pd(OAc)_2$ (10 mol %), $PPh_3$ (20 mol %), $K_2CO_3$ (2 equiv) and NaOAc (1 equiv) in MeCN (3 mL) at 105 °C for 3 h under nitrogen atmosphere. After aqueous work up, the crude product was purified by flash chromatography eluented with 20:80 ethyl acetate:petroleumn ether to afford product **12**.

## 6. Characterization of selected pyrrolidine adducts 5 and 6

*Ethyl (1R,3S,3aR,6aS)-3-(2-bromophenyl)-5-ethyl-1-methyl-4,6-dioxooctahydropyrrolo[3,4-c]pyrrole-1-carboxylate* (**5b**)

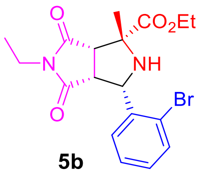

White solid, 368 mg, 90% yield. $^1$H NMR (300 MHz, $CDCl_3$) δ 7.60 (dd, *J* = 7.8, 1.3 Hz, 1H), 7.50 – 7.43 (m, 1H), 7.27 (s, 1H), 7.17 (td, *J* = 7.6, 1.7 Hz, 1H), 5.00 (dd, *J* = 8.4, 5.2 Hz, 1H), 4.34 (qd, *J* = 7.1, 1.0 Hz, 2H), 3.81 (dd, *J* = 8.8, 7.7 Hz, 1H), 3.31 (ddd, *J* = 22.2, 13.5, 7.2 Hz, 3H), 2.29 (d, *J* = 4.9 Hz, 1H), 1.65 (s, 3H), 1.39 (t, *J* = 7.2 Hz, 3H), 0.97 (t, *J* = 7.2 Hz, 3H). $^{13}$C NMR (75 MHz, $CDCl_3$) δ 175.5, 174.1, 172.3, 136.8, 132.5, 129.5, 127.40 (d, *J* = 2.3 Hz), 124.4, 66.8, 61.7, 60.4, 54.6, 46.9, 33.8, 24.0, 14.1, 13.1.

*Methyl (1R,3S,3aR,6aS)-3-(2-bromophenyl)-5-ethyl-1-methyl-4,6-dioxooctahydropyrrolo[3,4-c]pyrrole-1-carboxylate* (**5c**)

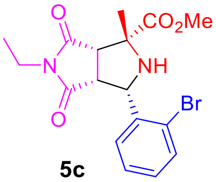

**5c**

White solid, 355 mg, 90% yield. $^{1}$H NMR (300 MHz, $CDCl_3$) δ 7.59 (dd, $J$ = 7.8, 1.3 Hz, 1H), 7.47 (dd, $J$ = 7.7, 1.6 Hz, 1H), 7.30 – 7.22 (m, 1H), 7.17 (td, $J$ = 7.6, 1.8 Hz, 1H), 5.00 (dd, $J$ = 8.8, 6.5 Hz, 1H), 3.88 (s, 3H), 3.81 (dd, $J$ = 8.8, 7.7 Hz, 1H), 3.31 (ddd, $J$ = 22.2, 11.0, 4.3 Hz, 3H), 2.26 (d, $J$ = 6.3 Hz, 1H), 1.65 (s, 3H), 0.97 (t, $J$ = 7.2 Hz, 3H). $^{13}$C NMR (75 MHz, $CDCl_3$) δ 175.6, 174.0, 172.8, 136.8, 132.5, 129.5, 127.4 (d, $J$ = 2.1 Hz), 124.4, 66.8, 60.42 (s), 54.6, 52.7, 46.8, 33.8, 23.9, 13.0.

*Ethyl (1R,3S,3aR,6aS)-3-(2-bromo-5-methoxyphenyl)-1,5-dimethyl-4,6-dioxooctahydropyrrolo[3,4-c]pyrrole-1-carboxylate* (**5h**)

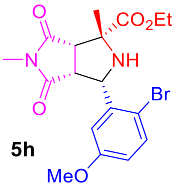

**5h**

White solid, 361 mg, 85% yield. $^{1}$H NMR (300 MHz, $CDCl_3$) δ 7.47 (d, $J$ = 8.7 Hz, 1H), 7.03 (d, $J$ = 3.0 Hz, 1H), 6.72 (dd, $J$ = 8.7, 3.1 Hz, 1H), 4.93 (d, $J$ = 8.0 Hz, 1H), 4.33 (q, $J$ = 7.1 Hz, 2H), 3.91 – 3.78 (m, 1H), 3.74 (s, 3H), 3.27 (d, $J$ = 7.6 Hz, 1H), 2.78 (s, 3H), 2.24 (s, 1H), 1.64 (s, 3H), 1.38 (t, $J$ = 7.2 Hz, 3H). $^{13}$C NMR (75 MHz, $CDCl_3$) δ 175.7, 174.2, 172.3, 159.2, 138.1, 133.1, 114.5 (d, $J$ = 7.1 Hz), 113.6, 66.7, 61.7, 60.3, 55.5, 54.5, 47.2, 24.8, 24.0, 14.1.

*Ethyl (1R,3S,3aR,6aS)-5-benzyl-3-(2-bromo-4-methylphenyl)-1-methyl-4,6-dioxooctahydro-pyrrolo[3,4-c]pyrrole-1-carboxylate* (**5i**)

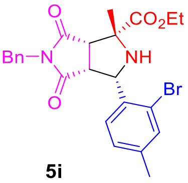

**5i**

White solid, 422 mg, 87% yield. $^{1}$H NMR (300 MHz, $CDCl_3$) δ 7.39 (d, $J$ = 0.8 Hz, 1H), 7.25 (d, $J$ = 1.1 Hz, 5H), 6.99 (d, $J$ = 7.9 Hz, 1H), 6.80 (d, $J$ = 7.9 Hz, 1H), 5.00 – 4.90 (m, 1H), 4.41 (d, $J$ = 4.9 Hz, 2H), 4.33 (q, $J$ = 6.9 Hz, 2H), 3.81 – 3.74 (m, 1H), 3.27 (d, $J$ = 7.6 Hz, 1H), 2.27 (d, $J$ = 7.2 Hz, 4H), 1.63 (s, 3H), 1.37 (t, $J$ = 7.2 Hz, 3H). $^{13}$C NMR (75 MHz, $CDCl_3$) δ 175.3, 174.0, 172.2, 139.3, 135.6, 133.1 (d, $J$ = 18.1 Hz), 129.0, 128.4 (d, $J$ = 12.9 Hz), 127.8, 126.9, 123.9, 66.9, 61.8, 60.5, 54.9, 47.2, 42.4, 23.9, 20.8, 14.1.

*Methyl (1R,3S,3aR,6aS)-3-(2-bromonaphthalen-1-yl)-5-ethyl-1-methyl-4,6-dioxooctahydro-pyrrolo[3,4-c]pyrrole-1-carboxylate* (**5m**)

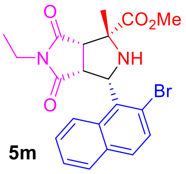

**5m**

White solid, 382 mg, 86% yield. $^{1}$H NMR (400 MHz, $CDCl_3$) δ 8.35 (d, $J$ = 8.5 Hz, 1H), 7.80 (d, $J$ = 8.1 Hz, 1H), 7.75 (d, $J$ = 8.6 Hz, 1H), 7.64 – 7.56 (m, 2H), 7.51 (t, $J$ = 7.4 Hz, 1H), 5.33 (d, $J$ = 4.4 Hz, 1H), 3.94 – 3.83 (m, 4H), 3.35 (dd, $J$ = 13.7, 6.9 Hz, 1H), 3.27 (dd, $J$ = 12.8, 7.2 Hz, 2H), 2.30 (s, 1H), 1.69 (s, 3H), 0.99 (t, $J$ = 7.2 Hz, 3H). $^{13}$C NMR (101 MHz, $CDCl_3$) δ 175.6, 174.0, 172.9, 134.7, 134.4, 132.2, 128.3, 127.8, 127.5 (d, $J$ = 15.9 Hz), 126.7, 124.6, 124.4, 66.9, 61.3, 54.4, 52.6, 46.8, 33.9, 24.0, 13.0.

*(3aS,6S,6aR)-2-Benzyl-6-(2-bromophenyl)-4,4-dimethyltetrahydropyrrolo[3,4-c]pyrrole-1,3(2H,3aH)-dione* (**6a**)

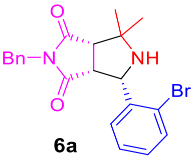

**6a**

White solid, 343 mg, 83% yield. $^{1}$H NMR (300 MHz, $CDCl_3$) δ 7.59 – 7.52 (m, 1H), 7.27 (s, 7H), 7.11 (pd, $J$ = 7.3, 4.0 Hz, 2H), 4.95 (d, $J$ = 8.1 Hz, 1H), 4.49 (d, $J$ = 2.6 Hz, 2H), 3.76 (t, $J$ = 7.9 Hz, 1H), 2.90 (d, $J$ = 7.8 Hz, 1H), 1.42 (s, 3H), 1.40 (s, 3H). $^{13}$C NMR (75 MHz, $CDCl_3$) δ 176.3, 174.9, 137.8, 136.0, 132.3, 129.0, 128.7, 128.4, 128.1, 127.7, 127.2, 124.1, 60.3, 59.8, 53.6, 47.6, 42.1, 29.0, 26.5.

*(3aS,6S,6aR)-6-(2-Bromophenyl)-2-cyclohexyl-4,4-dimethyltetrahydropyrrolo[3,4-c]pyrrole-1,3(2H,3aH)-dione* (**6c**)

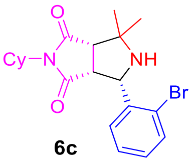

**6c**

White solid, 332 mg, 82% yield. $^{1}$H NMR (300 MHz, $CDCl_3$) δ 7.58 (dd, *J* = 7.8, 1.3 Hz, 1H), 7.48 (dd, *J* = 7.7, 1.5 Hz, 1H), 7.24 (dd, *J* = 7.6, 1.1 Hz, 1H), 7.15 (td, *J* = 7.6, 1.8 Hz, 1H), 4.94 (d, *J* = 8.0 Hz, 1H), 3.79 (ddd, *J* = 12.3, 8.4, 3.9 Hz, 1H), 3.68 (t, *J* = 7.9 Hz, 1H), 2.82 (d, *J* = 7.8 Hz, 1H), 2.04 (dd, *J* = 12.5, 3.5 Hz, 1H), 1.90 (dd, *J* = 12.5, 3.5 Hz, 1H), 1.73 (d, *J* = 12.9 Hz, 2H), 1.57 (d, *J* = 10.6 Hz, 2H), 1.45 (s, 3H), 1.40 (s, 3H), 1.32 – 1.05 (m, 3H). $^{13}$C NMR (75 MHz, $CDCl_3$) δ 176.7, 175.3, 138.2, 132.3, 129.0, 128.1, 127.1, 124.2, 60.3, 59.8, 53.1, 51.6, 47.1, 29.1, 28.7 (d, *J* = 3.0 Hz), 26.5, 25.8 (d, *J* = 3.7 Hz), 24.9.

*(3aS,6S,6aR)-6-(2-Bromo-4-(trifluoromethyl)phenyl)-2-ethyl-4,4-dimethyltetrahydropyrrolo[3,4-c]pyrrole-1,3(2H,3aH)-dione* (**6i**)

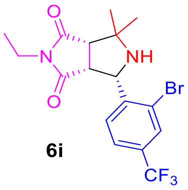

**6i**

White solid, 314 mg, 75% yield. $^{1}$H NMR (300 MHz, $CDCl_3$) δ 7.76 (d, *J* = 2.1 Hz, 1H), 7.70 (d, *J* = 8.3 Hz, 1H), 7.41 (dd, *J* = 8.3, 2.1 Hz, 1H), 4.98 (d, *J* = 8.3 Hz, 1H), 3.75 (t, *J* = 8.0 Hz, 1H), 3.43 – 3.29 (m, 2H), 2.88 (d, *J* = 7.8 Hz, 1H), 1.50 (s, 3H), 1.42 (s, 3H), 1.00 (t, *J* = 7.2 Hz, 3H). $^{13}$C NMR (75 MHz, $CDCl_3$) δ 176.1, 174.9, 139.8, 132.8, 129.9, 129.5, 128.0, 125.7 (d, *J* = 3.9 Hz), 125.1 (d, *J* = 3.8 Hz), 122.1, 59.9 (d, *J* = 6.5 Hz), 53.2, 47.2, 33.5, 29.0, 26.4, 13.1.

## 7. Characterization of products 9, 11 and 12

*Ethyl (8R,8aS,11aR,11bS)-8,10-dimethyl-5-methylene-9,11-dioxo-5,8,8a,9,10,11,11a,11b-octahydro-6H-pyrrolo-[3',4':3,4]pyrrolo[2,1-a]isoquinoline-8-carboxylate* (**9a**)

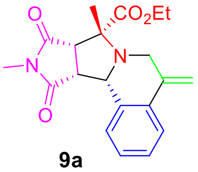

**9a**

Orange solid, 138 mg, 78% yield. $^{1}$H NMR (300 MHz, $CDCl_3$) δ 7.66 (dd, *J* = 7.8, 1.3 Hz, 1H), 7.56 (d, *J* = 7.6 Hz, 1H), 7.31 (td, *J* = 7.5, 1.5 Hz, 1H), 7.25 (dt, *J* = 11.7, 3.9 Hz, 1H), 5.56 (d, *J* = 1.4 Hz, 1H), 4.97 (d, *J* = 1.2 Hz, 1H), 4.39 (d, *J* = 7.1 Hz, 1H), 4.30 – 4.22 (m, 1H), 4.21 – 4.11 (m, 1H), 3.70 (t, *J* = 7.4 Hz, 1H), 3.47 (d, *J* = 12.0 Hz, 1H), 3.24 – 3.16 (m, 2H), 2.82 (s, 3H), 1.46 (s, 3H), 1.29 (d, *J* = 7.2 Hz, 3H). $^{13}$C NMR (75 MHz, $CDCl_3$) δ 175.5, 174.7, 171.3, 139.0, 132.3 (d, *J* = 4.9 Hz), 128.4, 127.0 (d, *J* = 13.5 Hz), 124.0, 108.4, 69.8, 62.0, 61.6, 54.4, 50.6, 45.6, 25.1, 14.6, 14.1. HRMS (ESI) calcd for $C_{20}H_{23}N_2O_4$ ([M+H]$^+$): 355.1652, found 355.1655.

*Ethyl (8R,8aS,11aR,11bS)-10-ethyl-8-methyl-5-methylene-9,11-dioxo-5,8,8a,9,10,11,11a,11b-octahydro-6H-pyrrolo[3',4':3,4]pyrrolo[2,1-a]isoquinoline-8-carboxylate* (**9b**)

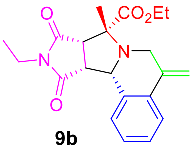

**9b**

Orange solid, 141 mg, 77% yield. $^{1}$H NMR (400 MHz, $CDCl_3$) δ 7.66 (d, *J* = 7.8 Hz, 1H), 7.55 (d, *J* = 7.6 Hz, 1H), 7.31 (t, *J* = 7.4 Hz, 1H), 7.27 – 7.22 (m, 1H), 5.55 (s, 1H), 4.96 (s, 1H), 4.39 (d, *J* = 7.0 Hz, 1H), 4.33 – 4.26 (m, 1H), 4.17 – 4.08 (m, 1H), 3.68 (t, *J* = 7.4 Hz, 1H), 3.47 (d, *J* = 11.9 Hz, 1H), 3.38 (q, *J* = 7.1 Hz, 2H), 3.22 – 3.14 (m, 2H), 1.46 (s, 3H), 1.29 (t, *J* = 7.1 Hz, 3H), 1.01 (t, *J* = 7.2 Hz, 3H). $^{13}$C NMR (101 MHz, $CDCl_3$) δ 175.3, 174.5, 171.2, 139.1, 132.4 (d, *J* = 8.3 Hz), 128.4, 127.0, 126.8, 124.0, 108.2, 69.9, 61.9, 61.6, 54.3, 50.6, 45.6, 34.0, 14.7, 14.1, 12.7. HRMS (ESI) calcd for $C_{21}H_{25}N_2O_4$ ([M+H]$^+$): 369.1809, found 369.1807.

*Methyl (8R,8aS,11aR,11bS)-10-ethyl-8-methyl-5-methylene-9,11-dioxo-5,8,8a,9,10,11,11a,11b-octahydro-6H-pyrrolo[3',4':3,4]pyrrolo[2,1-a]isoquinoline-8-carboxylate* (**9c**)

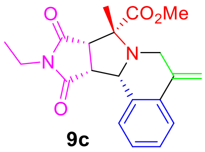

**9c**

Orange solid, 141 mg, 80% yield. $^{1}$H NMR (400 MHz, $CDCl_3$) δ 7.66 (d, *J* = 7.4 Hz, 1H), 7.55 (d, *J* = 7.7 Hz, 1H), 7.31 (t, *J* = 6.9 Hz, 1H), 7.24 (d, *J* = 7.4 Hz, 1H), 5.57 (s, 1H), 4.98 (s, 1H), 4.39 (d, *J* = 7.0 Hz, 1H), 3.76 (s, 3H), 3.68 (t, *J* = 7.4 Hz, 1H), 3.48 (d, *J* = 12.0 Hz, 1H), 3.42 – 3.33 (m, 2H), 3.20 (d, *J* = 12.0 Hz, 1H), 3.16 (d, *J* = 7.7 Hz, 1H), 1.47 (s, 3H),

1.01 (t, $J$ = 7.2 Hz, 3H). $^{13}$C NMR (75 MHz, $CDCl_3$) δ 175.4, 174.4, 171.7, 138.9, 132.2 (d, $J$ = 8.8 Hz), 128.4, 126.9 (d, $J$ = 18.0 Hz), 124.0, 108.4, 70.0, 62.0, 54.3, 52.6, 50.6, 45.5, 34.1, 14.6, 12.6. HRMS (ESI) calcd for $C_{20}H_{23}N_2O_4$ ([M+H]$^+$): 355.1652, found 355.1655.

*Methyl (8R,8aS,11aR,11bS)-10-benzyl-8-methyl-5-methylene-9,11-dioxo-5,8,8a,9,10,11,11a,11b-octahydro-6H-pyrrolo[3',4':3,4]pyrrolo[2,1-a]isoquinoline-8-carboxylate* (**9d**)

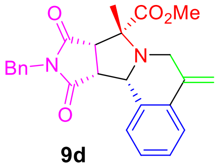


**9d**

Orange solid, 158 mg, 76% yield. $^1$H NMR (300 MHz, $CDCl_3$) δ 7.65 (dd, $J$ = 7.8, 1.2 Hz, 1H), 7.53 (d, $J$ = 7.6 Hz, 1H), 7.32 – 7.23 (m, 2H), 7.18 (d, $J$ = 4.4 Hz, 5H), 5.60 (s, 1H), 5.02 (s, 1H), 4.49 (d, $J$ = 4.8 Hz, 2H), 4.41 (d, $J$ = 7.3 Hz, 1H), 3.68 (t, $J$ = 7.4 Hz, 1H), 3.58 (s, 3H), 3.52 (d, $J$ = 11.8 Hz, 1H), 3.19 (dd, $J$ = 9.6, 6.5 Hz, 2H), 1.46 (s, 3H). $^{13}$C NMR (75 MHz, $CDCl_3$) δ 175.2, 174.3, 171.7, 139.2, 135.2, 132.3 (d, $J$ = 15.1 Hz), 128.4 (d, $J$ = 8.7 Hz), 128.1, 127.5, 127.0 (d, $J$ = 15.6 Hz), 124., 108.4, 70.1, 62.1, 54.3, 52.4, 50.6, 45.5, 42.4, 14.5. HRMS (ESI) calcd for $C_{25}H_{25}N_2O_4$ ([M+H]$^+$): 417.1809, found 417.1804.

*Methyl (8R,8aS,11aR,11bS)-8-methyl-5-methylene-9,11-dioxo-10-phenyl-5,8,8a,9,10,11,11a,11b-octahydro-6H-pyrrolo[3',4':3,4]pyrrolo[2,1-a]isoquinoline-8-carboxylate* (**9e**)

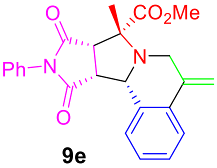


**9e**

Orange solid, 159 mg, 79% yield. $^1$H NMR (400 MHz, $CDCl_3$) δ 7.65 (dd, $J$ = 7.9, 0.9 Hz, 1H), 7.54 (d, $J$ = 7.7 Hz, 1H), 7.37 – 7.31 (m, 2H), 7.28 (t, $J$ = 4.9 Hz, 2H), 7.23 (d, $J$ = 7.4 Hz, 1H), 7.13 (dd, $J$ = 5.5, 3.4 Hz, 2H), 5.57 (d, $J$ = 1.1 Hz, 1H), 4.99 (d, $J$ = 0.8 Hz, 1H), 4.45 (d, $J$ = 6.7 Hz, 1H), 3.84 (dd, $J$ = 7.6, 6.9 Hz, 1H), 3.76 (s, 3H), 3.50 (d, $J$ = 12.0 Hz, 1H), 3.30 (d, $J$ = 7.8 Hz, 1H), 3.25 (d, $J$ = 11.9 Hz, 1H), 1.52 (s, 3H). $^{13}$C NMR (75 MHz, $CDCl_3$) δ 174.9, 173.8, 173.2, 171.8, 139.0, 132.2 (d, $J$ = 4.9 Hz), 131.8, 128.9, 128.4 (d, $J$ = 11.6 Hz), 126.9 (d, $J$ = 8.3 Hz), 126.6, 124.0, 110.9, 108.4, 70.7, 62.1, 54.4, 53.5, 52.7, 50.5, 45.9, 14.9. HRMS (ESI) calcd for $C_{24}H_{23}N_2O_4$ ([M+H]$^+$): 403.1652, found 403.1656.

*Ethyl (8R,8aS,11aR,11bS)-10-cyclohexyl-8-methyl-5-methylene-9,11-dioxo-5,8,8a,9,10,11,11a,11b-octahydro-6H-pyrrolo[3',4':3,4]pyrrolo[2,1-a]isoquinoline-8-carboxylate* (**9f**)

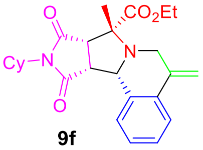


**9f**

Orange solid, 154 mg, 73% yield. $^1$H NMR (300 MHz, $CDCl_3$) δ 7.64 (dd, $J$ = 7.8, 1.3 Hz, 1H), 7.54 – 7.49 (m, 1H), 7.30 (td, $J$ = 7.5, 1.5 Hz, 1H), 7.26 – 7.20 (m, 1H), 5.52 (d, $J$ = 1.0 Hz, 1H), 4.93 (s, 1H), 4.37 – 4.26 (m, 2H), 4.15 – 4.03 (m, 1H), 3.83 – 3.68 (m, 1H), 3.60 (dd, $J$ = 7.6, 7.0 Hz, 1H), 3.47 – 3.41 (m, 1H), 3.17 (d, $J$ = 11.8 Hz, 1H), 3.07 (d, $J$ = 7.7 Hz, 1H), 2.01 – 1.87 (m, 2H), 1.70 (d, $J$ = 10.4 Hz, 2H), 1.53 (dd, $J$ = 7.1, 3.7 Hz, 2H), 1.44 (s, 3H), 1.27 (d, $J$ = 7.2 Hz, 3H), 1.22 – 1.04 (m, 4H). $^{13}$C NMR (75 MHz, $CDCl_3$) δ 175.5, 174.8, 171.2, 139.4, 132.5 (d, $J$ = 13.8 Hz), 128.4, 126.8 (d, $J$ = 16.4 Hz), 124.0, 108.0, 70.0, 61.9, 61.4, 53.9, 51.9, 50.4, 45.2, 28.6, 28.3, 25.8 (d, $J$ = 3.5 Hz), 24.9, 14.8, 14.1. HRMS (ESI) calcd for $C_{25}H_{31}N_2O_4$ ([M+H]$^+$): 423.2278, found 423.2275.

*Methyl (8R,8aS,11aR,11bS)-8,10-diethyl-5-methylene-9,11-dioxo-5,8,8a,9,10,11,11a,11b-octahydro-6H-pyrrolo-[3',4':3,4]pyrrolo[2,1-a]isoquinoline-8-carboxylate* (**9g**)

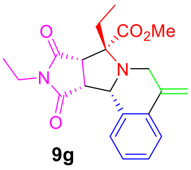


**9g**

Orange solid, 134 mg, 73% yield. $^1$H NMR (300 MHz, $CDCl_3$) δ 7.62 (dd, $J$ = 7.8, 1.3 Hz, 1H), 7.51 (d, $J$ = 7.6 Hz, 1H), 7.32 – 7.21 (m, 2H), 5.50 (s, 1H), 4.96 (d, $J$ = 0.9 Hz, 1H), 4.56 (d, $J$ = 7.3 Hz, 1H), 3.74 (d, $J$ = 11.5 Hz, 4H), 3.64 (t, $J$ = 7.5 Hz, 1H), 3.44 – 3.31 (m, 3H), 3.26 (d, $J$ = 7.7 Hz, 1H), 2.11 (dt, $J$ = 14.3, 7.2 Hz, 1H), 1.93 – 1.79 (m, 1H), 1.14 (t, $J$ = 7.4 Hz, 3H), 0.97 (t, $J$ = 7.2 Hz, 3H). $^{13}$C NMR (75 MHz, $CDCl_3$) δ 175.6, 174.5, 171.2, 139.3, 132.50 (d, $J$ = 16.5 Hz), 128.5, 126.85 (d, $J$ = 17.8 Hz), 124.1, 108.0, 73.4, 61.9, 52.32 (d, $J$ = 16.7 Hz), 51.4, 46.0, 34.0, 23.7, 12.7, 10.6. HRMS (ESI) calcd for $C_{21}H_{25}N_2O_4$ ([M+H]$^+$): 369.1809, found 369.1813.

*Ethyl (8R,8aS,11aR,11bS)-2-methoxy-8,10-dimethyl-5-methylene-9,11-dioxo-5,8,8a,9,10,11,-11a,11b-octahydro-6H-pyrrolo[3',4':3,4]pyrrolo[2,1-a]isoquinoline-8-carboxylate* (**9h**)

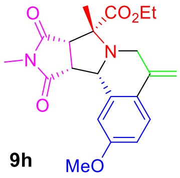


**9h**

Orange solid, 123 mg, 64% yield. $^{1}$H NMR (400 MHz, $CDCl_3$) δ 7.59 (d, $J$ = 8.8 Hz, 1H), 7.09 (d, $J$ = 2.0 Hz, 1H), 6.82 (dd, $J$ = 8.8, 2.3 Hz, 1H), 5.42 (s, 1H), 4.86 (s, 1H), 4.36 (d, $J$ = 7.2 Hz, 1H), 4.30 – 4.23 (m, 1H), 4.17 (dd, $J$ = 10.7, 7.1 Hz, 1H), 3.87 (s, 3H), 3.69 (s, 1H), 3.46 (d, $J$ = 12.0 Hz, 1H), 3.20 (s, 1H), 3.17 (d, $J$ = 5.2 Hz, 1H), 2.83 (s, 3H), 1.46 (s, 3H), 1.29 (t, $J$ = 7.1 Hz, 3H). $^{13}$C NMR (75 MHz, $CDCl_3$) δ 175.4, 174.7, 171.3, 158.3, 138.6, 133.7, 125.25 (d, $J$ = 11.7 Hz), 113.8, 112.9, 106.4, 69.8, 62.3, 61.6, 55.4, 54.4, 50.8, 45.6, 25.2, 14.6, 14.1. HRMS (ESI) calcd for $C_{21}H_{25}N_2O_5$ ($[M+H]^+$): 385.1758, found 385.1761.

*Ethyl (8R,8aS,11aR,11bS)-10-benzyl-3,8-dimethyl-5-methylene-9,11-dioxo-5,8,8a,9,10,11,11a,11b-octahydro-6H-pyrrolo[3',4':3,4]pyrrolo[2,1-a]isoquinoline-8-carboxylate* (**9i**)

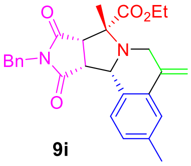


**9i**

Orange solid, 144 mg, 65% yield. $^{1}$H NMR (400 MHz, $CDCl_3$) δ 7.45 (s, 1H), 7.42 (d, $J$ = 7.9 Hz, 1H), 7.18 (s, 5H), 7.12 (d, $J$ = 7.9 Hz, 1H), 5.57 (s, 1H), 4.98 (s, 1H), 4.50 (s, 2H), 4.38 (d, $J$ = 7.2 Hz, 1H), 4.25 – 4.17 (m, 1H), 3.96 (dq, $J$ = 10.7, 7.1 Hz, 1H), 3.65 (t, $J$ = 7.4 Hz, 1H), 3.50 (d, $J$ = 11.7 Hz, 1H), 3.19 (s, 1H), 3.16 (d, $J$ = 3.9 Hz, 1H), 2.33 (s, 3H), 1.45 (s, 3H), 1.16 (t, $J$ = 7.1 Hz, 3H). $^{13}$C NMR (75 MHz, $CDCl_3$) δ 175.1, 174.5, 171.2, 139.5, 136.5, 135.2, 132.3, 129.4, 128.35 (d, $J$ = 5.2 Hz), 128.00 (d, $J$ = 3.3 Hz), 127.5, 124.5, 107.9, 69.9, 62.0, 61.5, 54.4, 50.6, 45.6, 42.3, 29.7, 21.4, 14.5, 14.0. HRMS (ESI) calcd for $C_{27}H_{29}N_2O_4$ ($[M+H]^+$): 445.2122, found 445.2127.

*Methyl (8R,8aS,11aR,11bS)-10-benzyl-8-methyl-5-methylene-9,11-dioxo-2-(trifluoromethyl)-5,8,8a,9,10,11,11a,-11b-octahydro-6H-pyrrolo[3',4':3,4]pyrrolo[2,1-a]isoquinoline-8-carboxylate* (**9j**)

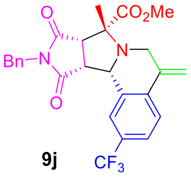


**9j**

Orange solid, 162 mg, 67% yield. $^{1}$H NMR (300 MHz, $CDCl_3$) δ 7.81 (s, 1H), 7.74 (d, $J$ = 8.3 Hz, 1H), 7.47 (d, $J$ = 8.3 Hz, 1H), 7.18 (s, 5H), 5.69 (s, 1H), 5.15 (s, 1H), 4.50 (d, $J$ = 2.3 Hz, 2H), 4.43 (d, $J$ = 7.5 Hz, 1H), 3.73 (dd, $J$ = 8.7, 6.2 Hz, 1H), 3.62 – 3.51 (m, 4H), 3.23 (d, $J$ = 2.1 Hz, 1H), 3.20 (d, $J$ = 6.6 Hz, 1H), 1.47 (s, 3H). $^{19}$F NMR (282 MHz, $CDCl_3$) δ -62.48 (s). $^{13}$C NMR (75 MHz, $CDCl_3$) δ 174.9, 173.9, 171.4, 138.2, 135.8, 135.0, 132.7, 128.69 (d, $J$ = 21.5 Hz), 128.4, 127.9, 127.5, 125.94 (d, $J$ = 3.8 Hz), 124.6, 123.68 (d, $J$ = 3.7 Hz), 110.7, 70.0, 61.9, 54.2, 52.5, 50.2, 45.2, 42.4, 29.7, 14.5. HRMS (ESI) calcd for $C_{26}H_{24}F_3N_2O_4$ ($[M+H]^+$): 485.1683, found 485.1687.

*Methyl(8R,8aS,11aR,11bS)-3-chloro-10-ethyl-8-methyl-5-methylene-9,11-dioxo-5,8,8a,9,10,11,-11a,11b-octahydro-6H-pyrrolo[3',4':3,4]pyrrolo[2,1-a]isoquinoline-8-carboxylate* (**9k**)

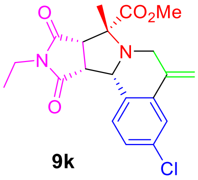


**9k**

Orange solid, 120 mg, 62% yield. $^{1}$H NMR (400 MHz, $CDCl_3$) δ 7.62 (s, 1H), 7.50 (d, $J$ = 8.4 Hz, 1H), 7.28 (s, 1H), 5.56 (s, 1H), 5.03 (s, 1H), 4.33 (d, $J$ = 7.0 Hz, 1H), 3.77 (s, 3H), 3.66 (t, $J$ = 7.4 Hz, 1H), 3.49 (d, $J$ = 12.0 Hz, 1H), 3.39 (dd, $J$ = 14.1, 6.9 Hz, 2H), 3.17 (d, $J$ = 7.9 Hz, 2H), 1.47 (s, 3H), 1.01 (t, $J$ = 7.2 Hz, 3H). $^{13}$C NMR (101 MHz, $CDCl_3$) δ 175.2, 174.3, 171.5, 137.9, 134.1, 133.0, 130.7, 129.9, 126.9, 124.0, 109.7, 70.0, 61.6, 54.2, 52.6, 50.3, 45.3, 34.1, 14.7, 12.7. HRMS (ESI) calcd for $C_{20}H_{22}ClN_2O_4$ ($[M+H]^+$): 389.1263, found 389.1260.

*Methyl (8R,8aS,11aR,11bS)-10-benzyl-8-methyl-5-methylene-9,11-dioxo-5,8,8a,9,10,11,11a,11b-octahydro-6H-[1,3]dioxolo[4,5-g]pyrrolo[3',4':3,4]pyrrolo[2,1-a]isoquinoline-8-carboxylate* (**9l**)

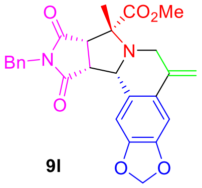


**9l**

Orange solid, 138 mg, 60% yield. $^1$H NMR (300 MHz, DMSO) δ 7.24 – 7.20 (m, 2H), 7.19 (d, *J* = 1.8 Hz, 2H), 7.13 (dd, *J* = 7.0, 2.6 Hz, 2H), 7.07 (s, 1H), 6.00 (d, *J* = 6.5 Hz, 2H), 5.49 (s, 1H), 4.88 (s, 1H), 4.47 (d, *J* = 15.5 Hz, 1H), 4.37 (d, *J* = 15.5 Hz, 1H), 4.29 (d, *J* = 7.1 Hz, 1H), 3.97 (t, *J* = 7.3 Hz, 1H), 3.47 (s, 3H), 3.38 (d, *J* = 3.4 Hz, 2H), 3.07 (d, *J* = 11.9 Hz, 1H), 1.37 (s, 3H). $^{13}$C NMR (75 MHz, DMSO) δ 176.0, 175.5, 171.9, 146.8, 146.6, 139.8, 135.9, 128.6, 128.1, 127.4 (d, *J* = 11.0 Hz), 126.5, 109.0, 107.2, 103.6, 101.4, 70.0, 61.8, 54.5, 52.4, 50.4, 45.9, 41.6, 14.9. HRMS (ESI) calcd for $C_{26}H_{25}N_2O_6$ ([M+H]$^+$): 461.1707, found 461.1703.

*Methyl(10R,10aS,13aR,13bS)-12-ethyl-10-methyl-7-methylene-11,13-dioxo-7,10,10a,11,12,-13,13a,13b-octahydro-8H-benzo[h]pyrrolo[3',4':3,4]pyrrolo[2,1-a]isoquinoline-10-carboxylate* (**9m**)

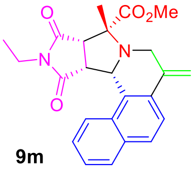


**9m**

Orange solid, 141 mg, 70% yield. $^1$H NMR (300 MHz, $CDCl_3$) δ 8.56 – 8.47 (m, 1H), 7.87 – 7.80 (m, 1H), 7.77 (d, *J* = 8.5 Hz, 1H), 7.59 (d, *J* = 8.5 Hz, 1H), 7.49 – 7.40 (m, 2H), 5.64 (s, 1H), 5.51 (s, 1H), 4.51 (d, *J* = 6.6 Hz, 1H), 3.78 (s, 3H), 3.75 – 3.67 (m, 1H), 3.50 (d, *J* = 10.3 Hz, 1H), 3.38 (q, *J* = 7.2 Hz, 2H), 3.29 (d, *J* = 10.3 Hz, 1H), 3.15 (d, *J* = 7.5 Hz, 1H), 1.47 (s, 3H), 0.99 (t, *J* = 7.2 Hz, 3H). $^{13}$C NMR (75 MHz, $CDCl_3$) δ 175.6, 174.8, 171.8, 138.2, 133.4, 130.71 – 130.21 (m), 128.6, 126.9, 126.1, 125.8 – 125.2 (m), 116.9, 70.2, 62.7, 54.2, 52.5 (d, *J* = 2.7 Hz), 45.7, 34.0, 14.7, 12.7. HRMS (ESI) calcd for $C_{24}H_{25}N_2O_4$ ([M+H]$^+$): 405.1809, found 405.1814.

*(8aS,11aR,11bS)-10-Benzyl-8,8-dimethyl-5-methylene-5,6,8,8a,11a,11b-hexahydro-9H-pyrrolo[3',4':3,4]pyrrolo[2,1-a]isoquinoline-9,11(10H)-dione* (**11a**)

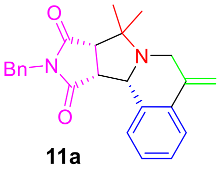


**11a**

Orange solid, 139 mg, 75% yield. $^1$H NMR (300 MHz, $CDCl_3$) δ 7.66 (dd, *J* = 7.8, 1.1 Hz, 1H), 7.51 (d, *J* = 7.6 Hz, 1H), 7.29 (dd, *J* = 7.6, 1.4 Hz, 1H), 7.25 (dd, *J* = 5.5, 2.5 Hz, 1H), 7.22 – 7.17 (m, 5H), 5.57 (s, 1H), 5.00 (s, 1H), 4.55 (d, *J* = 14.5 Hz, 1H), 4.46 (d, *J* = 14.5 Hz, 1H), 4.36 (d, *J* = 6.5 Hz, 1H), 3.67 – 3.58 (m, 1H), 3.49 (d, *J* = 12.0 Hz, 1H), 3.14 (d, *J* = 11.9 Hz, 1H), 2.93 (d, *J* = 7.6 Hz, 1H), 1.26 (s, 3H), 1.14 (s, 3H). $^{13}$C NMR (75 MHz, $CDCl_3$) δ 176.2, 175.3, 140.2, 135.6, 133.2, 132.4, 128.4, 128.1, 127.5, 126.9 (d, *J* = 1.3 Hz), 124.0, 107.8, 62.4, 61.9 53.9, 49.1, 46.0, 42.1, 24.1, 19.6. HRMS (ESI) calcd for $C_{24}H_{25}N_2O_2$ ([M+H]$^+$): 373.1911, found 373.1914.

*(8aS,11aR,11bS)-8,8-Dimethyl-5-methylene-10-phenyl-5,6,8,8a,11a,11b-hexahydro-9H-pyrrolo[3',4':3,4]pyrrolo[2,1-a]isoquinoline-9,11(10H)-dione* (**11b**)

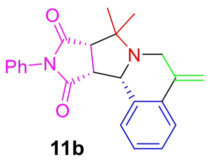


**11b**

Orange solid, 140 mg, 78% yield. $^1$H NMR (300 MHz, $CDCl_3$) δ 7.64 (dd, *J* = 7.8, 1.3 Hz, 1H), 7.54 – 7.49 (m, 1H), 7.37 – 7.21 (m, 5H), 7.18 – 7.13 (m, 2H), 5.55 (d, *J* = 1.2 Hz, 1H), 4.99 (d, *J* = 1.1 Hz, 1H), 4.39 (d, *J* = 6.1 Hz, 1H), 3.78 (dd, *J* = 7.6, 6.3 Hz, 1H), 3.54 (d, *J* = 12.2 Hz, 1H), 3.18 (d, *J* = 12.0 Hz, 1H), 3.07 (d, *J* = 7.7 Hz, 1H), 1.42 (s, 3H), 1.21 (s, 3H). $^{13}$C NMR (75 MHz, $CDCl_3$) δ 175.8, 174.6, 140.0, 133.2, 132.3, 131.8, 128.9, 128.3 (d, *J* = 3.5 Hz), 126.9 (d, *J* = 5.8 Hz), 126.5, 123.9, 107.8, 62.6, 62.4, 54.0, 49.4, 46.2, 45.2, 24.5, 20.1. HRMS (ESI) calcd for $C_{23}H_{23}N_2O_2$ ([M+H]$^+$): 359.1754, found 359.1757.

*(8aS,11aR,11bS)-10-Cyclohexyl-8,8-dimethyl-5-methylene-5,6,8,8a,11a,11b-hexahydro-9H-pyrrolo[3',4':3,4]pyrrolo[2,1-a]isoquinoline-9,11(10H)-dione* (**11c**)

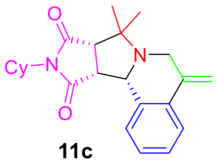

**11c**

Orange solid, 131 mg, 72% yield. $^{1}$H NMR (300 MHz, $CDCl_3$) δ 7.65 (d, $J$ = 7.5 Hz, 1H), 7.48 (d, $J$ = 7.5 Hz, 1H), 7.27 (ddd, $J$ = 18.4, 10.9, 6.6 Hz, 2H), 5.53 (s, 1H), 4.95 (s, 1H), 4.28 (d, $J$ = 6.0 Hz, 1H), 3.79 (tt, $J$ = 12.3, 3.7 Hz, 1H), 3.55 (dd, $J$ = 7.4, 6.3 Hz, 1H), 3.46 (d, $J$ = 12.1 Hz, 1H), 3.13 (d, $J$ = 12.1 Hz, 1H), 2.83 (d, $J$ = 7.6 Hz, 1H), 2.14 – 1.89 (m, 2H), 1.71 (d, $J$ = 11.0 Hz, 2H), 1.55 (d, $J$ = 6.9 Hz, 1H), 1.44 (d, $J$ = 12.1 Hz, 2H), 1.29 (s, 3H), 1.21 – 1.06 (m, 6H). $^{13}$C NMR (75 MHz, $CDCl_3$) δ 176.9, 175.8, 140.2, 133.6, 132.2, 128.2, 126.8, 123.9, 107.6, 62.4, 61.9, 53.6, 51.6, 49.3, 45.7, 29.0, 28.3, 25.8 (d, $J$ = 3.5 Hz), 25.0, 24.2, 20.2. HRMS (ESI) calcd for $C_{23}H_{29}N_2O_2$ ($[M+H]^+$): 365.2224, found 365.2222.

*(8aS,11aR,11bS)-10-Ethyl-3,8,8-trimethyl-5-methylene-5,6,8,8a,11a,11b-hexahydro-9H-pyrrolo[3',4':3,4]pyrrolo[2,1-a]isoquinoline-9,11(10H)-dione* (**11d**)

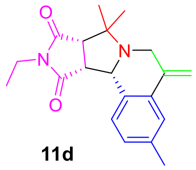

**11d**

Orange solid, 118 mg, 73% yield. $^{1}$H NMR (400 MHz, $CDCl_3$) δ 7.46 (s, 1H), 7.41 (d, $J$ = 7.8 Hz, 1H), 7.13 (d, $J$ = 7.8 Hz, 1H), 5.53 (s, 1H), 4.94 (s, 1H), 4.26 (d, $J$ = 6.0 Hz, 1H), 3.59 (t, $J$ = 6.8 Hz, 1H), 3.46 (d, $J$ = 12.1 Hz, 1H), 3.42 – 3.30 (m, 2H), 3.11 (d, $J$ = 12.0 Hz, 1H), 2.90 (d, $J$ = 7.5 Hz, 1H), 2.35 (s, 3H), 1.31 (s, 3H), 1.14 (s, 3H), 1.02 (t, $J$ = 7.0 Hz, 3H). $^{13}$C NMR (101 MHz, $CDCl_3$) δ 176.5, 175.6, 140.2, 136.2, 132.0, 130.6, 128.2, 127.9, 124.3, 107.5, 62.2, 61.8, 53.9, 49.4, 46.0, 33.6, 24.3, 21.4, 19.9, 12.9. HRMS (ESI) calcd for $C_{20}H_{25}N_2O_2$ ($[M+H]^+$): 325.1911, found 325.1914.

*(8aS,11aR,11bS)-10-Benzyl-3,8,8-trimethyl-5-methylene-5,6,8,8a,11a,11b-hexahydro-9H-pyrrolo[3',4':3,4]pyrrolo[2,1-a]isoquinoline-9,11(10H)-dione* (**11e**)

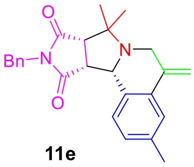

**11e**

Orange solid, 135 mg, 70% yield. $^{1}$H NMR (400 MHz, $CDCl_3$) δ 7.46 (s, 1H), 7.40 (d, $J$ = 7.9 Hz, 1H), 7.20 (s, 5H), 7.13 (d, $J$ = 7.8 Hz, 1H), 5.55 (s, 1H), 4.98 (s, 1H), 4.55 (d, $J$ = 14.5 Hz, 1H), 4.46 (d, $J$ = 14.5 Hz, 1H), 4.31 (d, $J$ = 6.4 Hz, 1H), 3.60 (t, $J$ = 7.0 Hz, 1H), 3.47 (d, $J$ = 12.0 Hz, 1H), 3.11 (d, $J$ = 11.9 Hz, 1H), 2.91 (d, $J$ = 7.5 Hz, 1H), 2.34 (s, 3H), 1.26 (s, 3H), 1.13 (s, 3H). $^{13}$C NMR (101 MHz, $CDCl_3$) δ 176.3, 175.4, 140.4, 136.3, 135.7, 132.2, 130.4, 128.2 (dd, $J$ = 25.0, 11.9 Hz), 127.5, 124.4, 107.5, 62.3, 61.9, 53.9, 49.2, 46.1, 42.1, 24.2, 21.4, 19.6. HRMS (ESI) calcd for $C_{25}H_{27}N_2O_2$ ($[M+H]^+$): 387.2067, found 387.2071.

*(8aS,11aR,11bS)-2-Methoxy-8,8,10-trimethyl-5-methylene-5,6,8,8a,11a,11b-hexahydro-9H-pyrrolo[3',4':3,4]pyrrolo[2,1-a]isoquinoline-9,11(10H)-dione* (**11f**)

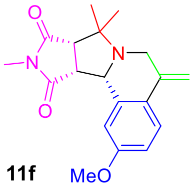

**11f**

Orange solid, 115 mg, 71% yield. $^{1}$H NMR (300 MHz, $CDCl_3$) δ 7.59 (d, $J$ = 8.8 Hz, 1H), 7.05 (d, $J$ = 2.2 Hz, 1H), 6.80 (dd, $J$ = 8.8, 2.3 Hz, 1H), 5.42 (d, $J$ = 1.2 Hz, 1H), 4.86 (d, $J$ = 1.1 Hz, 1H), 4.27 (d, $J$ = 6.6 Hz, 1H), 3.86 (s, 3H), 3.62 (t, $J$ = 7.2 Hz, 1H), 3.47 (d, $J$ = 12.2 Hz, 1H), 3.11 (d, $J$ = 12.2 Hz, 1H), 2.94 (d, $J$ = 7.7 Hz, 1H), 2.82 (s, 3H), 1.33 (s, 3H), 1.14 (s, 3H). $^{13}$C NMR (75 MHz, $CDCl_3$) δ 176.6, 175.6, 158.4, 139.5, 134.7, 125.1 (d, $J$ = 10.0 Hz), 113.6, 112.9, 105.8, 62.7, 61.8, 55.4, 54.0, 49.5, 45.8, 24.8, 24.3, 19.6. HRMS (ESI) calcd for $C_{19}H_{23}N_2O_3$ ($[M+H]^+$): 327.1703, found 327.1701.

*(8aS,11aR,11bS)-10-Ethyl-2-methoxy-8,8-dimethyl-5-methylene-5,6,8,8a,11a,11b-hexahydro-9H-pyrrolo[3',4':3,4]pyrrolo[2,1-a]isoquinoline-9,11(10H)-dione* (**11g**)

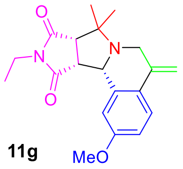


**11g**

Orange solid, 115 mg, 68% yield. $^1$H NMR (400 MHz, $CDCl_3$) δ 7.59 (d, $J$ = 8.8 Hz, 1H), 7.04 (d, $J$ = 2.3 Hz, 1H), 6.81 (dd, $J$ = 8.8, 2.5 Hz, 1H), 5.40 (s, 1H), 4.85 (s, 1H), 4.28 (d, $J$ = 6.5 Hz, 1H), 3.86 (s, 3H), 3.60 (t, $J$ = 7.1 Hz, 1H), 3.46 (d, $J$ = 12.2 Hz, 1H), 3.39 (q, $J$ = 7.1 Hz, 2H), 3.11 (d, $J$ = 12.1 Hz, 1H), 2.91 (d, $J$ = 7.7 Hz, 1H), 1.32 (s, 3H), 1.15 (s, 3H), 1.03 (t, $J$ = 7.2 Hz, 3H). $^{13}$C NMR (101 MHz, $CDCl_3$) δ 176.4, 175.3, 158.4, 139.6, 134.7, 125.2, 113.4, 112.9, 105.6, 62.6, 61.8, 55.4, 53.9, 49.5, 45.9, 33.7, 24.3, 19.8, 12.9. HRMS (ESI) calcd for $C_{20}H_{25}N_2O_3$ ([M+H]$^+$): 341.1860, found 341.1864.

*(8aS,11aR,11bS)-10-Cyclohexyl-2-methoxy-8,8-dimethyl-5-methylene-5,6,8,8a,11a,11b-hexahydro-9H-pyrrolo[3',4':3,4]pyrrolo[2,1-a]isoquinoline-9,11(10H)-dione* (**11h**)

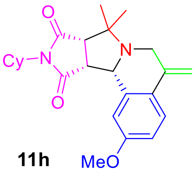


**11h**

Orange solid, 128 mg, 65% yield. $^1$H NMR (400 MHz, $CDCl_3$) δ 7.58 (d, $J$ = 8.8 Hz, 1H), 6.99 (s, 1H), 6.81 (d, $J$ = 8.6 Hz, 1H), 5.38 (s, 1H), 4.84 (s, 1H), 4.25 (d, $J$ = 6.0 Hz, 1H), 3.85 (s, 3H), 3.79 (t, $J$ = 12.3 Hz, 1H), 3.53 (t, $J$ = 6.8 Hz, 1H), 3.43 (d, $J$ = 12.1 Hz, 1H), 3.11 (d, $J$ = 12.1 Hz, 1H), 2.82 (d, $J$ = 7.6 Hz, 1H), 2.03 (dq, $J$ = 36.3, 12.1 Hz, 2H), 1.72 (d, $J$ = 10.7 Hz, 2H), 1.55 (d, $J$ = 11.2 Hz, 1H), 1.46 (d, $J$ = 11.8 Hz, 2H), 1.29 (s, 3H), 1.20 (s, 1H), 1.13 (s, 3H). $^{13}$C NMR (75 MHz, $CDCl_3$) δ 176.8, 175.6, 158.3, 139.8, 134.9, 125.2, 113.4, 112.7, 105.5, 62.6, 61.9, 55.3, 53.5, 51.6, 49.4, 45.6, 29.0, 28.4, 25.8 (d, $J$ = 3.2 Hz), 25.0, 24.2, 20.1. HRMS (ESI) calcd for $C_{24}H_{31}N_2O_3$ ([M+H]$^+$): 395.2329, found 395.2327.

*(8aS,11aR,11bS)-10-Ethyl-8,8-dimethyl-5-methylene-3-(trifluoromethyl)-5,6,8,8a,11a,11b-hexahydro-9H-pyrrolo[3',4':3,4]pyrrolo[2,1-a]isoquinoline-9,11(10H)-dione* (**11i**)

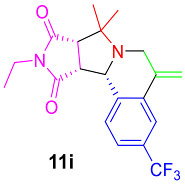


**11i**

Orange solid, 136 mg, 72% yield. $^1$H NMR (400 MHz, $CDCl_3$) δ 7.81 – 7.72 (m, 2H), 7.47 (d, $J$ = 8.3 Hz, 1H), 5.64 (s, 1H), 5.10 (s, 1H), 4.32 (d, $J$ = 6.3 Hz, 1H), 3.65 (t, $J$ = 7.0 Hz, 1H), 3.52 (d, $J$ = 12.3 Hz, 1H), 3.39 (q, $J$ = 7.1 Hz, 2H), 3.15 (d, $J$ = 12.2 Hz, 1H), 2.94 (d, $J$ = 7.6 Hz, 1H), 1.33 (s, 3H), 1.17 (s, 3H), 1.03 (t, $J$ = 7.2 Hz, 3H). $^{19}$F NMR (282 MHz, $CDCl_3$) δ -62.52 (s). $^{13}$C NMR (101 MHz, $CDCl_3$) δ 176.2, 175.1, 139.0, 133.9, 124.4, 123.4, 110.1, 62.2, 61.9, 53.8, 53.4, 49.0, 45.6, 33.7, 29.7, 24.2, 19.8, 12.9. HRMS (ESI) calcd for $C_{20}H_{22}F_3N_2O_2$ ([M+H]$^+$): 379.1628, found 379.1631.

*Ethyl (8R,8aS,11aR,11bS)-5-((Z)-benzylidene)-10-ethyl-8-methyl-9,11-dioxo-5,8,8a,9,10,11,11a,-11b-octahydro-6H-pyrrolo[3',4':3,4]pyrrolo[2,1-a]isoquinoline-8-carboxylate* (**12a**)

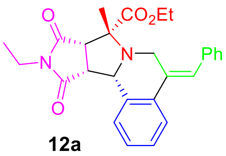


**12a**

Orange solid, 122 mg, 55% yield. $^1$H NMR (300 MHz, $CDCl_3$) δ 7.76 – 7.68 (m, 1H), 7.60 – 7.53 (m, 1H), 7.32 (dd, $J$ = 9.7, 4.3 Hz, 4H), 7.22 (dd, $J$ = 10.2, 4.4 Hz, 3H), 7.14 (s, 1H), 4.44 (d, $J$ = 6.9 Hz, 1H), 4.00 – 3.89 (m, 2H), 3.85 – 3.75 (m, 1H), 3.68 (t, $J$ = 7.3 Hz, 1H), 3.36 (q, $J$ = 7.2 Hz, 2H), 3.13 (t, $J$ = 11.2 Hz, 2H), 1.46 (s, 3H), 1.00 (t, $J$ = 7.2 Hz, 3H), 0.78 (t, $J$ = 7.2 Hz, 3H). $^{13}$C NMR (75 MHz, $CDCl_3$) δ 175.2, 174.7, 171.3, 137.2, 133.6, 133.2, 132.6, 129.1, 128.4 (d, $J$ = 18.8 Hz), 127.0 (d, $J$ = 19.1 Hz), 126.6, 124.2, 123.9, 70.1, 61.7, 61.4, 54.2, 45.7, 45.3, 34.0, 14.8, 13.4, 12.7. HRMS (ESI) calcd for $C_{27}H_{29}N_2O_4$ ([M+H]$^+$): 445.2122, found 445.2126.

*Ethyl (8R,8aS,11aR,11bS)-10-benzyl-5-((Z)-benzylidene)-8-methyl-9,11-dioxo-5,8,8a,9,10,11,11a,-11b-octahydro-6H-pyrrolo[3',4':3,4]pyrrolo[2,1-a]isoquinoline-8-carboxylate* (**12b**)

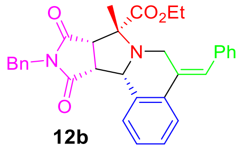


**12b**

Orange solid, 142 mg, 56% yield. $^1$H NMR (300 MHz, $CDCl_3$) δ 7.72 (dd, $J$ = 7.7, 1.5 Hz, 1H), 7.55 (dd, $J$ = 7.1, 1.3 Hz, 1H), 7.37 – 7.22 (m, 7H), 7.19 (d, $J$ = 7.2 Hz, 6H), 4.48 (d, $J$ = 3.7 Hz, 3H), 4.03 (d, $J$ = 11.9 Hz, 1H), 3.84 (dq, $J$ = 10.7, 7.1 Hz, 1H), 3.67 (t, $J$ = 7.4 Hz, 1H), 3.56 (dq, $J$ = 10.7, 7.2 Hz, 1H), 3.15 (dd, $J$ = 10.0, 5.5 Hz, 2H), 1.46 (s, 3H), 0.60 (t, $J$ = 7.2 Hz, 3H). $^{13}$C NMR (75 MHz, $CDCl_3$) δ 174.9, 174.5, 171.2, 137.2, 135.2, 133.8, 133.4, 132.4, 129.1, 128.4 (t, $J$ = 10.8 Hz), 127.9, 127.4 (d, $J$ = 12.9 Hz), 127.0, 126.7, 124.4, 123.8, 70.2, 61.9, 61.4, 54.2, 45.6, 45.2, 42.2, 14.5, 13.2. HRMS (ESI) calcd for $C_{32}H_{31}N_2O_4$ ([M+H]$^+$): 507.2278, found 507.2278.

*(8aS,11aR,11bS)-10-Benzyl-5-((Z)-benzylidene)-8,8-dimethyl-5,6,8,8a,11a,11b-hexahydro-9H-pyrrolo[3',4':3,4]pyrrolo[2,1-a]isoquinoline-9,11(10H)-dione* (**12c**)

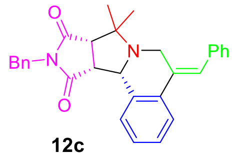


**12c**

Orange solid, 116 mg, 52% yield. $^1$H NMR (300 MHz, $CDCl_3$) δ 7.76 – 7.71 (m, 1H), 7.54 (dd, $J$ = 7.1, 1.2 Hz, 1H), 7.41 – 7.31 (m, 3H), 7.27 (ddd, $J$ = 10.6, 6.1, 2.4 Hz, 5H), 7.18 (t, $J$ = 2.2 Hz, 5H), 4.55 (d, $J$ = 14.5 Hz, 1H), 4.46 (d, $J$ = 12.9 Hz, 1H), 4.42 (d, $J$ = 4.8 Hz, 1H), 4.03 (d, $J$ = 12.3 Hz, 1H), 3.69 – 3.59 (m, 1H), 3.09 (d, $J$ = 12.6 Hz, 1H), 2.92 (d, $J$ = 7.6 Hz, 1H), 1.15 (s, 3H), 1.12 (s, 3H). $^{13}$C NMR (75 MHz, $CDCl_3$) δ 176.2, 175.4, 137.4, 135.6, 134.1, 133.7, 133.4, 129.2, 128.7 – 128.2 (m), 128.1, 127.5, 127.2 – 126.5 (m), 124.1, 123.2, 62.2 (d, $J$ = 10.8 Hz), 53.9, 46.1, 43.8, 42.1, 24.1, 19.8. HRMS (ESI) calcd for $C_{30}H_{29}N_2O_2$ ([M+H]$^+$): 449.2224, found 449.2227.

*(8aS,11aR,11bS)-5-((Z)-Benzylidene)-3-chloro-8,8-dimethyl-10-phenyl-5,6,8,8a,11a,11b-hexahydro-9H-pyrrolo[3',4':3,4]pyrrolo[2,1-a]isoquinoline-9,11(10H)-dione* (**12d**)

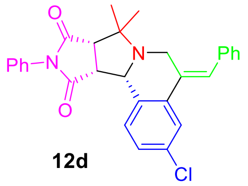


**12d**

Orange solid, 117 mg, 50% yield. $^1$H NMR (300 MHz, $CDCl_3$) δ 7.68 (d, $J$ = 2.0 Hz, 1H), 7.46 (d, $J$ = 8.4 Hz, 1H), 7.36 (dd, $J$ = 7.2, 4.3 Hz, 3H), 7.31 – 7.21 (m, 6H), 7.13 (dd, $J$ = 5.3, 3.1 Hz, 3H), 4.36 (d, $J$ = 6.1 Hz, 1H), 4.05 (d, $J$ = 12.5 Hz, 1H), 3.73 (dd, $J$ = 7.7, 6.2 Hz, 1H), 3.10 (d, $J$ = 13.5 Hz, 1H), 3.04 (d, $J$ = 7.8 Hz, 1H), 1.25 (s, 3H), 1.20 (s, 3H). $^{13}$C NMR (75 MHz, $CDCl_3$) δ 175.6, 174.6, 136.9, 135.5, 132.8 (d, $J$ = 13.1 Hz), 131.8 (d, $J$ = 6.4 Hz), 129.9, 129.2, 128.9, 128.4, 127.2, 126.5 (d, $J$ = 14.8 Hz), 124.5, 123.9, 62.6, 62.1, 53.9, 46.2, 44.0, 24.4, 20.1. HRMS (ESI) calcd for $C_{29}H_{26}ClN_2O_2$ ([M+H]$^+$): 469.1677, found 469.1680.

## 8. $^{1}$H NMR and $^{13}$C NMR of selected pyrrolidine adduct 5 and 6

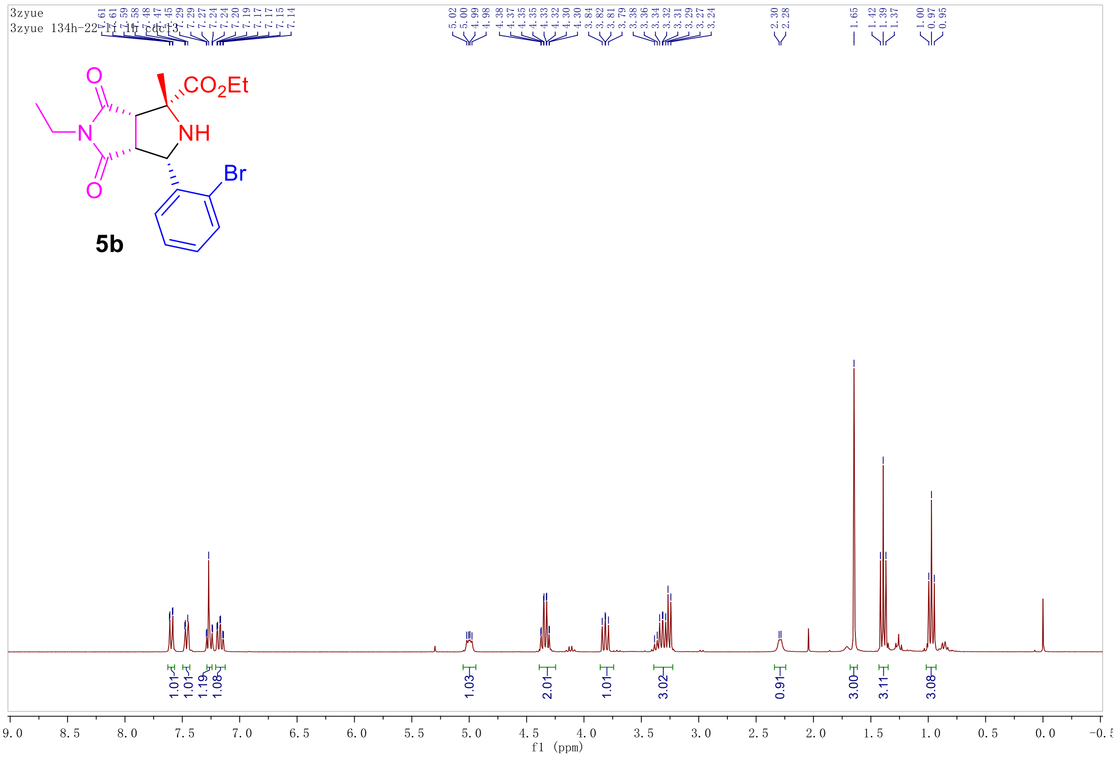

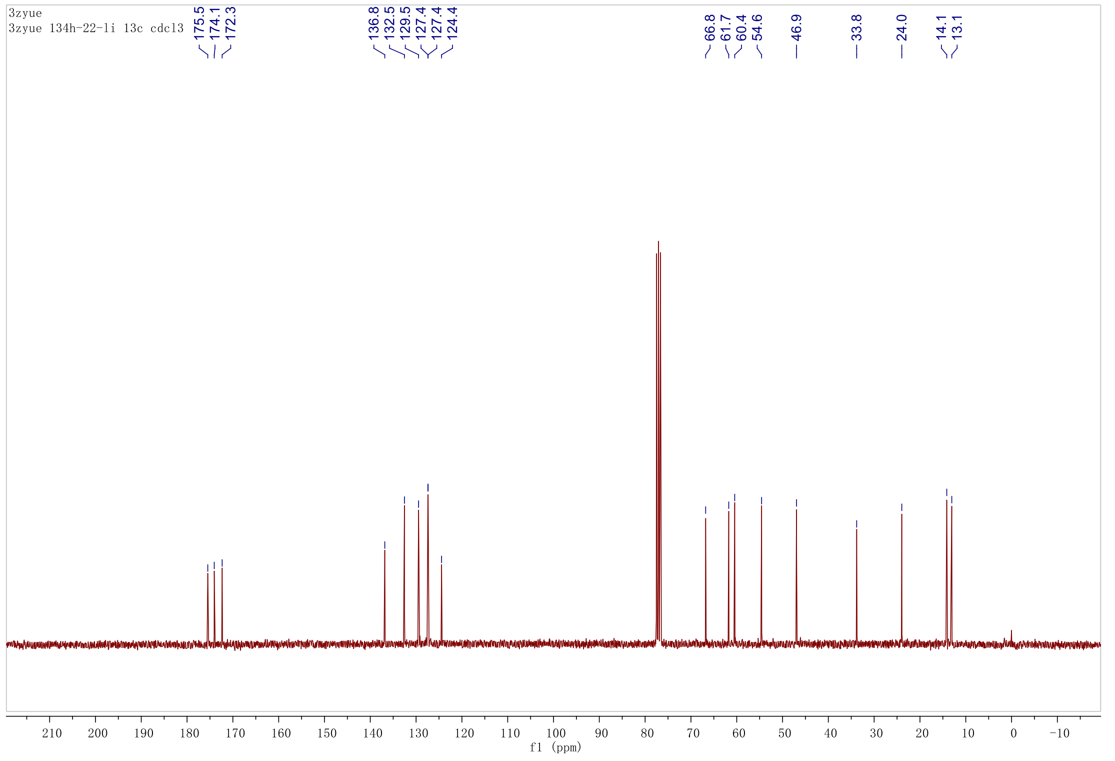

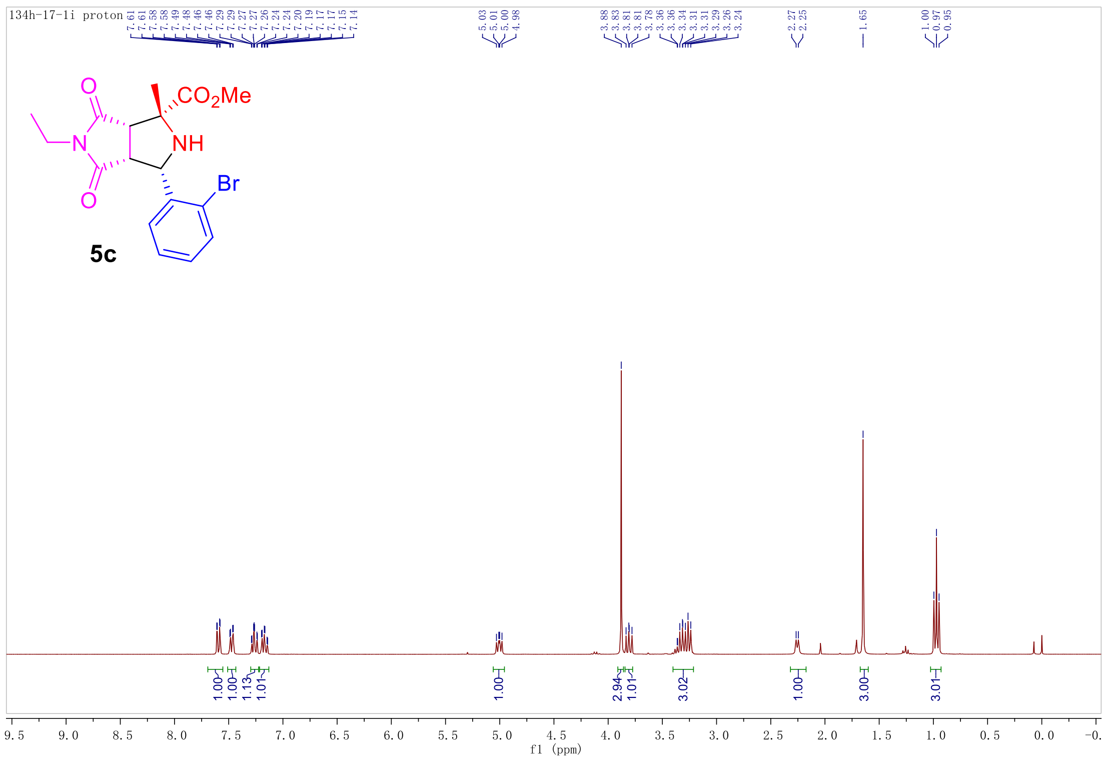
134h-17-1i proton
CO2Me
NH
Br
O
N
O
5c
1.00
1.00
1.13
1.01
1.00
2.94
1.01
3.02
1.00
3.00
3.01
f1 (ppm)

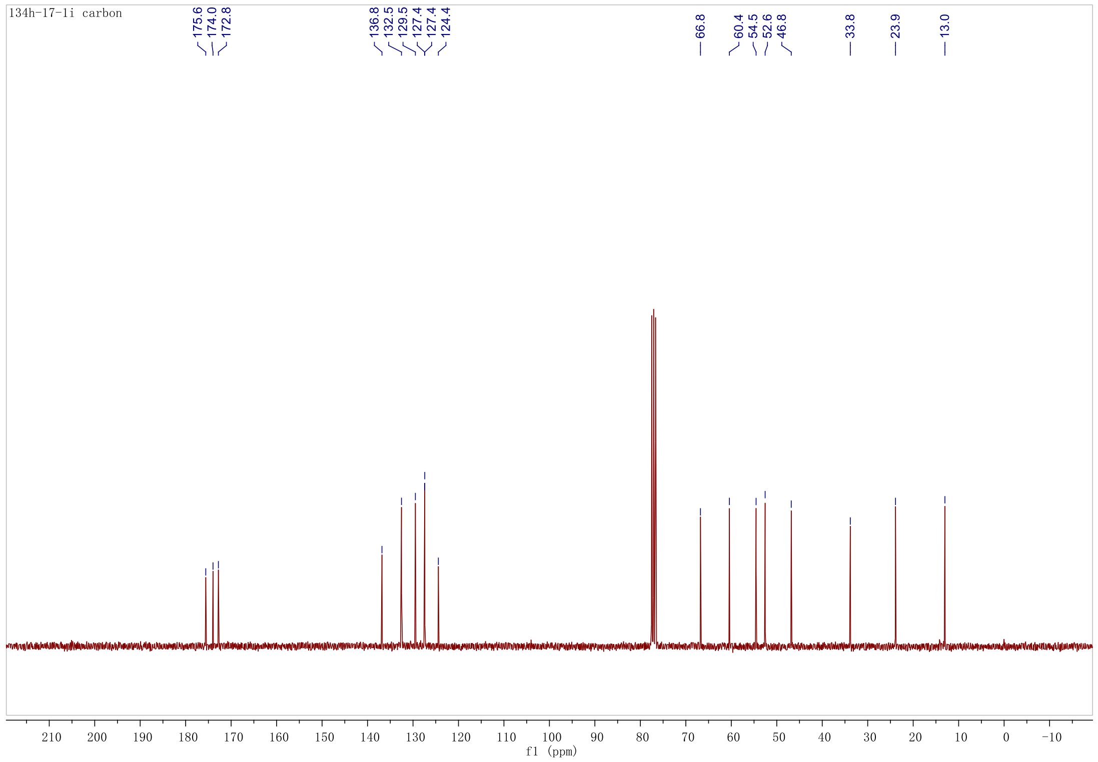
134h-17-1i carbon
175.6
174.0
172.8
136.8
132.5
129.5
127.4
127.4
124.4
66.8
60.4
54.5
52.6
46.8
33.8
23.9
13.0
f1 (ppm)

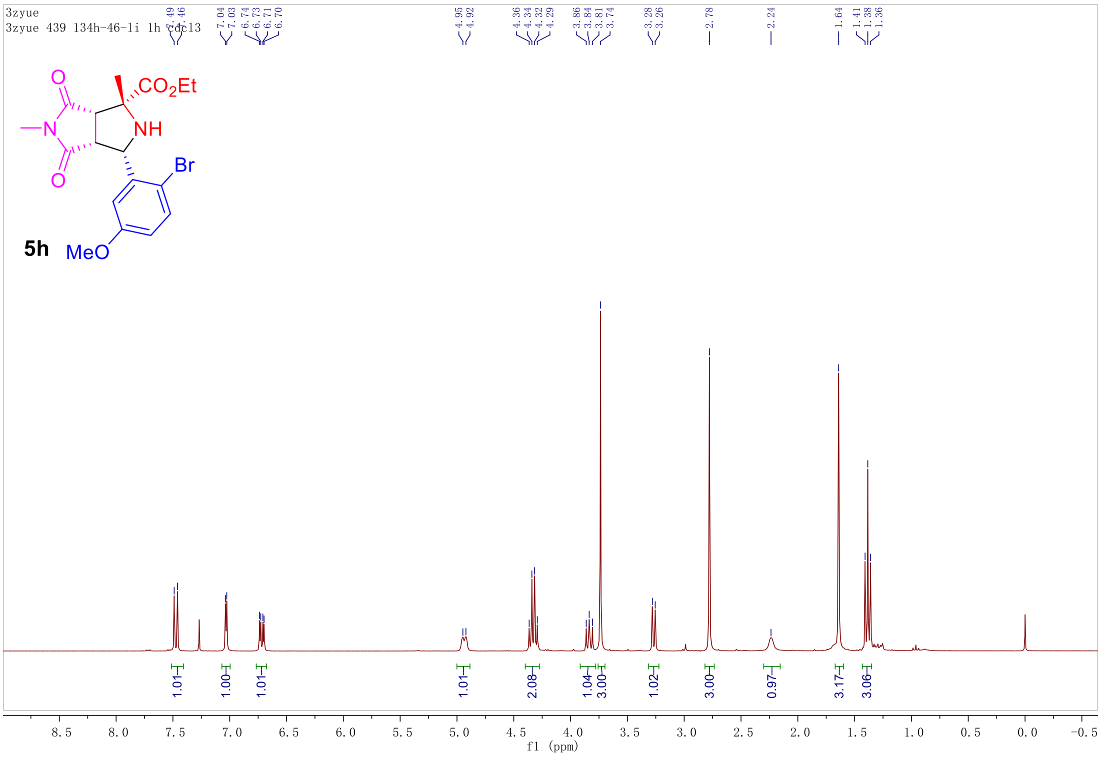
3zyue
3zyue 439 134h-46-1i 1h cdcl3
5h
MeO
Br
NH
CO2Et
7.49
7.46
7.04
7.03
6.74
6.73
6.71
6.70
4.95
4.92
4.36
4.34
4.32
4.29
3.86
3.84
3.81
3.74
3.28
3.26
2.78
2.24
1.64
1.41
1.38
1.36
1.01
1.00
1.01
1.01
2.08
1.04
3.00
1.02
3.00
0.97
3.17
3.06
f1 (ppm)

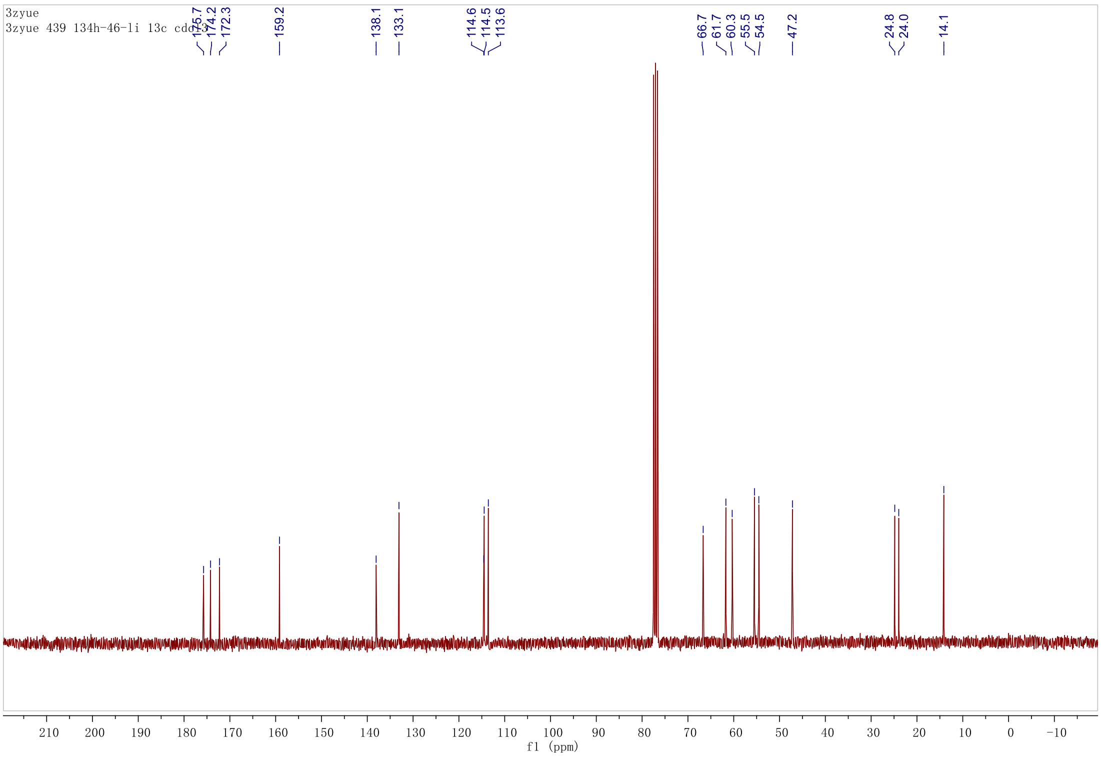
3zyue
3zyue 439 134h-46-1i 13c cdcl3
175.7
174.2
172.3
159.2
138.1
133.1
114.6
114.5
113.6
66.7
61.7
60.3
55.5
54.5
47.2
24.8
24.0
14.1
f1 (ppm)

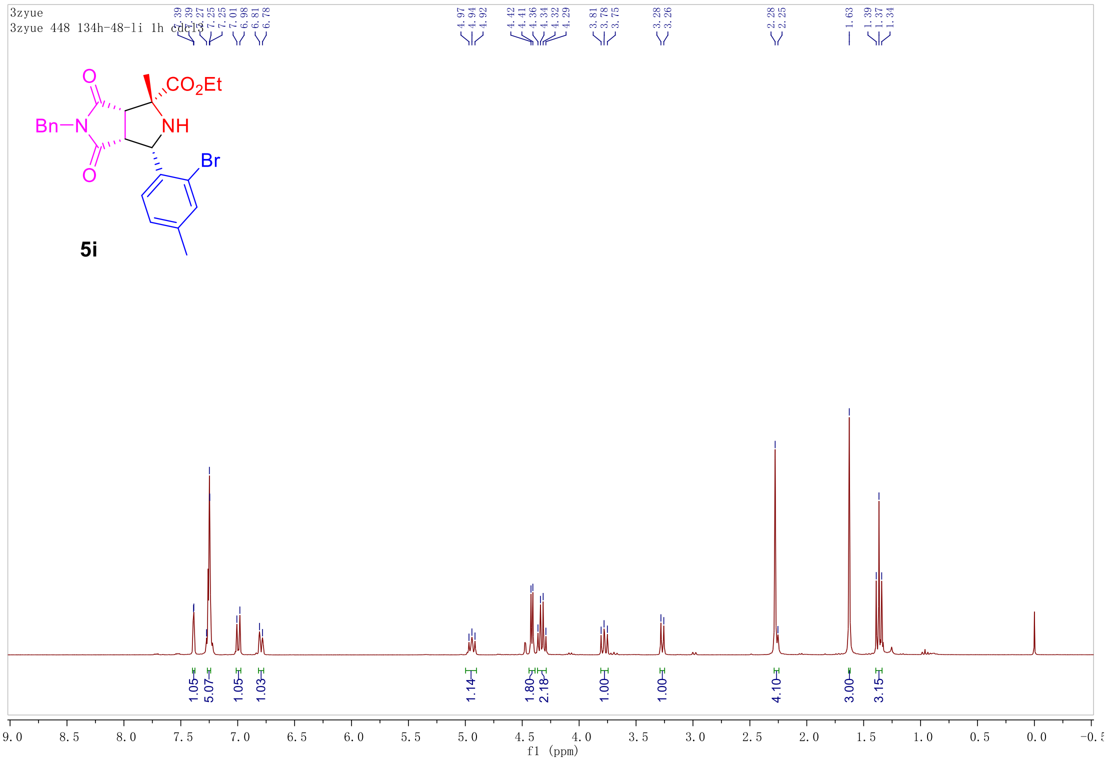
3zyue
3zyue 448 134h-48-1i 1h cdcl3
7.39
7.39
7.27
7.25
7.25
7.01
6.98
6.81
6.78
4.97
4.94
4.92
4.42
4.41
4.36
4.34
4.32
4.29
3.81
3.78
3.75
3.28
3.26
2.28
2.25
1.63
1.39
1.37
1.34
Bn
N
NH
O
O
CO2Et
Br
5i
1.05
5.07
1.05
1.03
1.14
1.80
2.18
1.00
1.00
4.10
3.00
3.15
f1 (ppm)

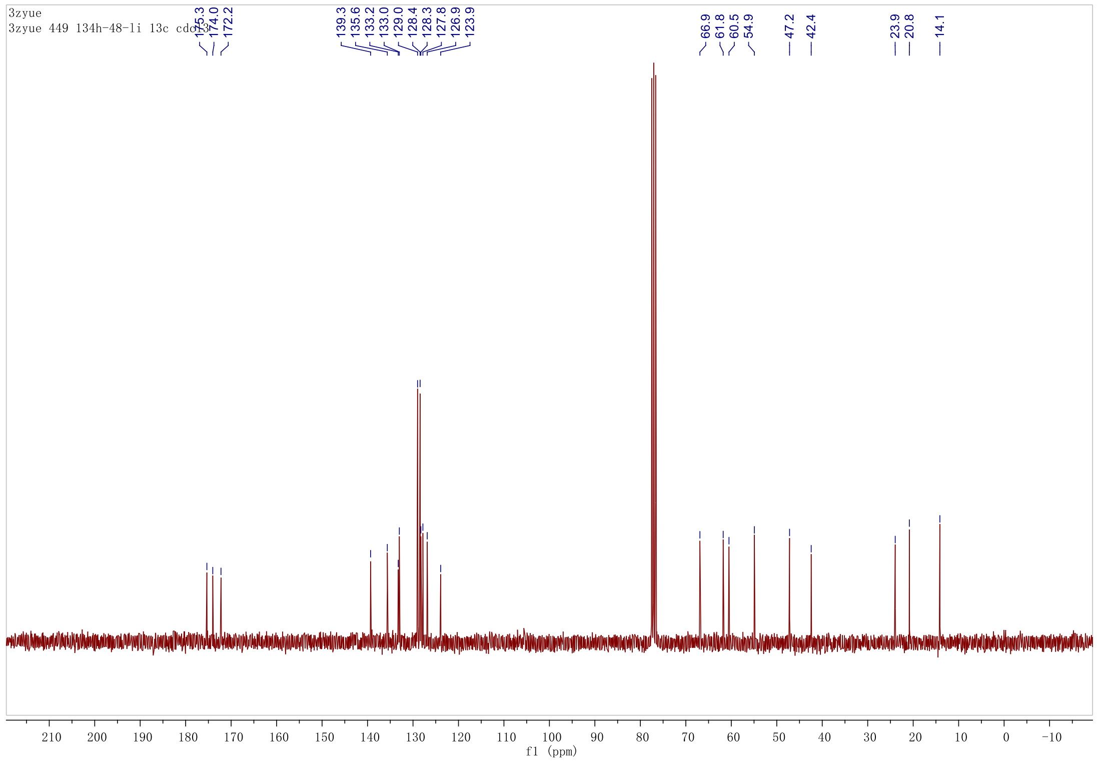
3zyue
3zyue 449 134h-48-1i 13c cdcl3
175.3
174.0
172.2
139.3
135.6
133.2
133.0
129.0
128.4
128.3
127.8
126.9
123.9
66.9
61.8
60.5
54.9
47.2
42.4
23.9
20.8
14.1
f1 (ppm)

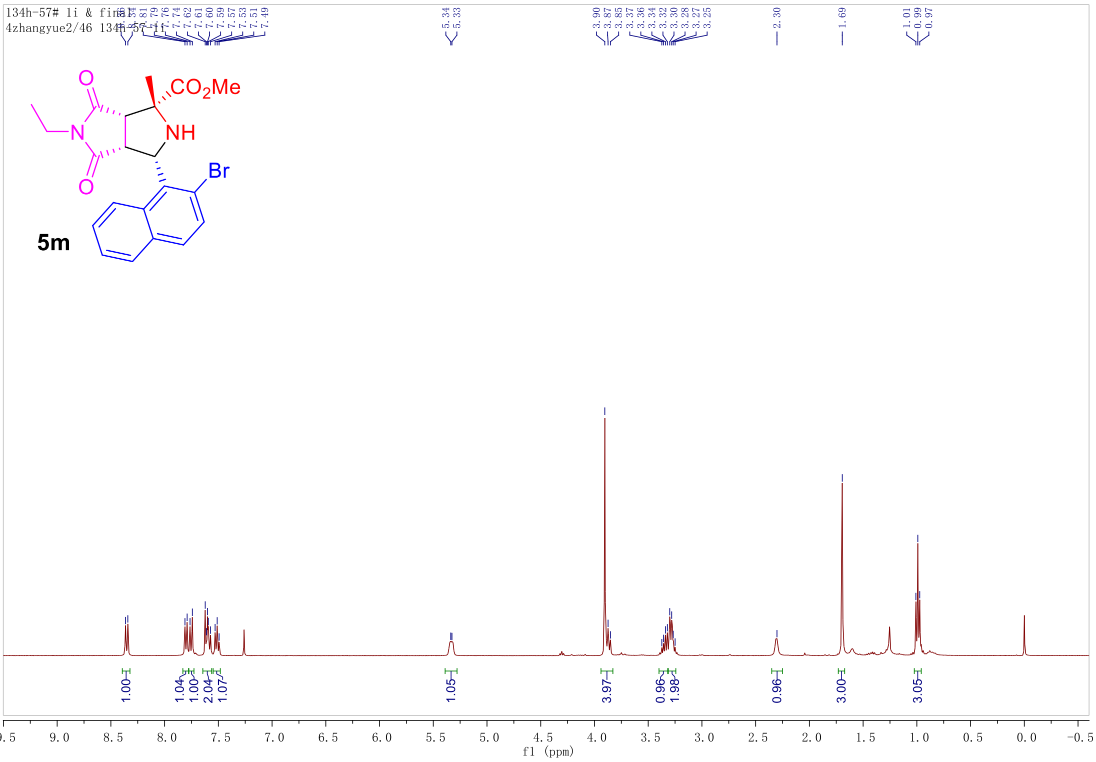
134h-57# 1i & final
4zhangyue2/46 134h-57-1i
5.34
5.33
3.90
3.87
3.85
2.30
1.69
1.01
0.99
0.97
CO2Me
NH
Br
O
N
O
5m
1.00
1.04
1.00
2.04
1.07
1.05
3.97
0.96
1.98
0.96
3.00
3.05
f1 (ppm)

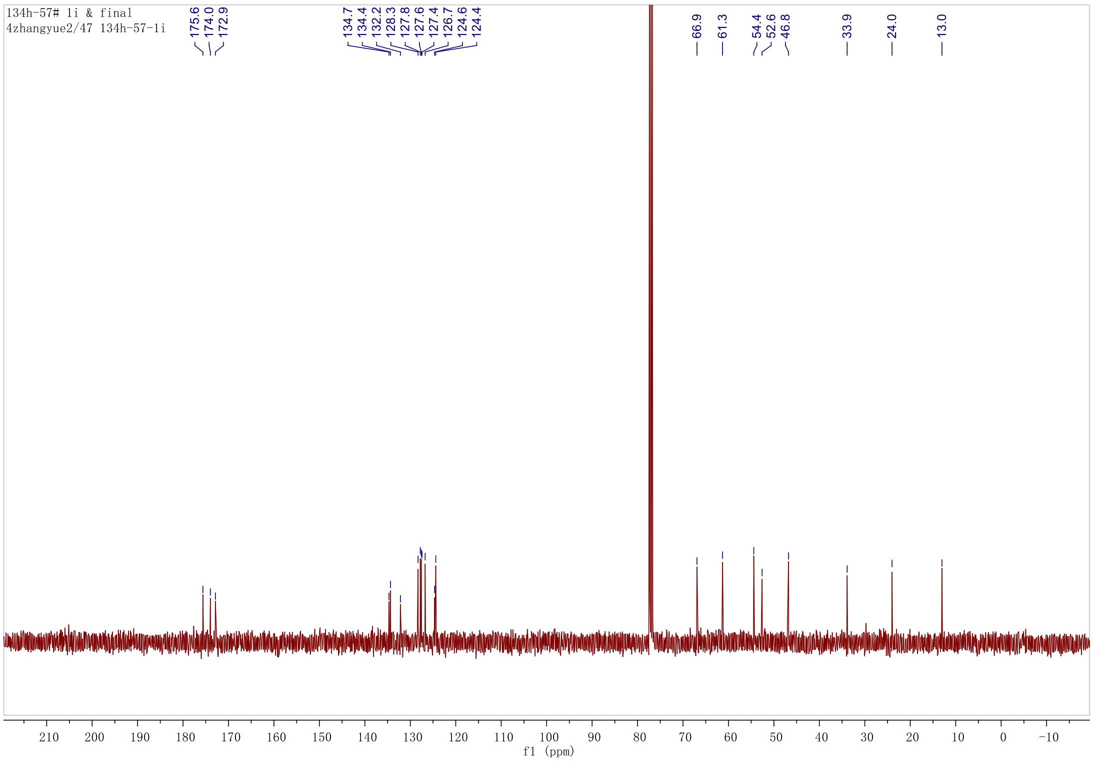
134h-57# 1i & final
4zhangyue2/47 134h-57-1i
175.6
174.0
172.9
134.7
134.4
132.2
128.3
127.8
127.6
127.4
126.7
124.6
124.4
66.9
61.3
54.4
52.6
46.8
33.9
24.0
13.0
f1 (ppm)

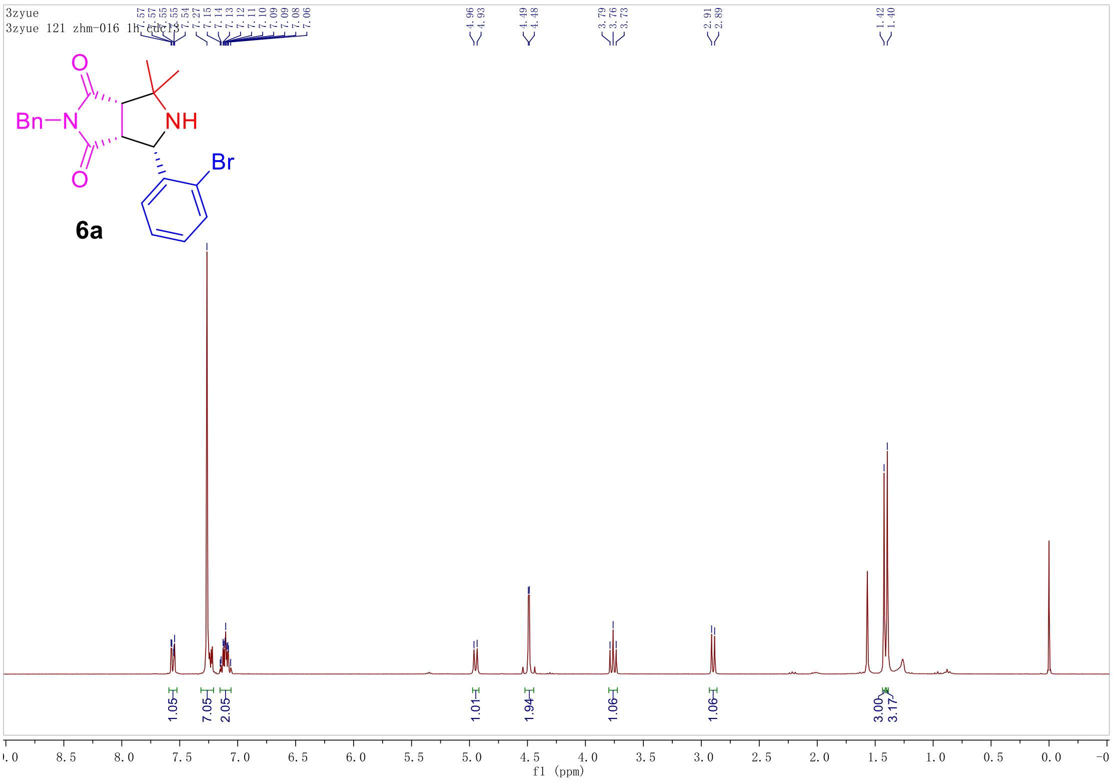
3zyue
3zyue 121 zhm-016 1h cdcl3
Bn−N
NH
Br
6a
f1 (ppm)

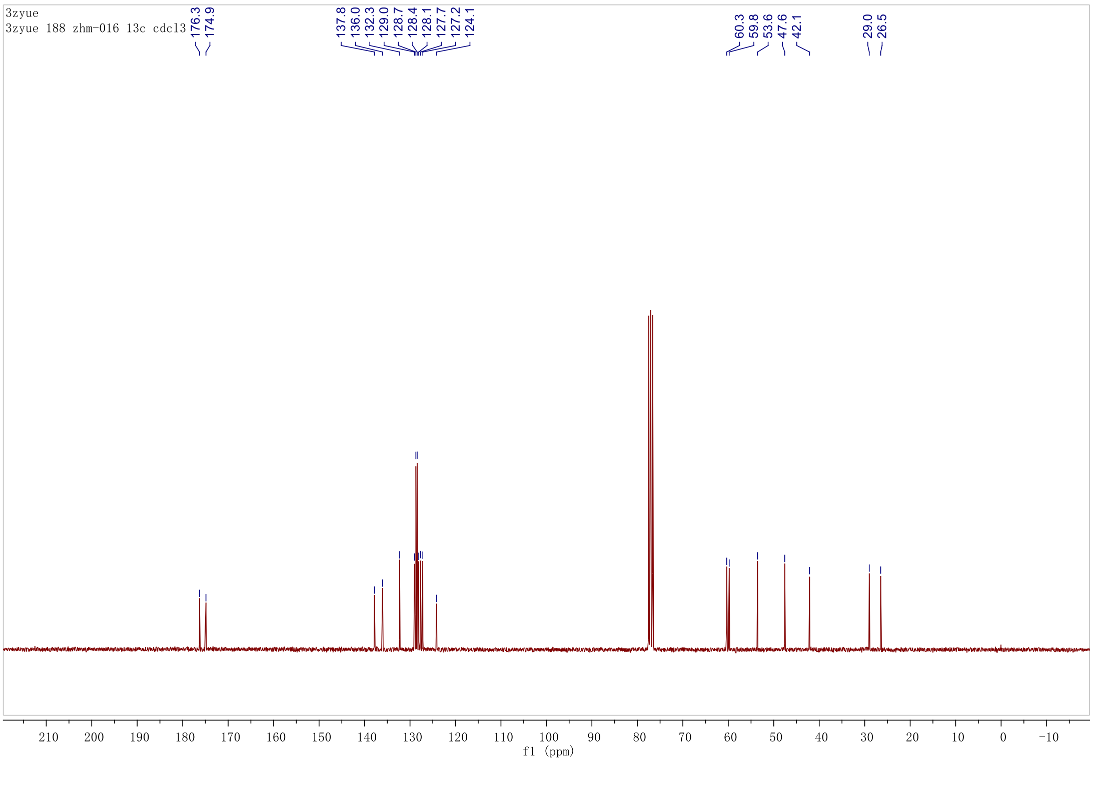
3zyue
3zyue 188 zhm-016 13c cdcl3
176.3
174.9
137.8
136.0
132.3
129.0
128.7
128.4
128.1
127.7
127.2
124.1
60.3
59.8
53.6
47.6
42.1
29.0
26.5
f1 (ppm)

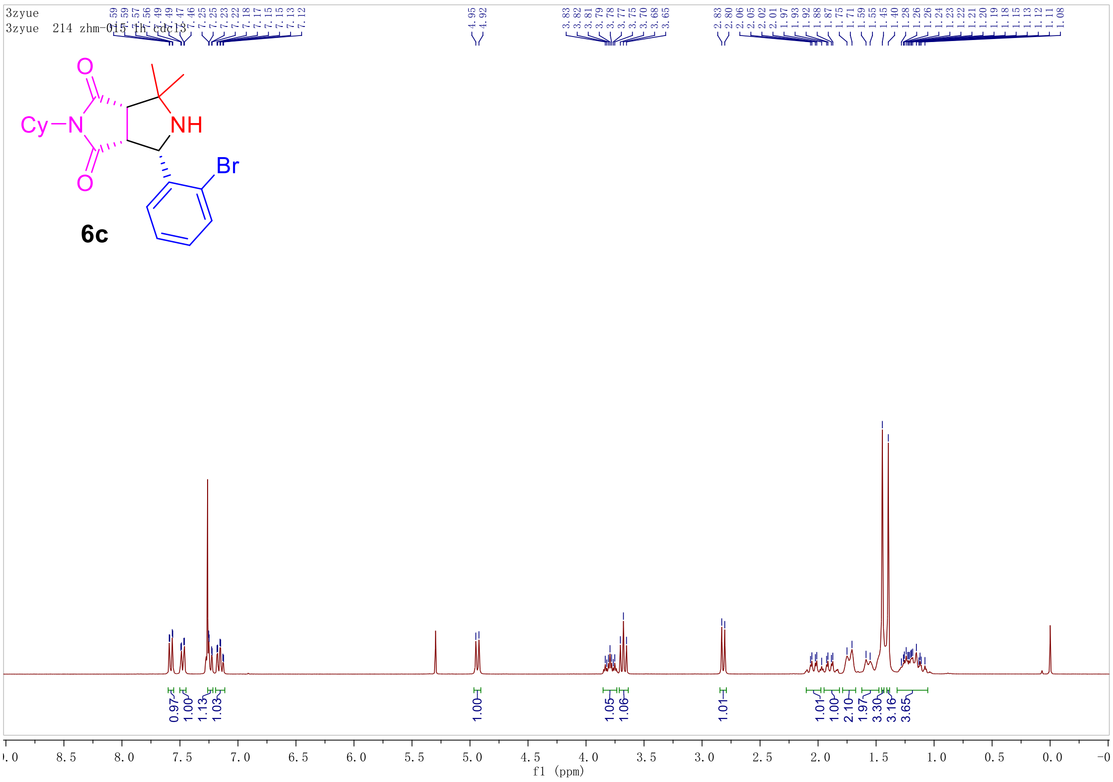

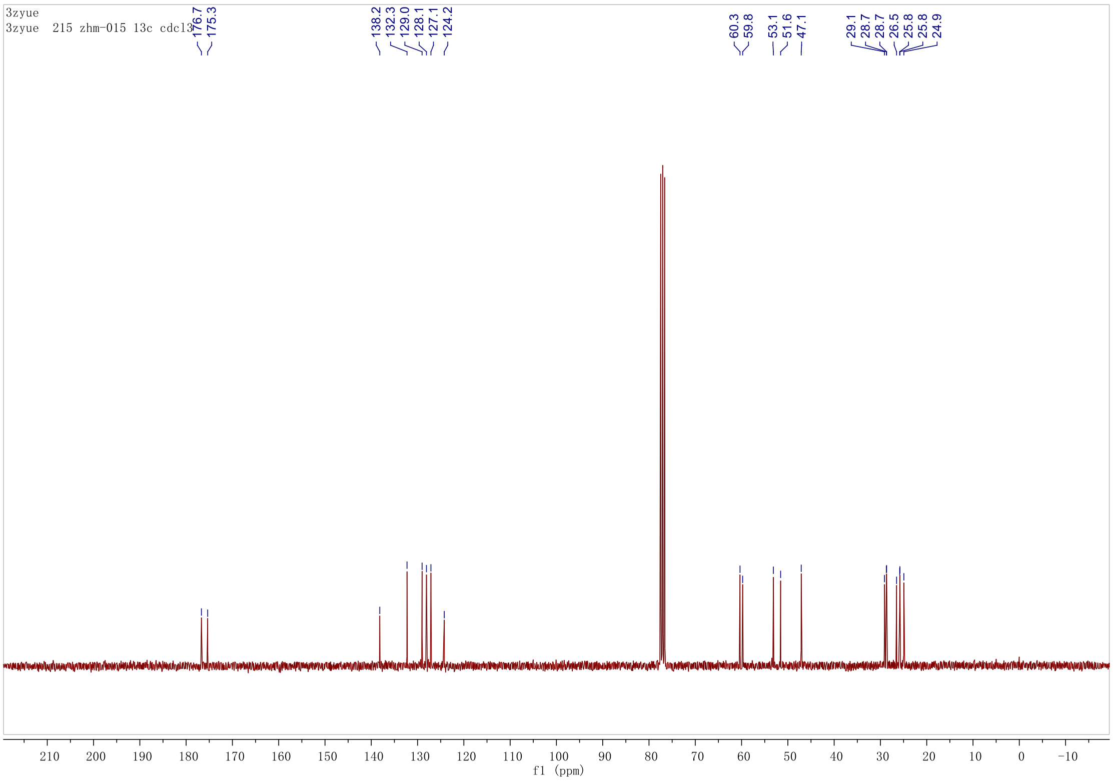

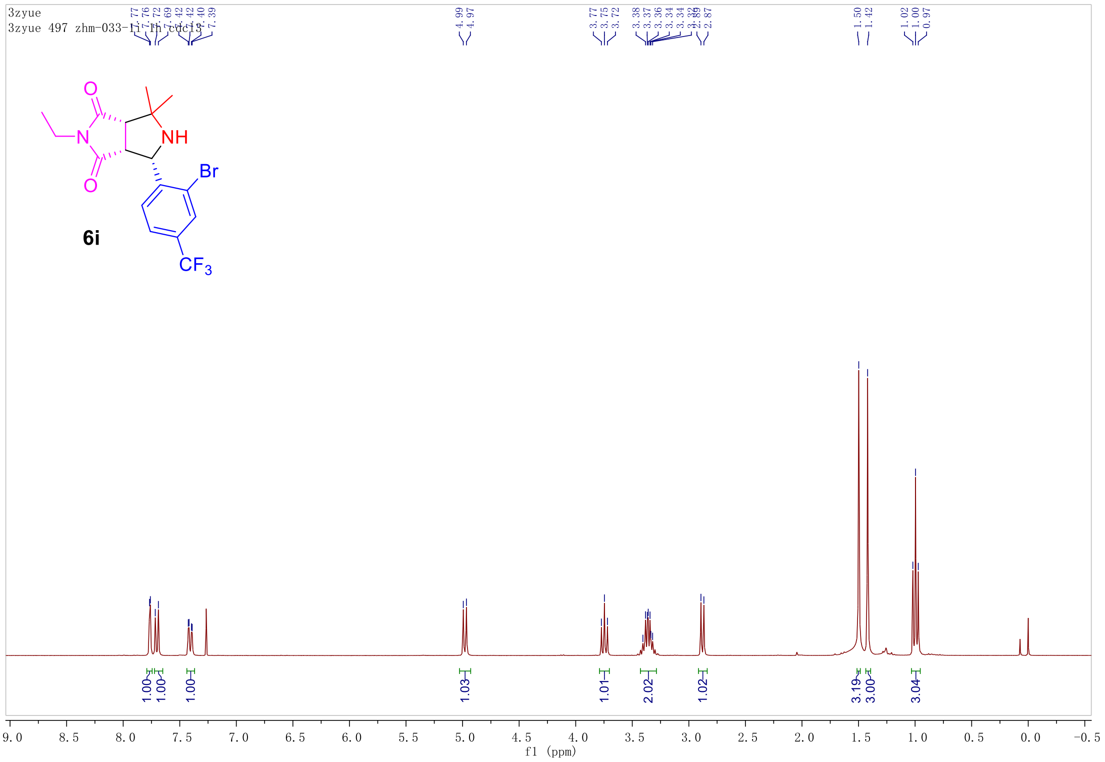
3zyue
NH
Br
O
N
CF3
6i
7.77
7.76
7.72
7.69
4.99
4.97
3.77
3.75
3.72
3.38
3.37
3.36
3.34
3.34
2.87
1.50
1.42
1.02
1.00
0.97
1.00
1.00
1.00
1.03
1.01
2.02
1.02
3.19
3.00
3.04
f1 (ppm)

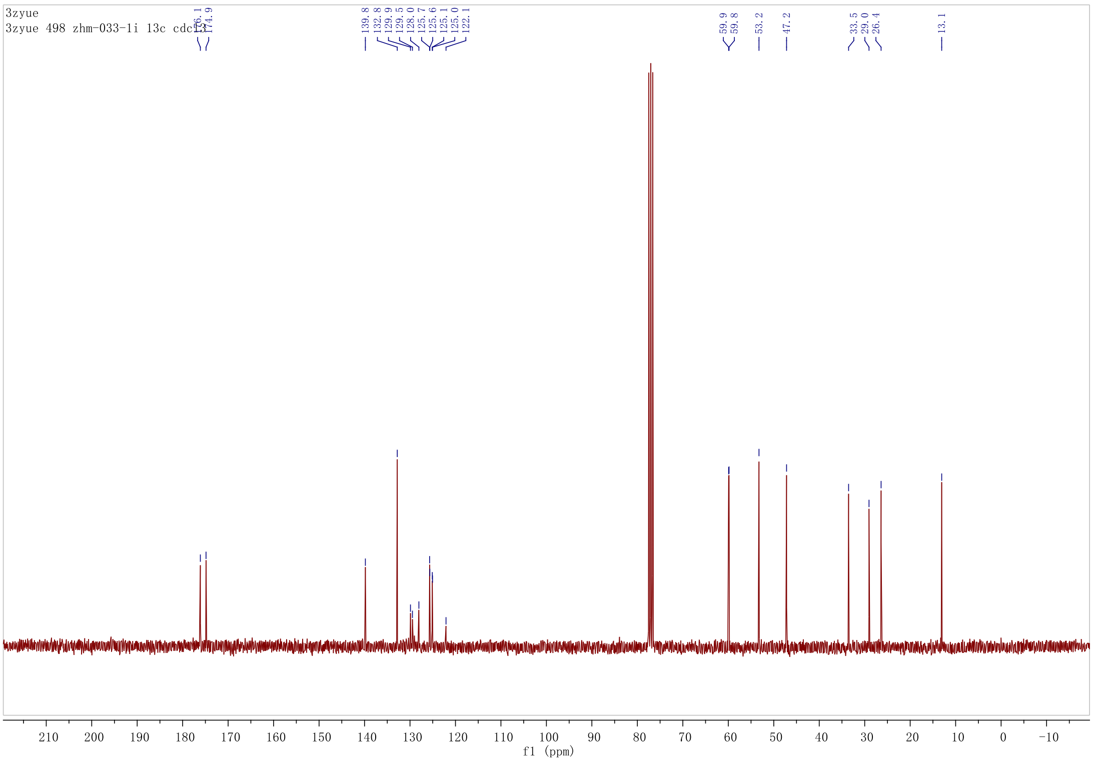
3zyue
3zyue 498 zhm-033-1i 13c cdcl3
139.8
132.8
129.9
129.5
128.0
125.7
125.6
125.1
125.0
122.1
59.9
59.8
53.2
47.2
33.5
29.0
26.4
13.1
f1 (ppm)

## 9. $^{1}$H NMR, $^{13}$C NMR and $^{19}$F NMR of products 9, 11 and 12

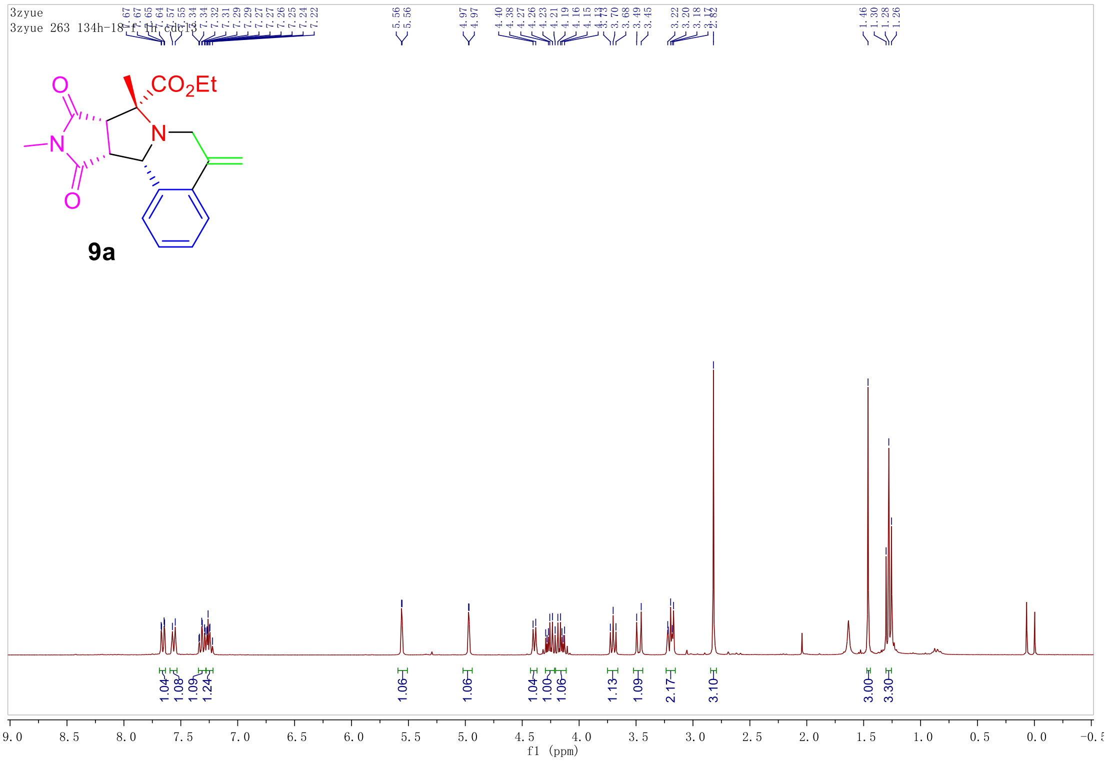


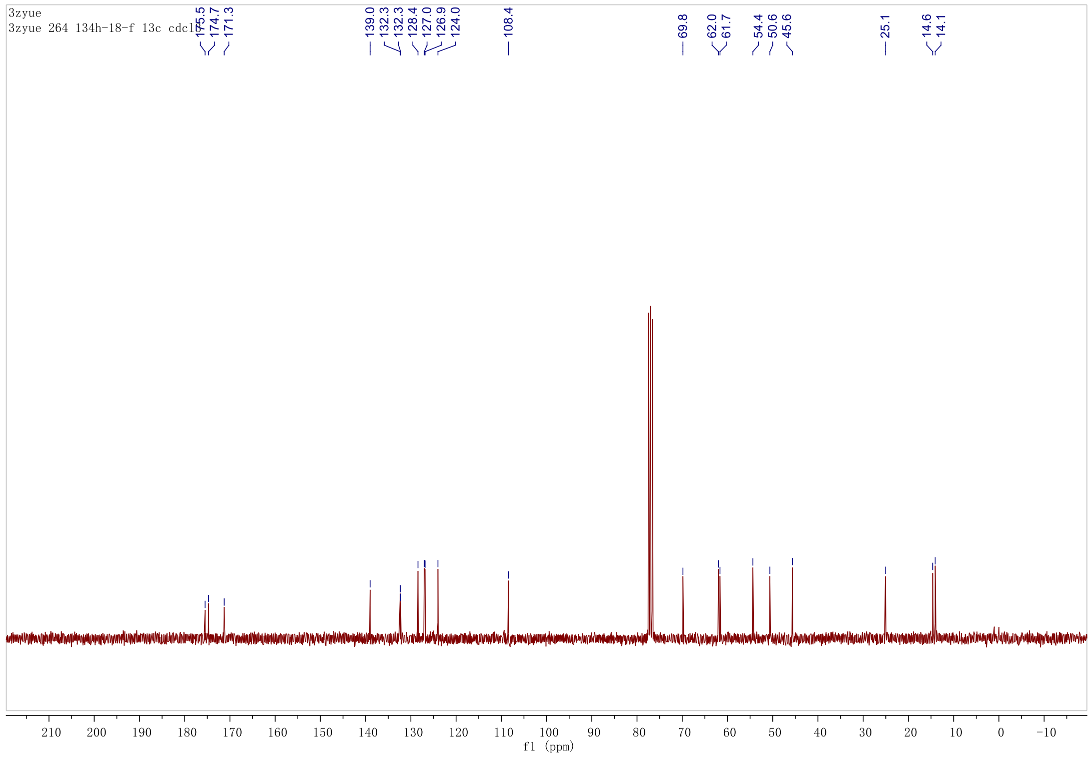

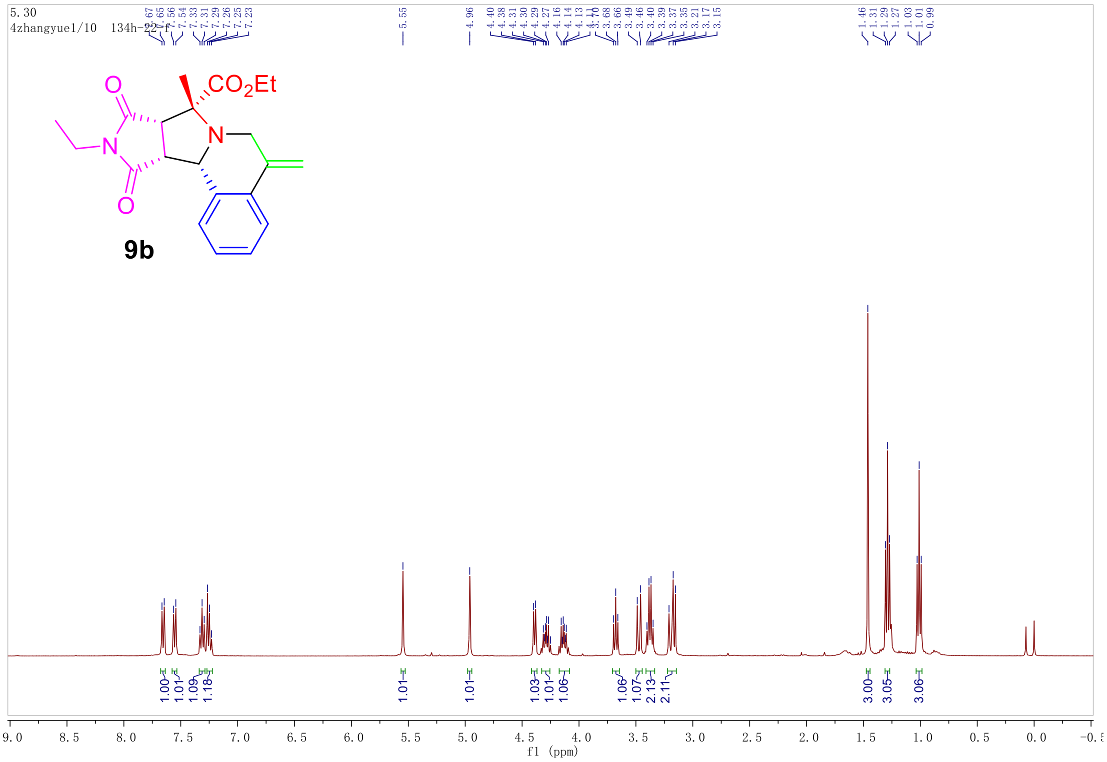

5.30
4zhangyue1/10 134h-22-f
CO2Et
O
N
N
O
9b
f1 (ppm)


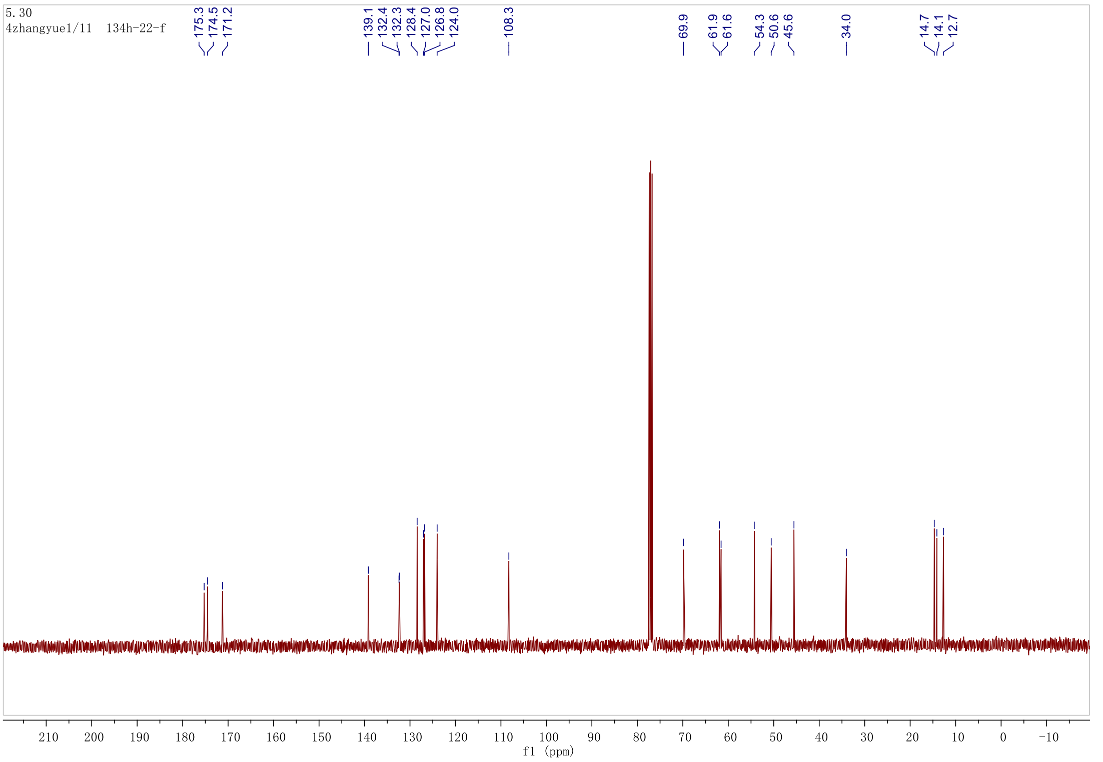

5.30
4zhangyue1/11 134h-22-f
175.3
174.5
171.2
139.1
132.4
132.3
128.4
127.0
126.8
124.0
108.3
69.9
61.9
61.6
54.3
50.6
45.6
34.0
14.7
14.1
12.7
f1 (ppm)

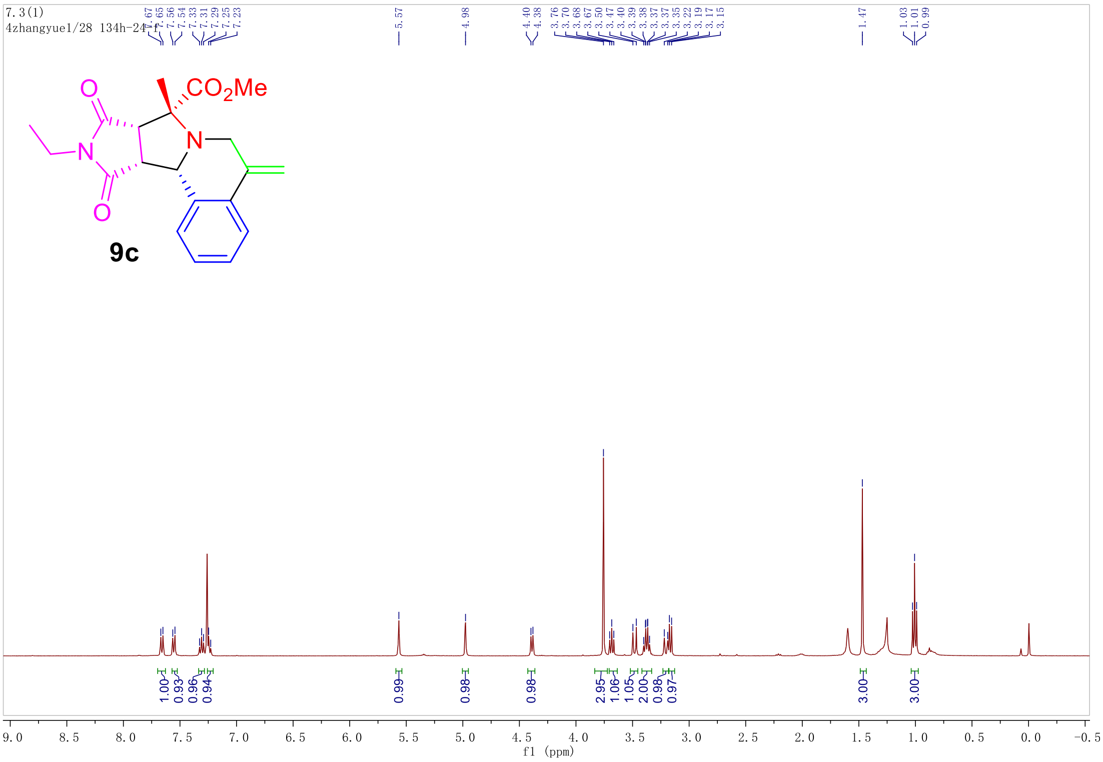
7.3(1)
4zhangyue1/28 134h-24-f
7.67 7.65 7.56 7.54 7.33 7.31 7.29 7.25 7.23
5.57
4.98
4.40 4.38
3.76 3.70 3.68 3.67 3.50 3.47 3.40 3.39 3.38 3.37 3.35 3.22 3.19 3.17 3.15
1.47
1.03 1.01 0.99
CO2Me
N
O
9c
1.00 0.93 0.96 0.94
0.99
0.98
0.98
2.95 1.06 1.05 2.00 0.98 0.97
3.00
3.00
f1 (ppm)

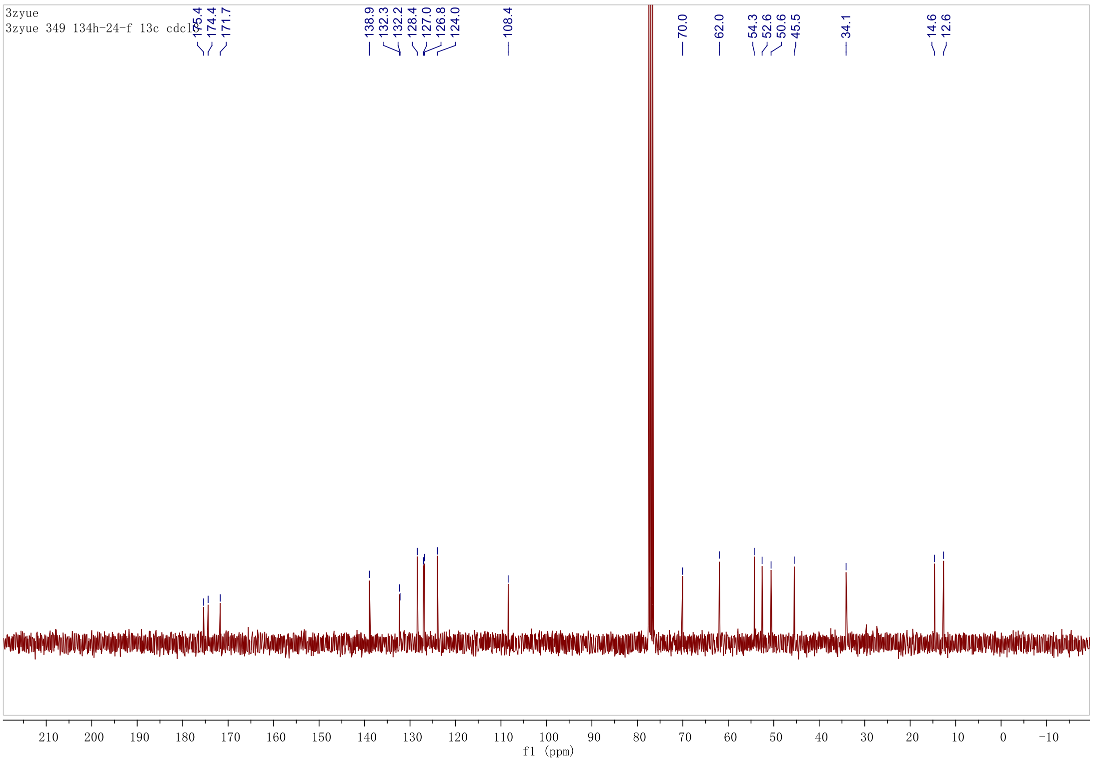
3zyue
3zyue 349 134h-24-f 13c cdcl3
175.4 174.4 171.7
138.9 132.3 132.2 128.4 127.0 126.8 124.0
108.4
70.0
62.0
54.3 52.6 50.6 45.5
34.1
14.6 12.6
f1 (ppm)

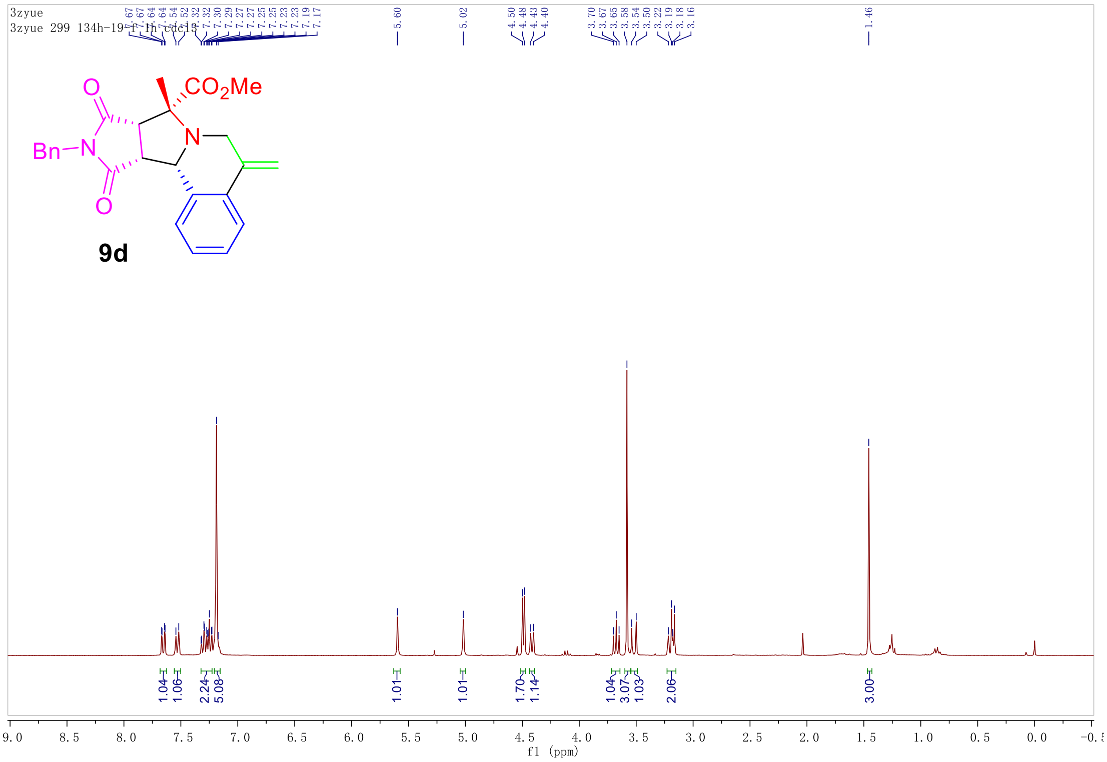
3zyue
3zyue 299 134h-19-f 1h cdcl3
7.67 7.67 7.64 7.64 7.54 7.52 7.32 7.32 7.30 7.29 7.27 7.27 7.25 7.25 7.23 7.23 7.19 7.17
5.60
5.02
4.50 4.48 4.43 4.40
3.70 3.67 3.65 3.58 3.54 3.50 3.22 3.19 3.18 3.16
1.46
$CO_2Me$
Bn
N
O
9d
1.04 1.06 2.24 5.08 1.01 1.01 1.70 1.14 1.04 3.07 1.03 2.06 3.00
f1 (ppm)

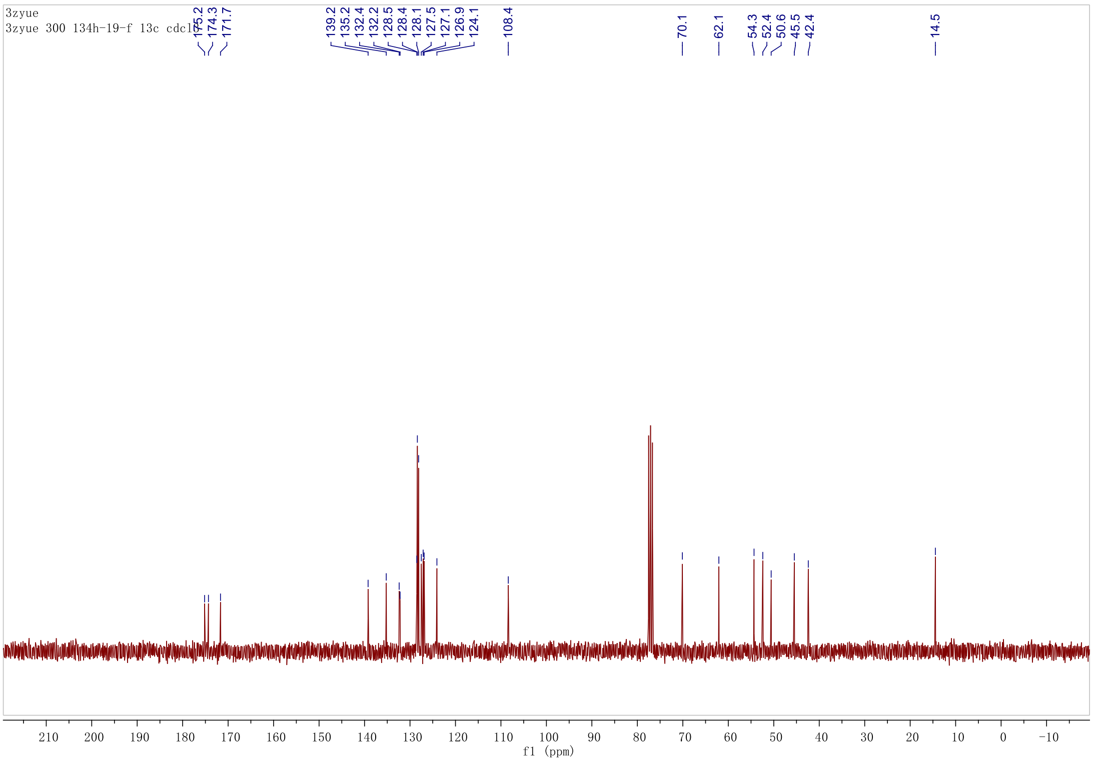
3zyue
3zyue 300 134h-19-f 13c cdcl3
175.2 174.3 171.7
139.2 135.2 132.4 132.2 128.5 128.4 128.1 127.5 127.1 126.9 124.1
108.4
70.1
62.1
54.3 52.4 50.6 45.5 42.4
14.5
f1 (ppm)

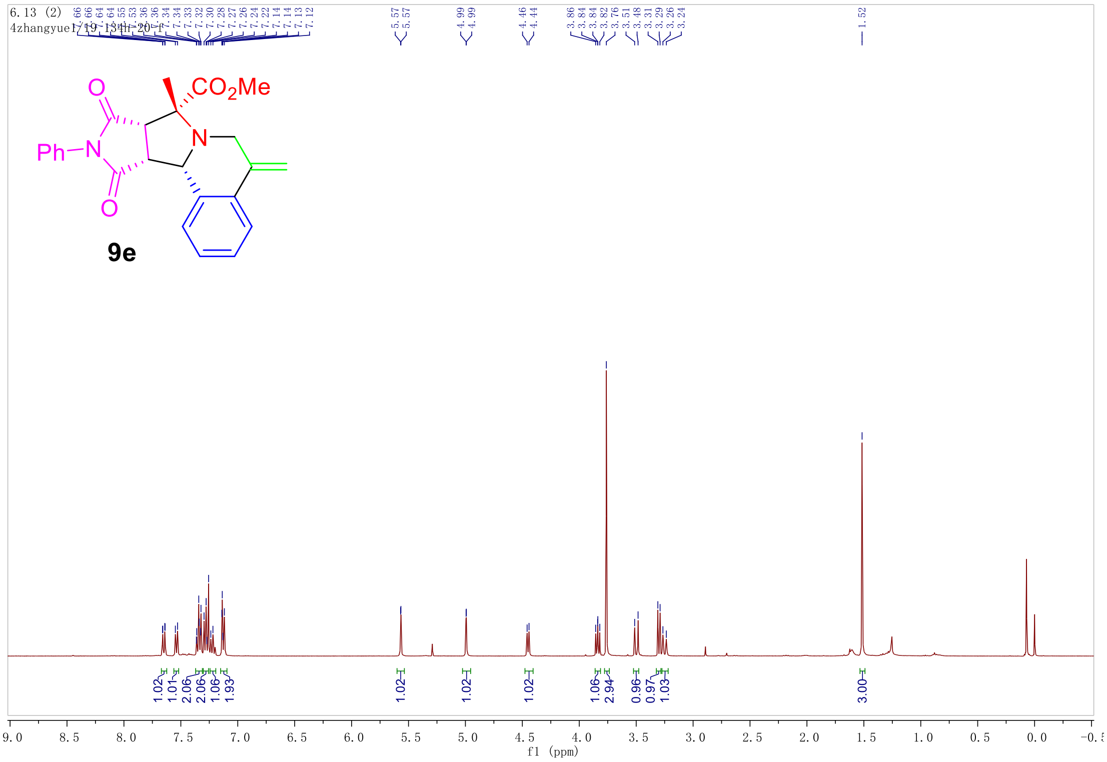
6.13 (2)
4zhangyue
9e
CO2Me
Ph
N
O
f1 (ppm)

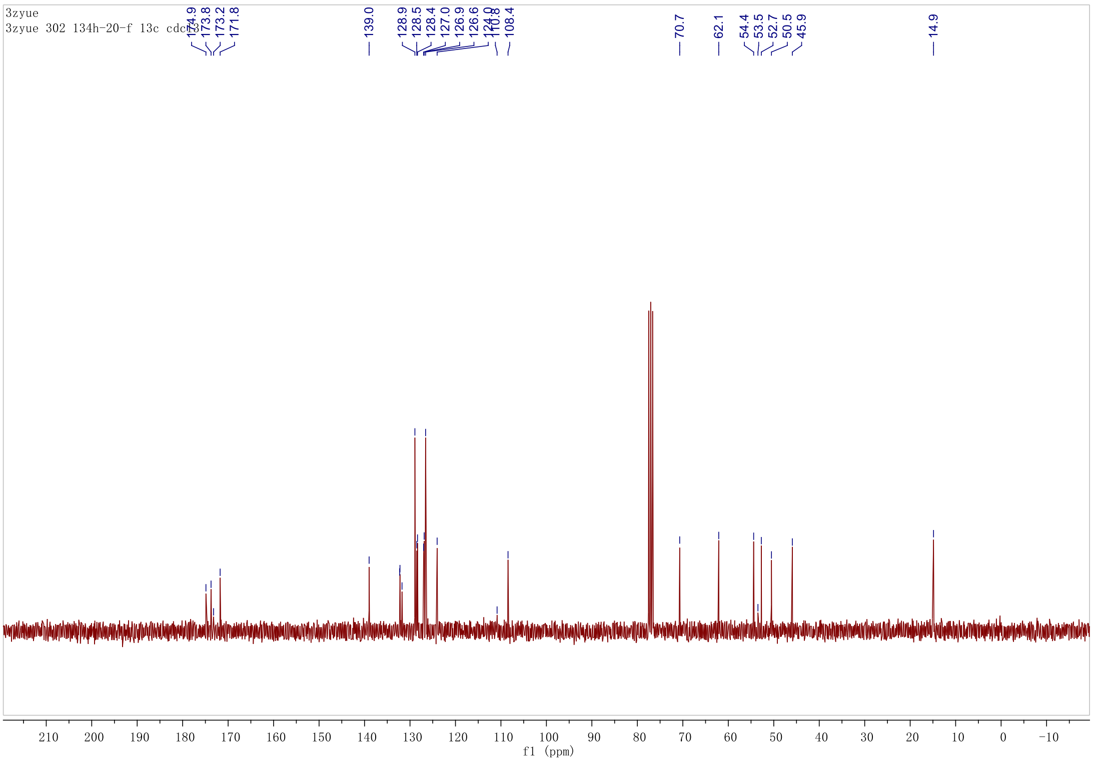
3zyue
3zyue 302 134h-20-f 13c cdcl3
f1 (ppm)

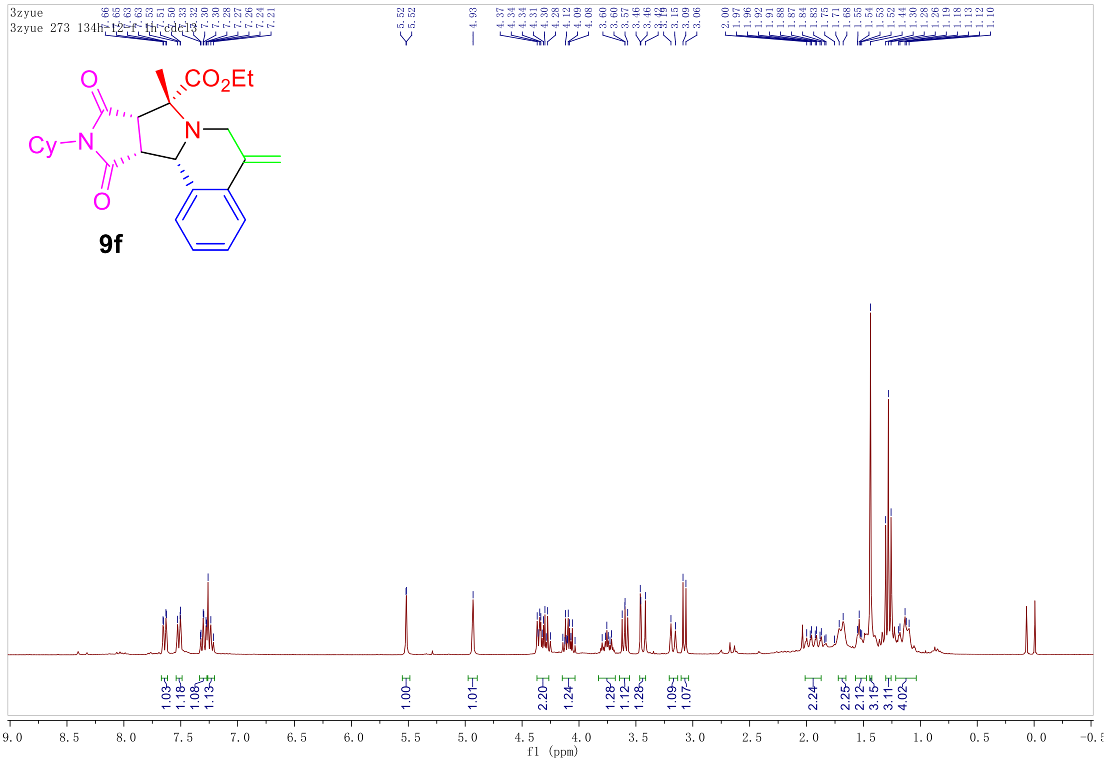
3zyue
CO2Et
Cy
N
O
9f
1.03
1.18
1.08
1.13
1.00
1.01
2.20
1.24
1.28
1.12
1.28
1.09
1.07
2.24
2.25
2.12
3.15
3.11
4.02
f1 (ppm)

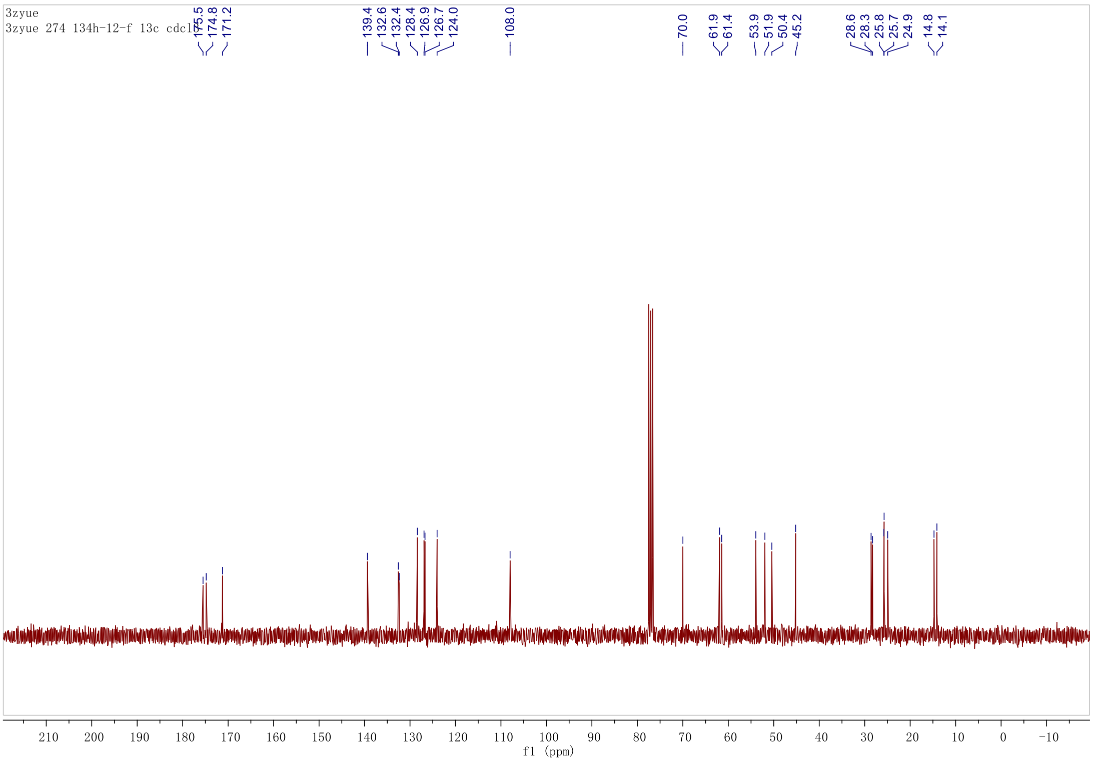
3zyue
3zyue 274 134h-12-f 13c cdcl3
175.5
174.8
171.2
139.4
132.6
132.4
128.4
126.9
126.7
124.0
108.0
70.0
61.9
61.4
53.9
51.9
50.4
45.2
28.6
28.3
25.8
25.7
24.9
14.8
14.1
f1 (ppm)

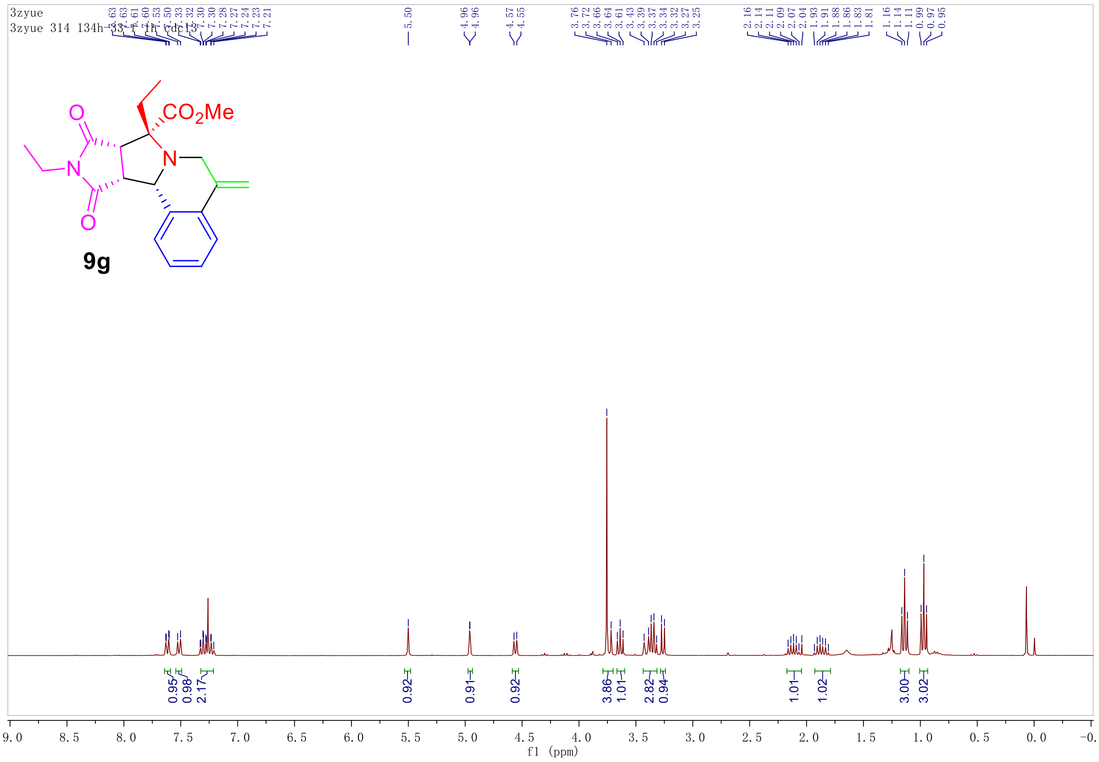
3zyue
3zyue 314 134h-33-f 1h cdcl3
CO2Me
9g
0.95
0.98
2.17
0.92
0.91
0.92
3.86
1.01
2.82
0.94
1.01
1.02
3.00
3.02
f1 (ppm)

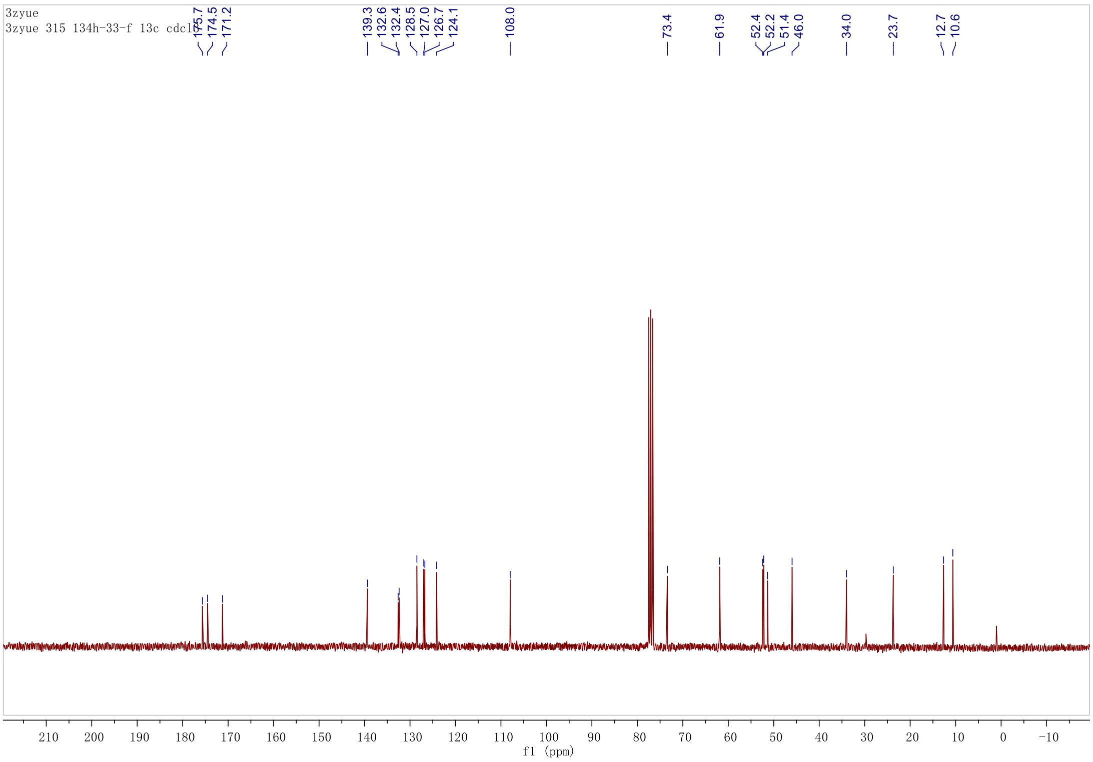
3zyue
3zyue 315 134h-33-f 13c cdcl3
175.7
174.5
171.2
139.3
132.6
132.4
128.5
127.0
126.7
124.1
108.0
73.4
61.9
52.4
52.2
51.4
46.0
34.0
23.7
12.7
10.6
f1 (ppm)

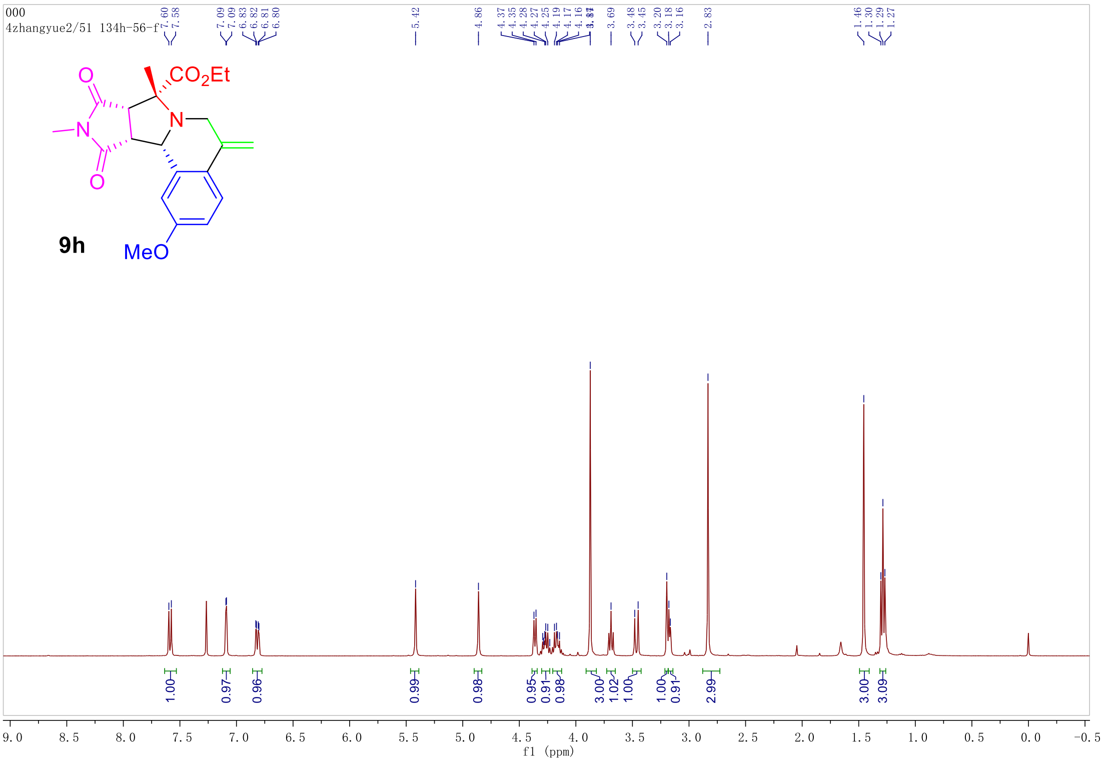
000
4zhangyue2/51 134h-56-f
7.60 7.58 7.09 7.09 6.83 6.82 6.81 6.80 5.42 4.86 4.37 4.35 4.28 4.27 4.25 4.19 4.17 4.16 3.69 3.48 3.45 3.20 3.18 3.16 2.83 1.46 1.30 1.29 1.27
CO2Et
N
O
O
MeO
9h
1.00 0.97 0.96 0.99 0.98 0.95 0.91 0.98 3.00 1.02 1.00 1.00 0.91 2.99 3.00 3.09
9.0 8.5 8.0 7.5 7.0 6.5 6.0 5.5 5.0 4.5 4.0 3.5 3.0 2.5 2.0 1.5 1.0 0.5 0.0 -0.5
f1 (ppm)

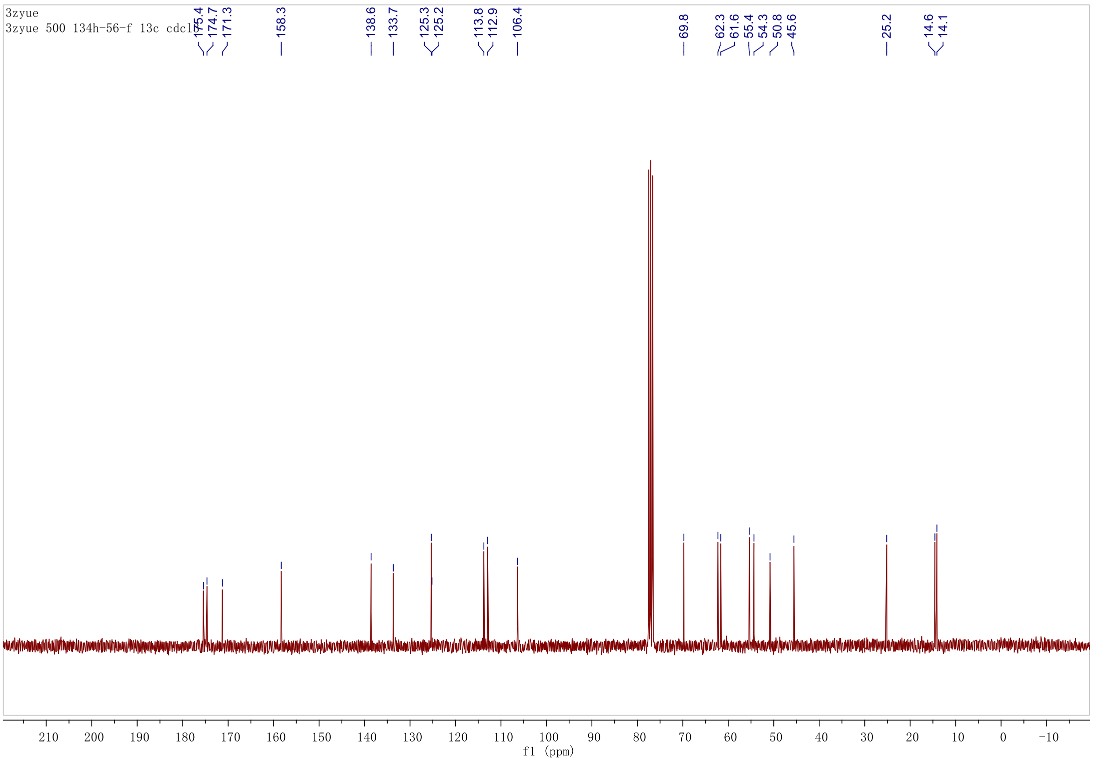
3zyue
3zyue 500 134h-56-f 13c cdcl3
175.4 174.7 171.3 158.3 138.6 133.7 125.3 125.2 113.8 112.9 106.4 69.8 62.3 61.6 55.4 54.3 50.8 45.6 25.2 14.6 14.1
210 200 190 180 170 160 150 140 130 120 110 100 90 80 70 60 50 40 30 20 10 0 -10
f1 (ppm)

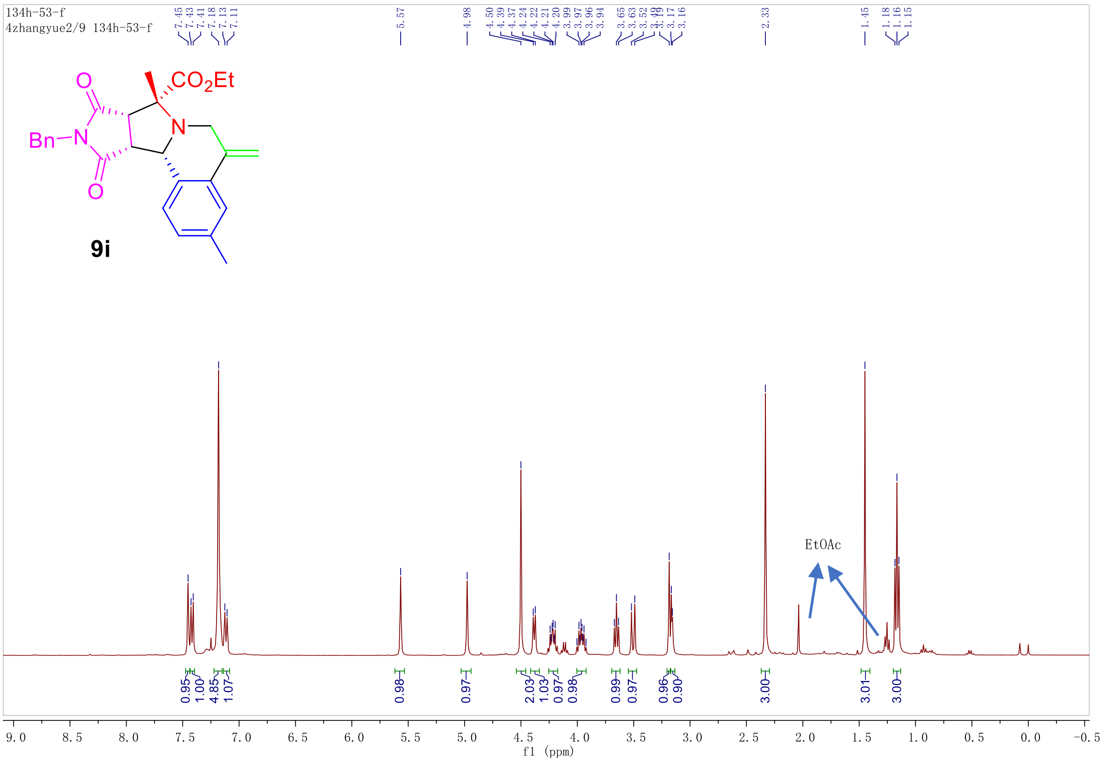
134h-53-f
4zhangyue2/9 134h-53-f
7.45 7.43 7.41 7.18 7.13 7.11
5.57
4.98
4.50 4.39 4.37 4.24 4.22 4.21 4.20 3.99 3.97 3.96 3.94
3.65 3.63 3.52 3.17 3.16
2.33
1.45 1.18 1.16 1.15
CO2Et
Bn–N
N
O
O
9i
EtOAc
0.95 1.00 4.85 1.07 0.98 0.97 2.03 1.03 0.97 0.98 0.99 0.97 0.96 0.90 3.00 3.01 3.00
9.0 8.5 8.0 7.5 7.0 6.5 6.0 5.5 5.0 4.5 4.0 3.5 3.0 2.5 2.0 1.5 1.0 0.5 0.0 -0.5
f1 (ppm)

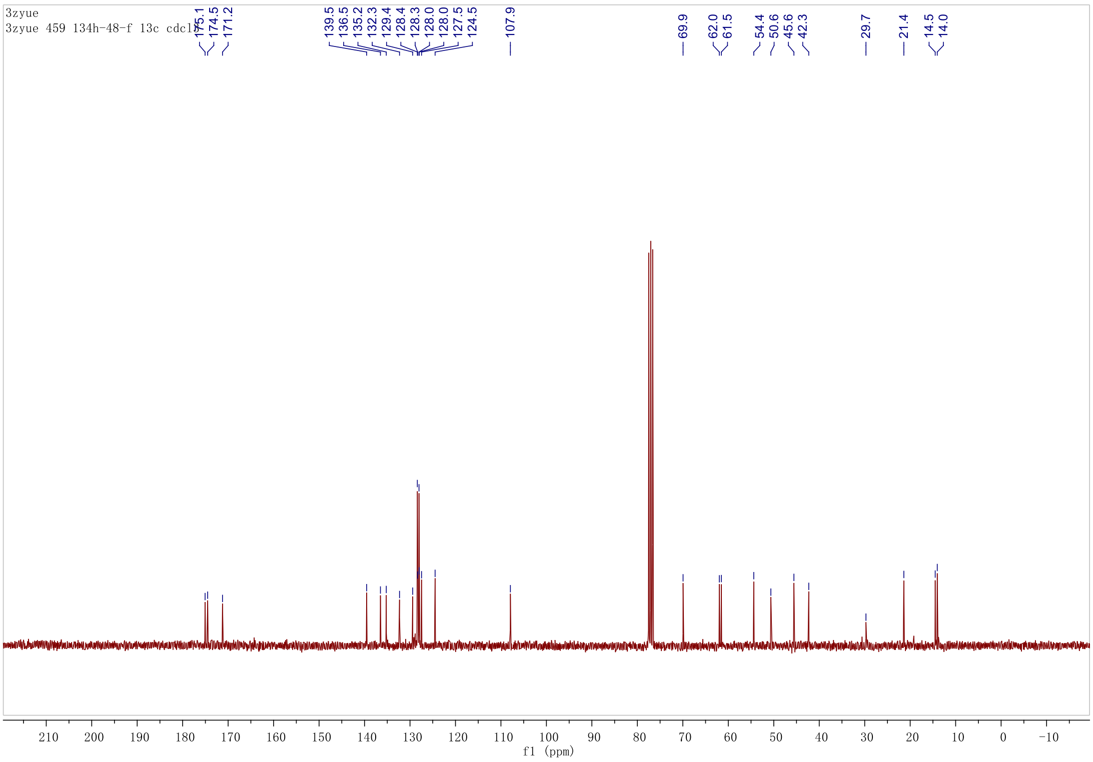
3zyue
3zyue 459 134h-48-f 13c cdcl3
175.1 174.5 171.2
139.5 136.5 135.2 132.3 129.4 128.4 128.3 128.0 128.0 127.5 124.5
107.9
69.9
62.0 61.5
54.4 50.6 45.6 42.3
29.7
21.4
14.5 14.0
210 200 190 180 170 160 150 140 130 120 110 100 90 80 70 60 50 40 30 20 10 0 -10
f1 (ppm)

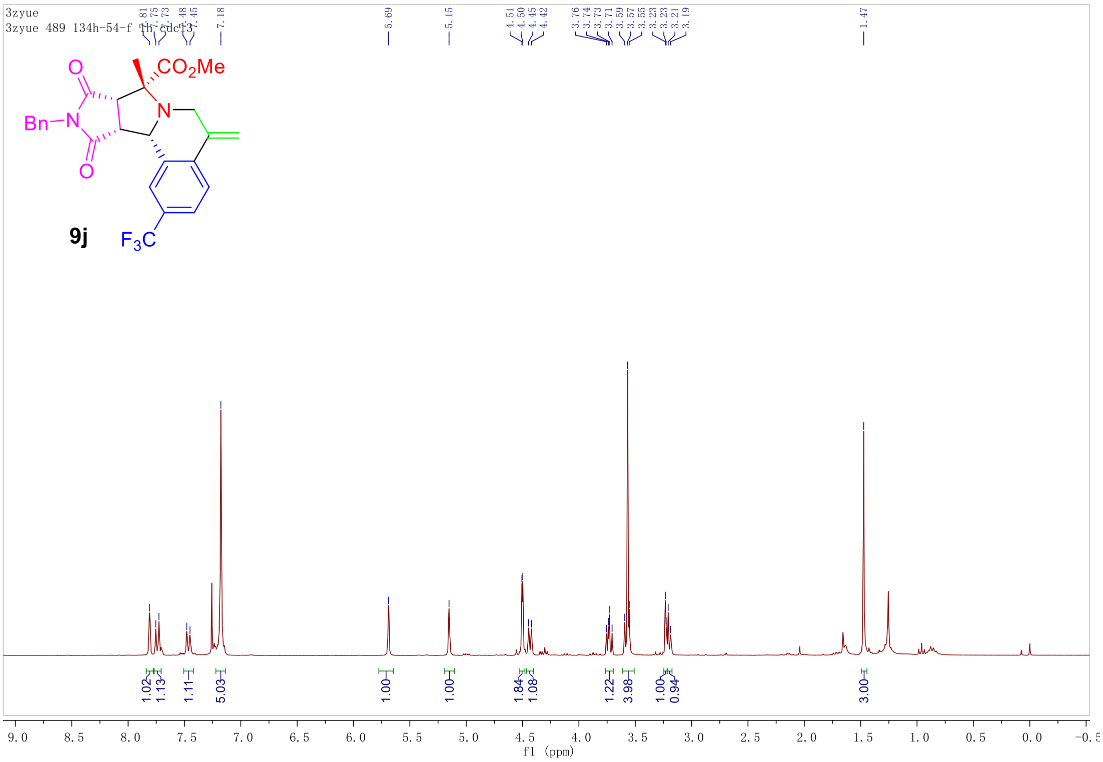
3zyue
3zyue 489 134h-54-f 1h cdcl3
7.81 7.75 7.73 7.48 7.45 7.18
5.69
5.15
4.51 4.50 4.45 4.42
3.76 3.74 3.73 3.71 3.59 3.57 3.55 3.23 3.23 3.21 3.19
1.47
CO2Me
Bn
N
O
F3C
9j
1.02 1.13 1.11 5.03 1.00 1.00 1.84 1.08 1.22 3.98 1.00 0.94 3.00
f1 (ppm)

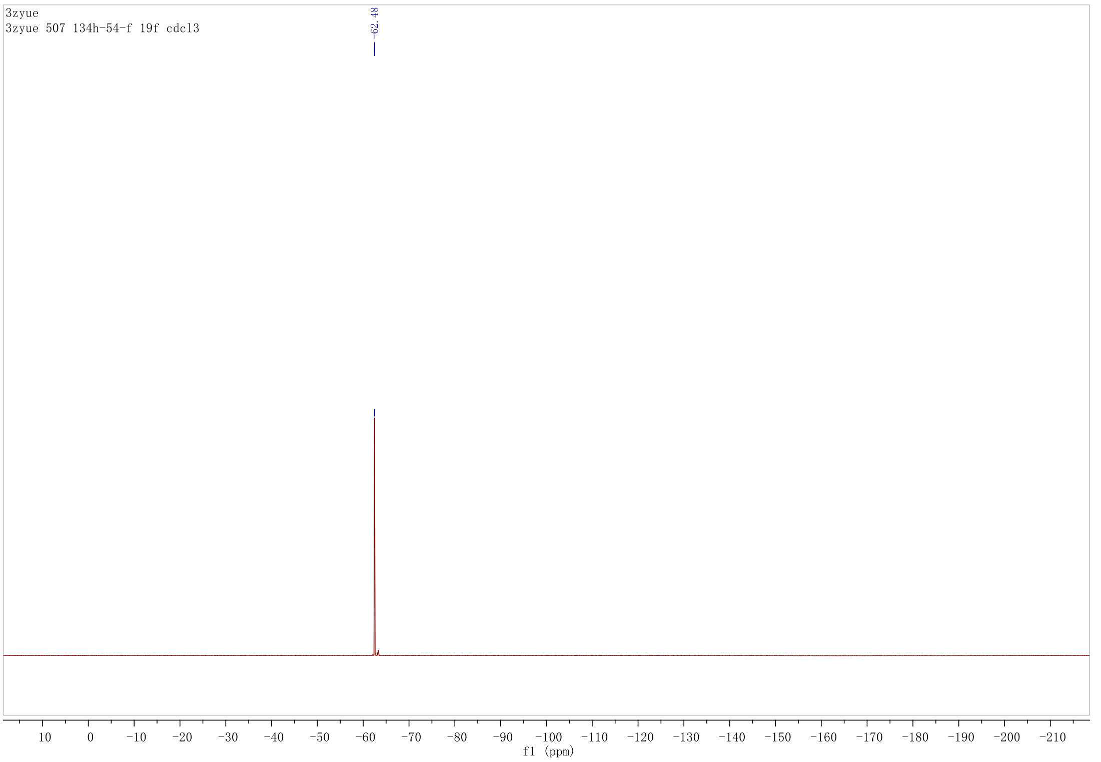
3zyue
3zyue 507 134h-54-f 19f cdcl3
-62.48
f1 (ppm)

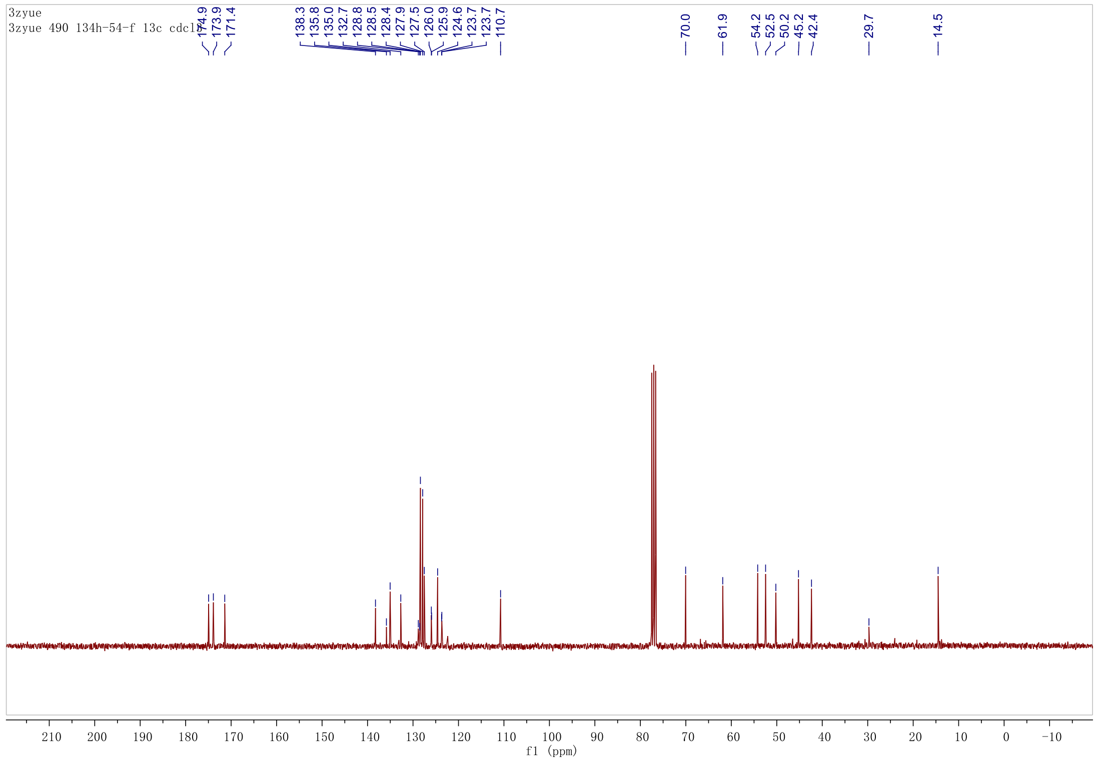
3zyue
3zyue 490 134h-54-f 13c cdcl3
174.9
173.9
171.4
138.3
135.8
135.0
132.7
128.8
128.5
128.4
127.9
127.5
126.0
125.9
124.6
123.7
123.7
110.7
70.0
61.9
54.2
52.5
50.2
45.2
42.4
29.7
14.5
210 200 190 180 170 160 150 140 130 120 110 100 90 80 70 60 50 40 30 20 10 0 -10
f1 (ppm)

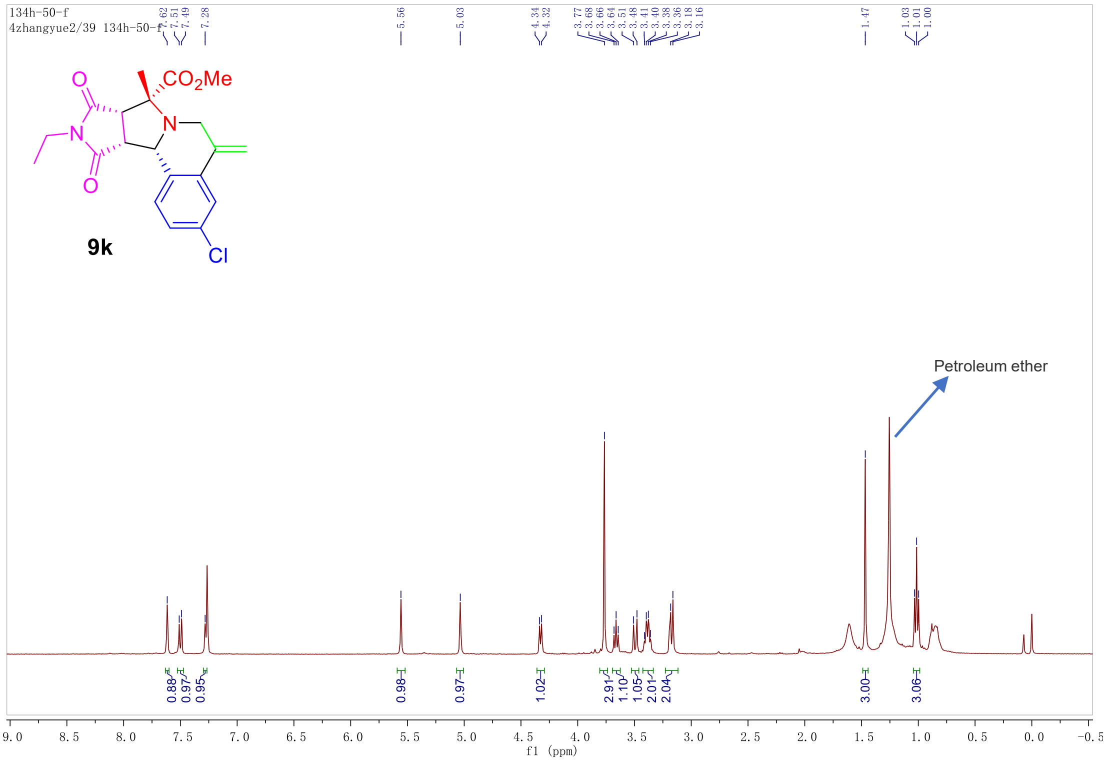
134h-50-f
4zhangyue2/39 134h-50-f
7.62
7.51
7.49
7.28
5.56
5.03
4.34
4.32
3.77
3.68
3.66
3.64
3.51
3.48
3.41
3.40
3.38
3.36
3.18
3.16
1.47
1.03
1.01
1.00
O
CO2Me
N
N
O
9k
Cl
Petroleum ether
0.88
0.97
0.95
0.98
0.97
1.02
2.91
1.10
1.05
2.01
2.04
3.00
3.06
f1 (ppm)

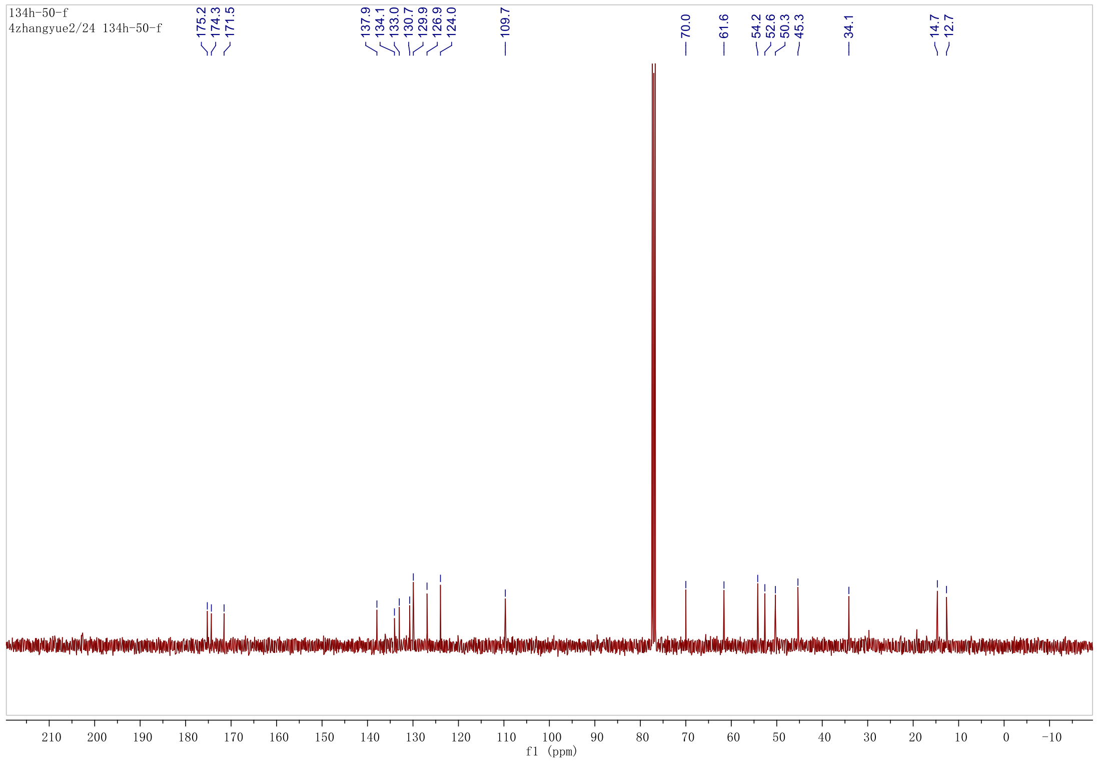
134h-50-f
4zhangyue2/24 134h-50-f
175.2
174.3
171.5
137.9
134.1
133.0
130.7
129.9
126.9
124.0
109.7
70.0
61.6
54.2
52.6
50.3
45.3
34.1
14.7
12.7
f1 (ppm)

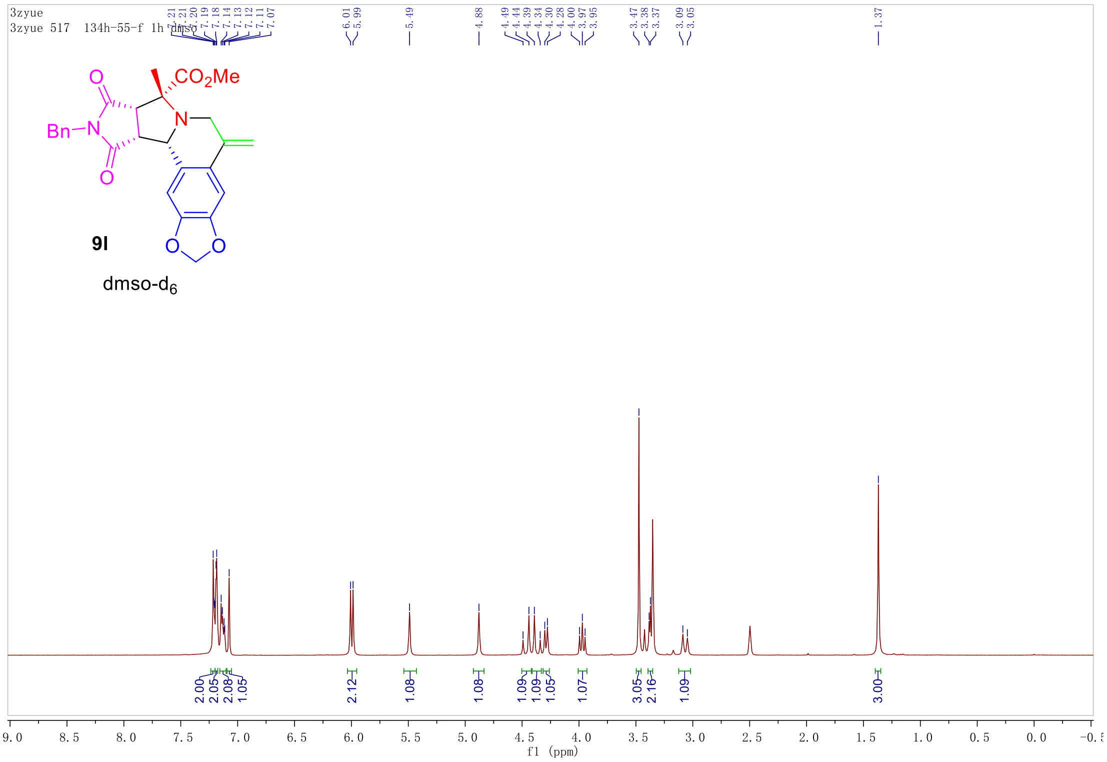
3zyue
3zyue 517 134h-55-f 1h dmso
CO2Me
Bn
N
O
O
O
9l
dmso-d6
f1 (ppm)

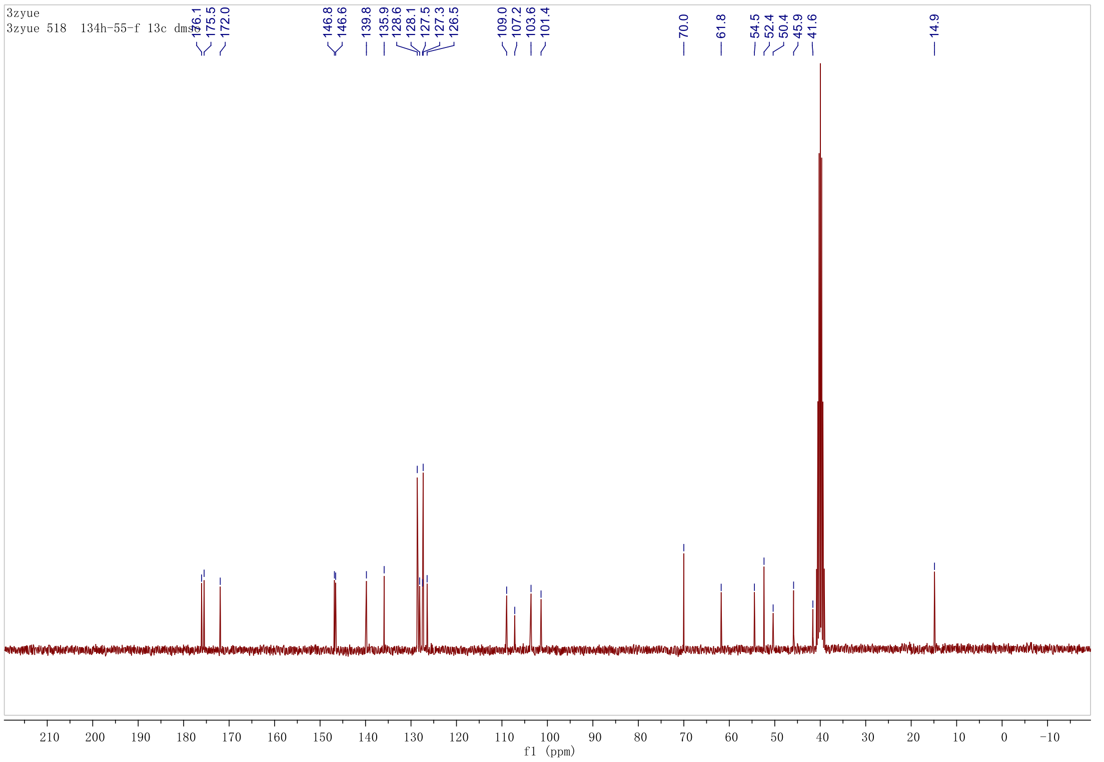
3zyue
3zyue 518 134h-55-f 13c dmso
176.1
175.5
172.0
146.8
146.6
139.8
135.9
128.6
128.1
127.5
127.3
126.5
109.0
107.2
103.6
101.4
70.0
61.8
54.5
52.4
50.4
45.9
41.6
14.9
f1 (ppm)

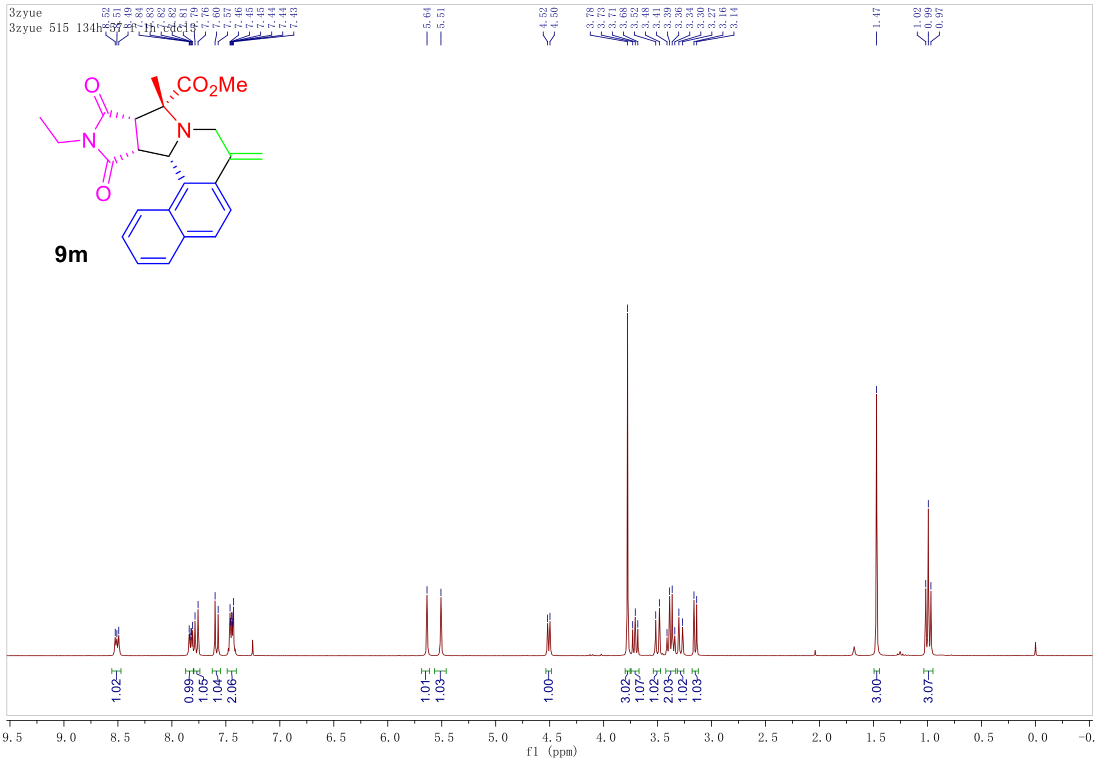
3zyue
9m
1.02
0.99
1.05
1.04
2.06
1.01
1.03
1.00
3.02
1.07
1.02
2.03
1.02
1.03
3.00
3.07
f1 (ppm)

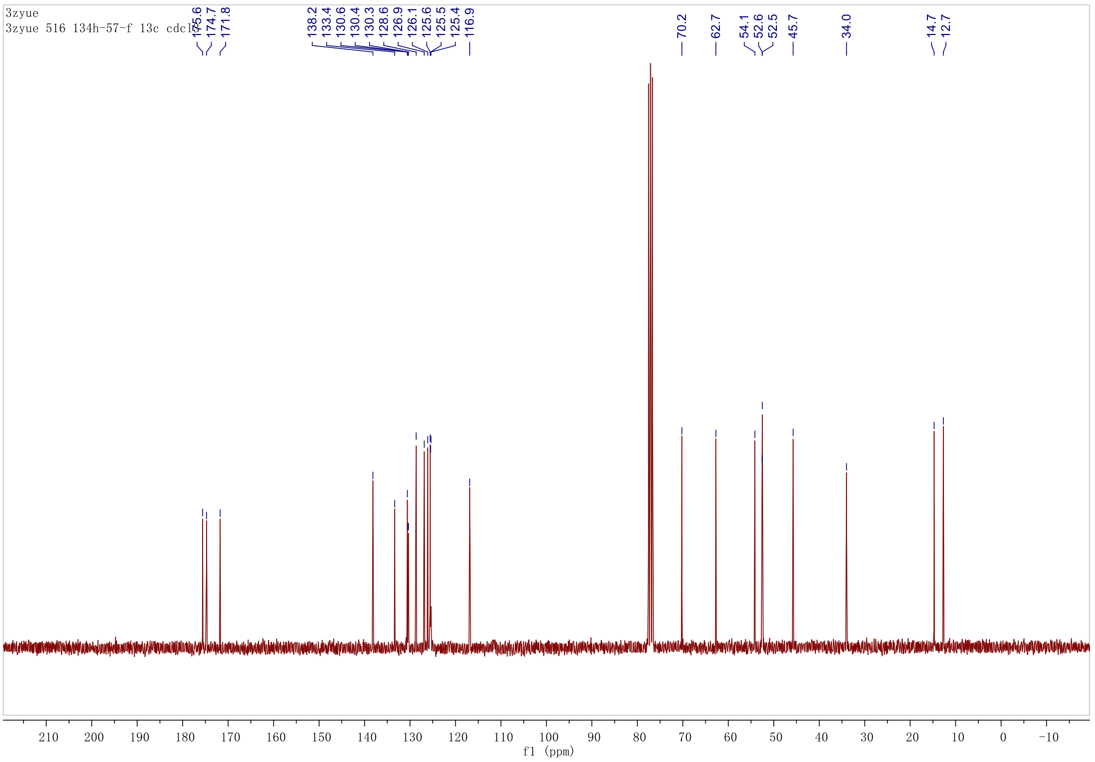
3zyue
3zyue 516 134h-57-f 13c cdcl3
175.6
174.7
171.8
138.2
133.4
130.6
130.4
130.3
128.6
126.9
126.1
125.6
125.5
125.4
116.9
70.2
62.7
54.1
52.6
52.5
45.7
34.0
14.7
12.7
f1 (ppm)

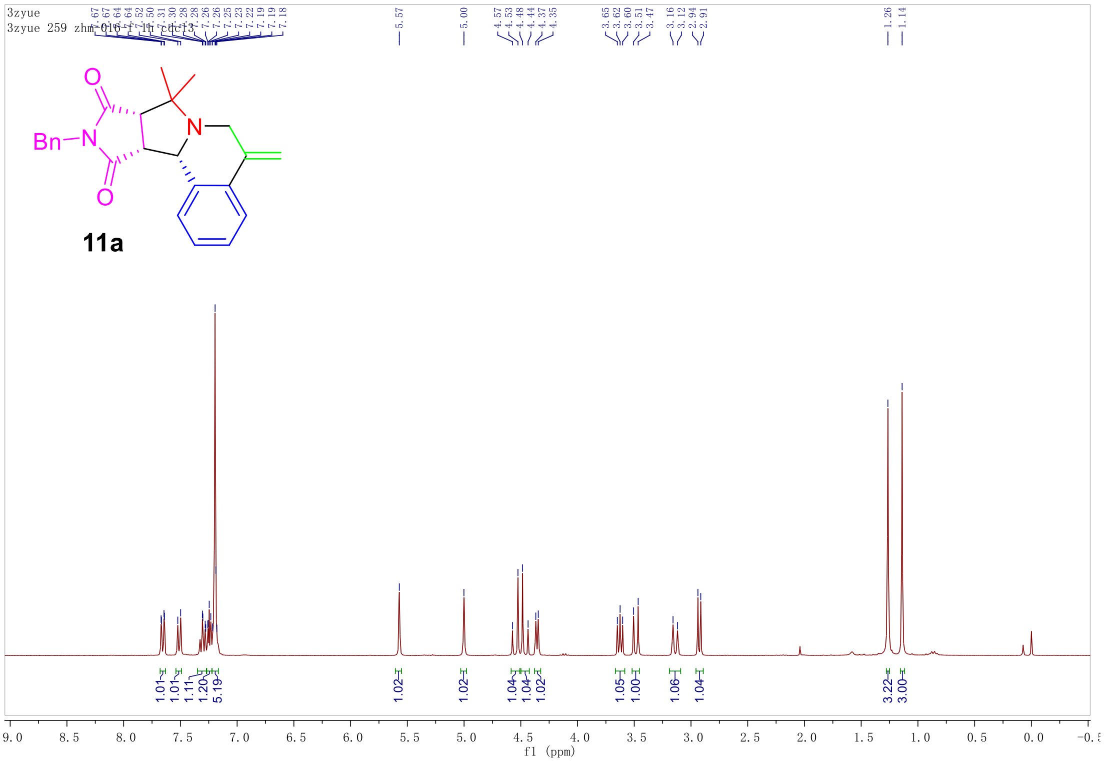
3zyue
7.67 7.67 7.64 7.64 7.52 7.50 7.31 7.30 7.28 7.28 7.26 7.26 7.25 7.23 7.22 7.19 7.19 7.18
5.57
5.00
4.57 4.53 4.48 4.44 4.37 4.35
3.65 3.62 3.60 3.51 3.47
3.16 3.12 2.94 2.91
1.26 1.14
Bn
N
O
O
N
11a
1.01 1.01 1.11 1.20 5.19
1.02
1.02
1.04 1.04 1.02
1.05 1.00
1.06 1.04
3.22 3.00
9.0 8.5 8.0 7.5 7.0 6.5 6.0 5.5 5.0 4.5 4.0 3.5 3.0 2.5 2.0 1.5 1.0 0.5 0.0
f1 (ppm)

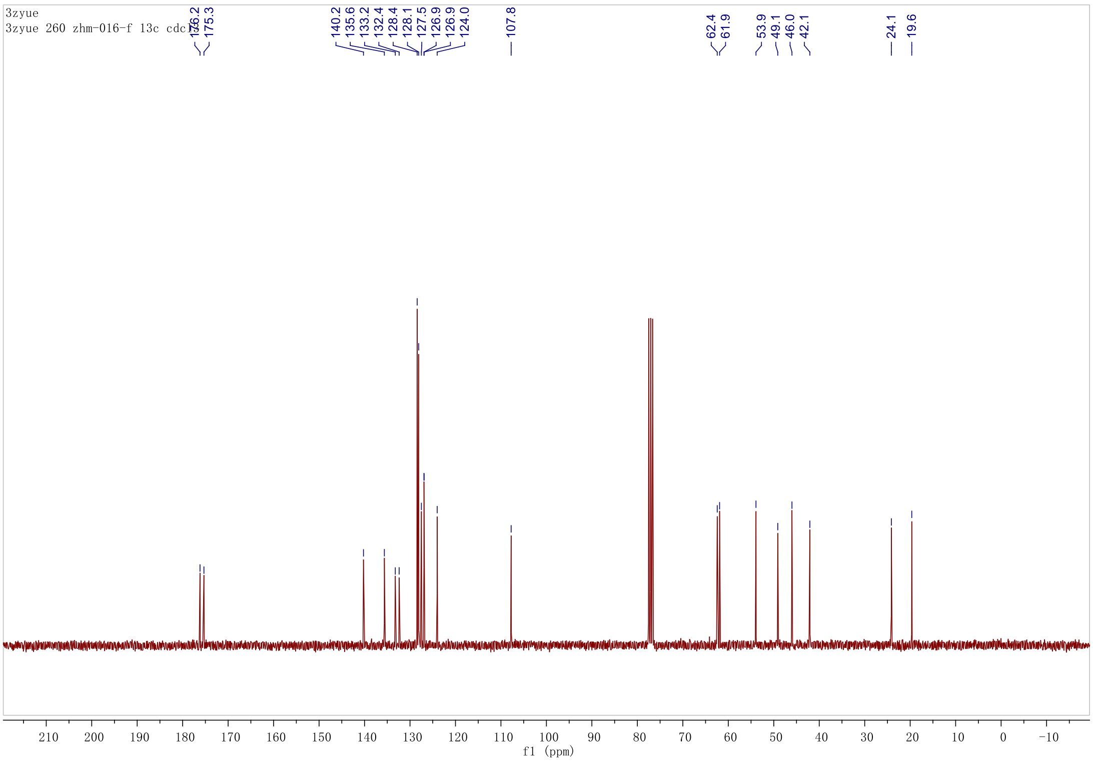
3zyue
3zyue 260 zhm-016-f 13c cdcl3
176.2 175.3
140.2 135.6 133.2 132.4 128.4 128.1 127.5 126.9 126.9 124.0
107.8
62.4 61.9
53.9 49.1 46.0 42.1
24.1 19.6
210 200 190 180 170 160 150 140 130 120 110 100 90 80 70 60 50 40 30 20 10 0 -10
f1 (ppm)

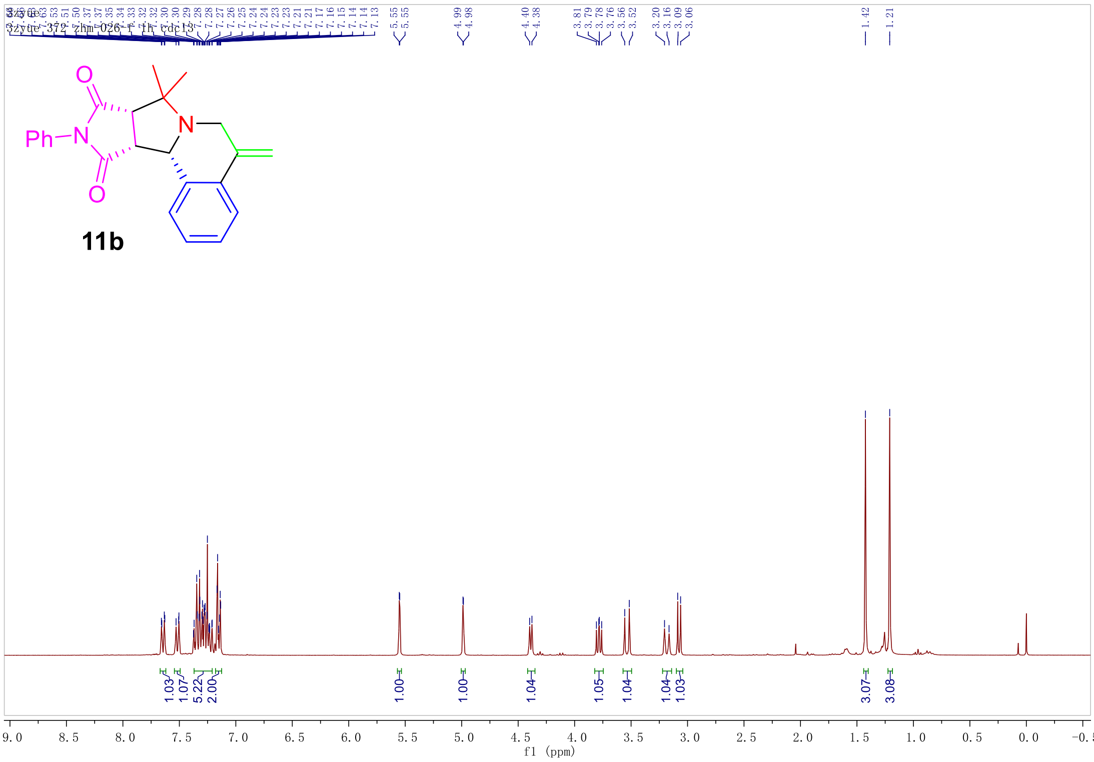
3zyue 372 zhm-026-f 1h cdcl3
Ph
N
O
11b
5.55
4.99
4.98
4.40
4.38
3.81
3.79
3.78
3.76
3.56
3.52
3.20
3.16
3.09
3.06
1.42
1.21
1.03
1.07
5.22
2.00
1.00
1.00
1.04
1.05
1.04
1.04
1.03
3.07
3.08
f1 (ppm)

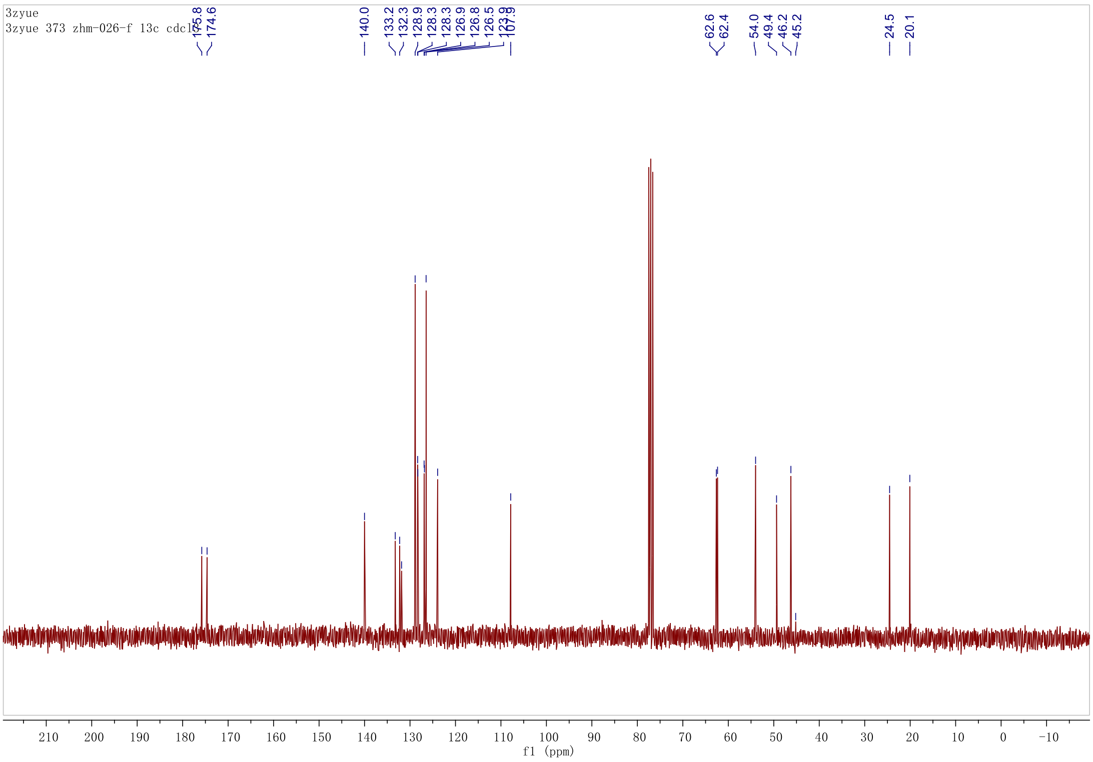
3zyue
3zyue 373 zhm-026-f 13c cdcl3
175.8
174.6
140.0
133.2
132.3
128.9
128.3
128.3
126.9
126.8
126.5
123.9
107.9
62.6
62.4
54.0
49.4
46.2
45.2
24.5
20.1
f1 (ppm)

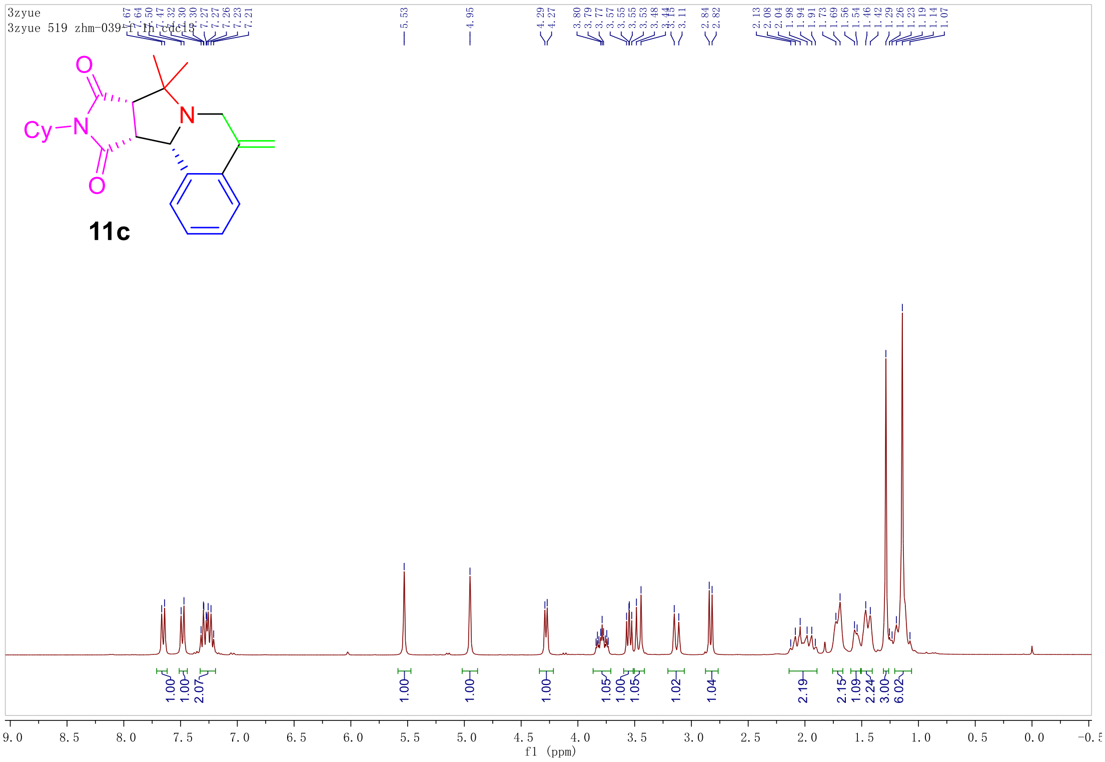
3zyue
11c
Cy
f1 (ppm)
9.0 8.5 8.0 7.5 7.0 6.5 6.0 5.5 5.0 4.5 4.0 3.5 3.0 2.5 2.0 1.5 1.0 0.5 0.0
1.00 1.00 2.07 1.00 1.00 1.00 1.05 1.00 1.05 1.02 1.04 2.19 2.15 1.09 2.24 3.00 6.02

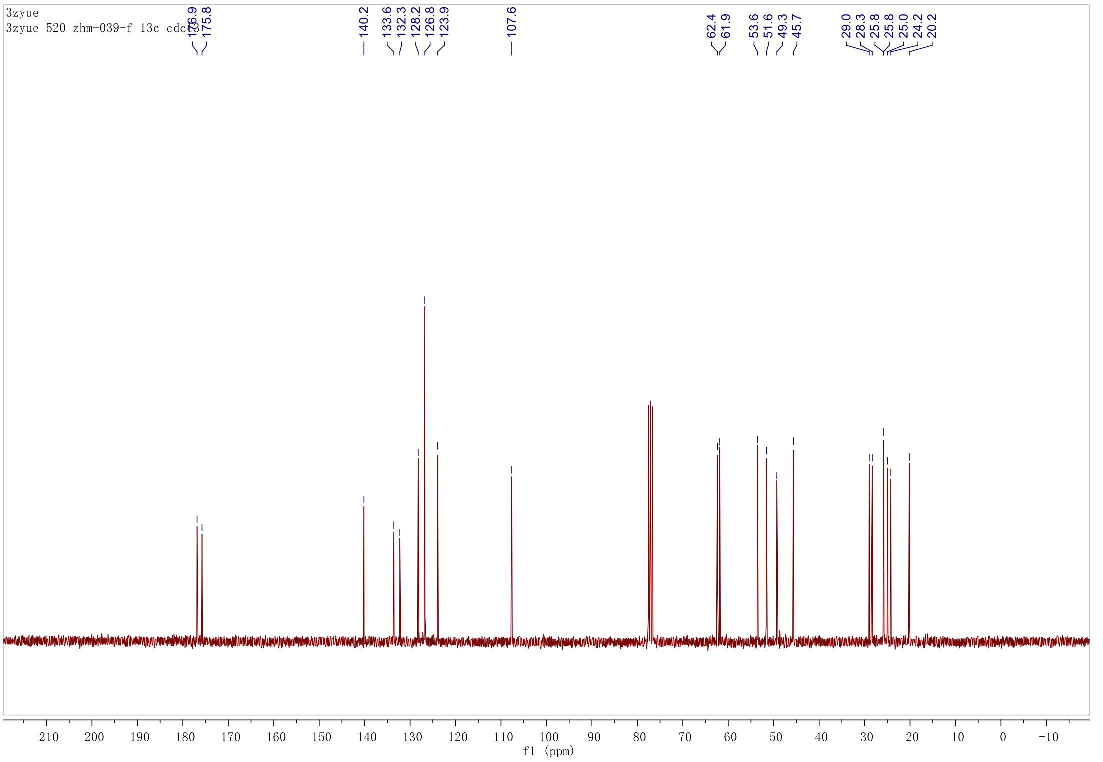
3zyue
3zyue 520 zhm-039-f 13c cdcl3
176.9 175.8 140.2 133.6 132.3 128.2 126.8 123.9 107.6 62.4 61.9 53.6 51.6 49.3 45.7 29.0 28.3 25.8 25.8 25.0 24.2 20.2
210 200 190 180 170 160 150 140 130 120 110 100 90 80 70 60 50 40 30 20 10 0 -10
f1 (ppm)

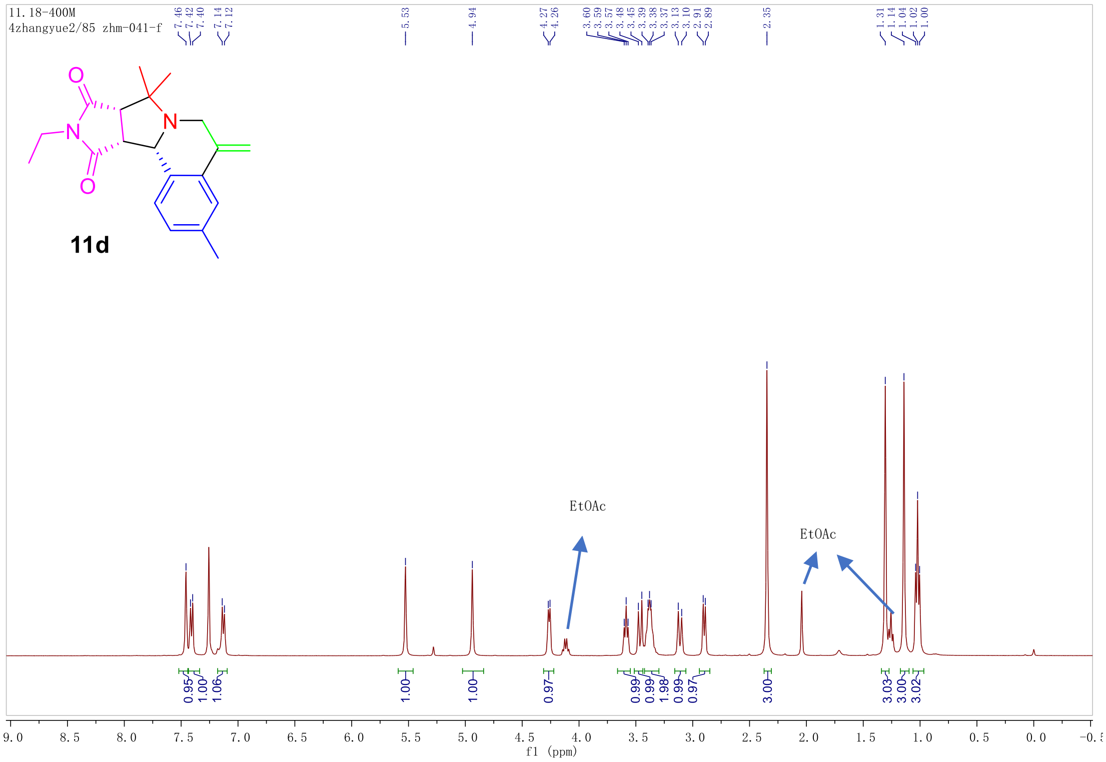
11.18-400M
4zhangyue2/85 zhm-041-f
11d
EtOAc
EtOAc
f1 (ppm)

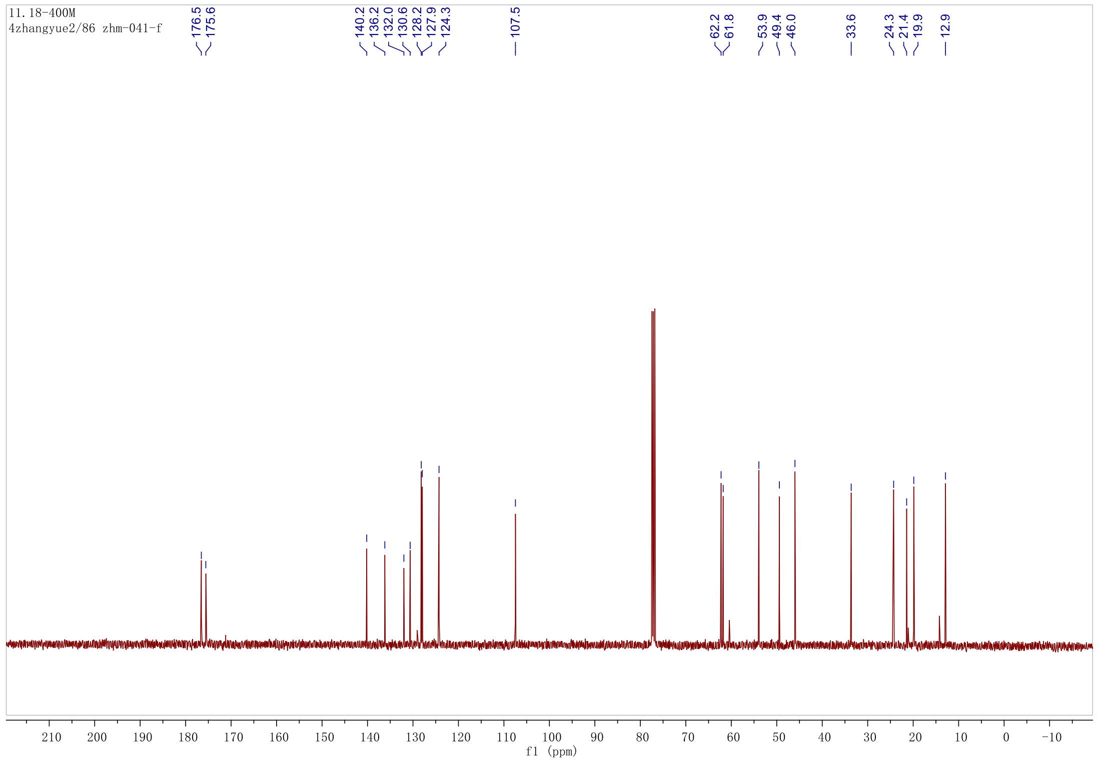
11.18-400M
4zhangyue2/86 zhm-041-f
f1 (ppm)

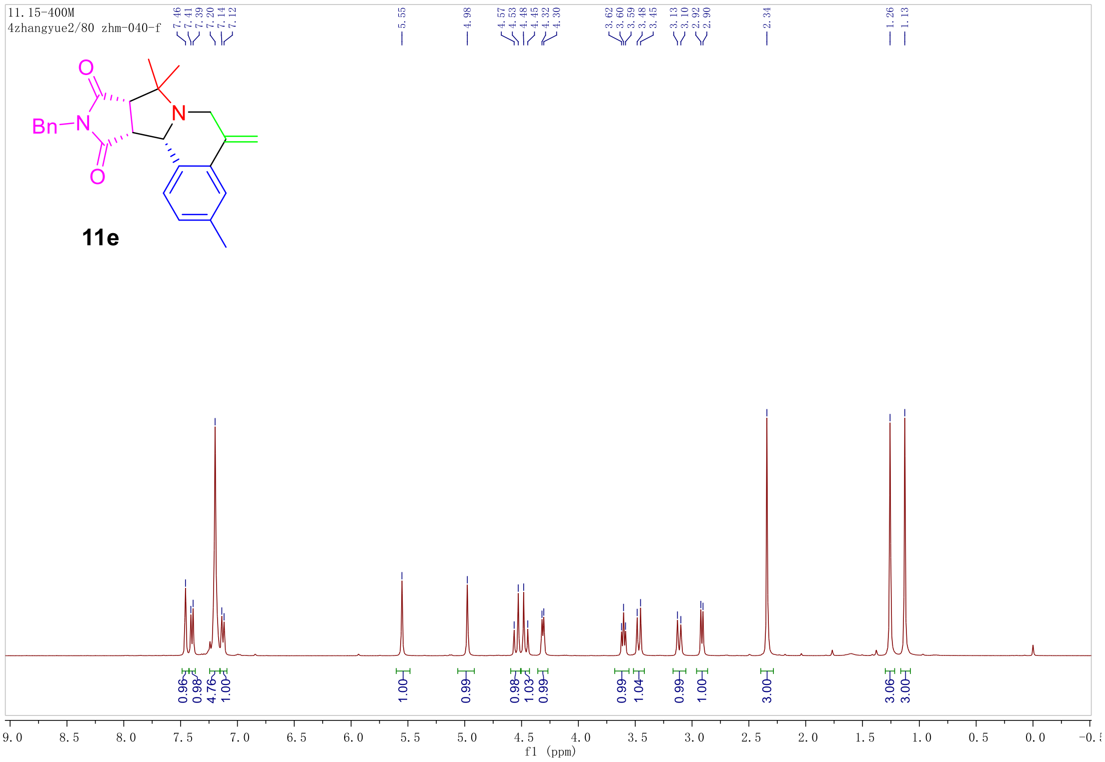
11.15-400M
4zhangyue2/80 zhm-040-f
7.46 7.41 7.39 7.20 7.14 7.12
5.55
4.98
4.57 4.53 4.48 4.45 4.32 4.30
3.62 3.60 3.59 3.48 3.45
3.13 3.10 2.92 2.90
2.34
1.26 1.13
Bn
N
O
O
N
11e
0.96 0.98 4.76 1.00
1.00
0.99
0.98 1.03 0.99
0.99 1.04
0.99 1.00
3.00
3.06 3.00
f1 (ppm)

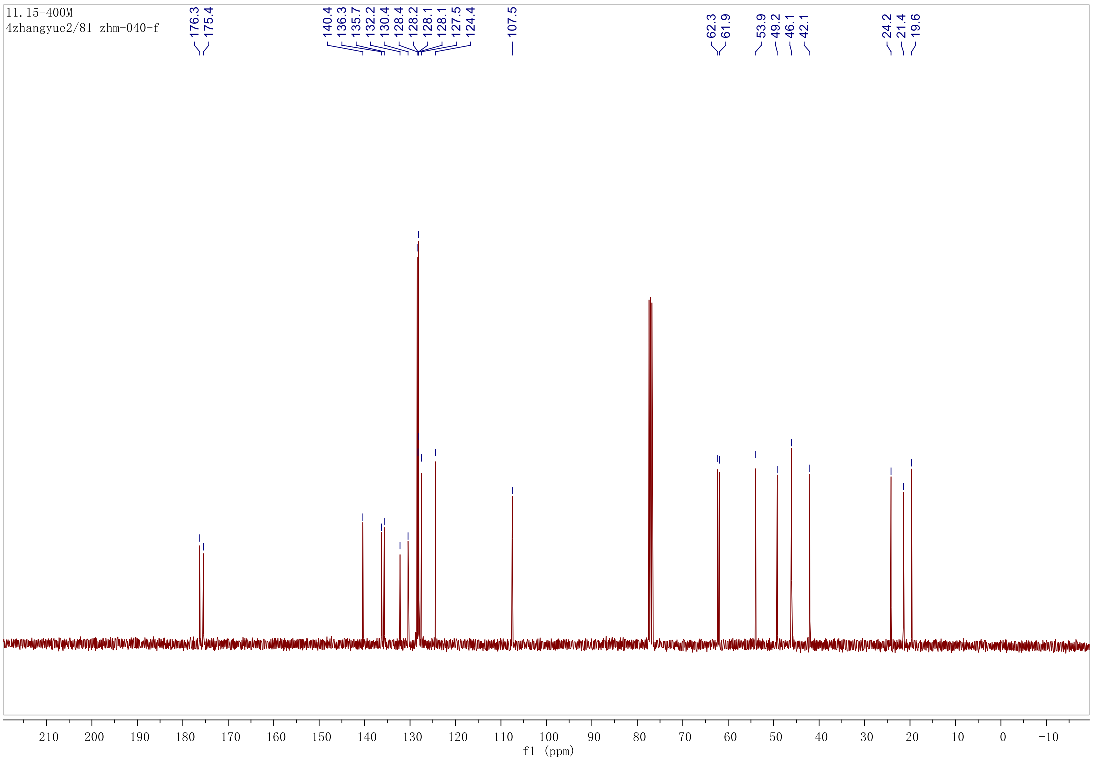
11.15-400M
4zhangyue2/81 zhm-040-f
176.3 175.4
140.4 136.3 135.7 132.2 130.4 128.4 128.2 128.1 127.5 124.4
107.5
62.3 61.9
53.9 49.2 46.1 42.1
24.2 21.4 19.6
f1 (ppm)

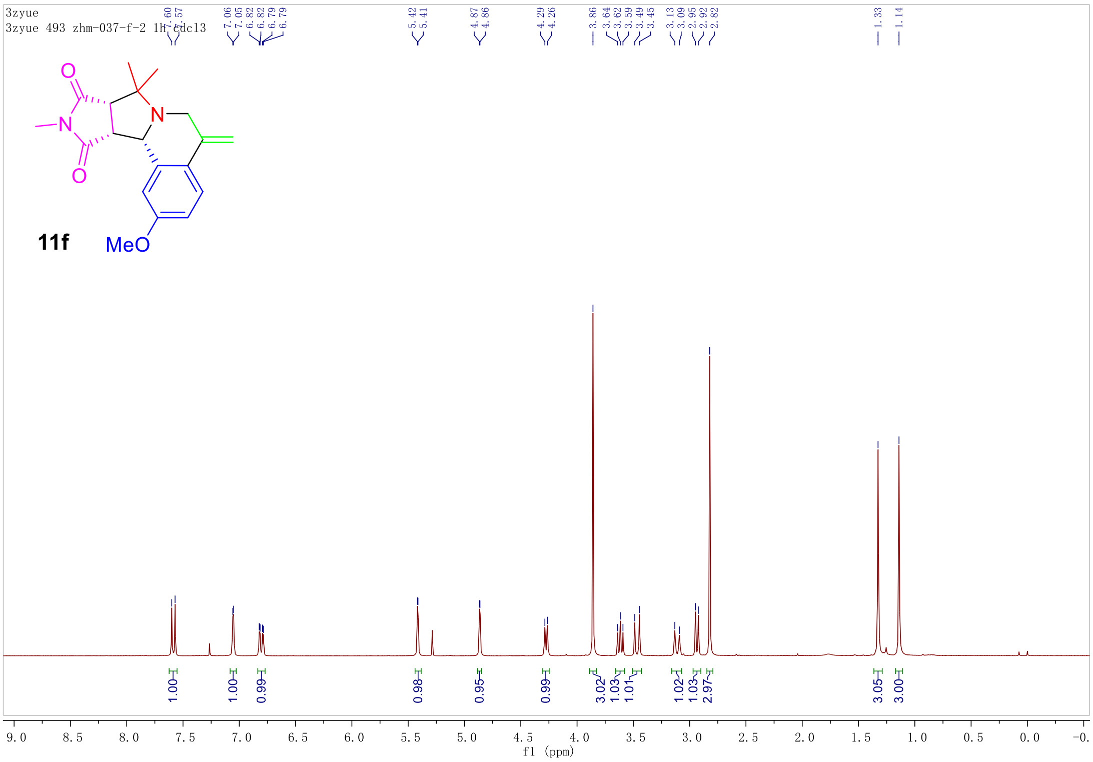
3zyue
3zyue 493 zhm-037-f-2 1h cdcl3
7.60
7.57
7.06
7.05
6.82
6.82
6.79
6.79
5.42
5.41
4.87
4.86
4.29
4.26
3.86
3.64
3.62
3.59
3.49
3.45
3.13
3.09
2.95
2.92
2.82
1.33
1.14
11f
MeO
1.00
1.00
0.99
0.98
0.95
0.99
3.02
1.03
1.01
1.02
1.03
2.97
3.05
3.00
f1 (ppm)

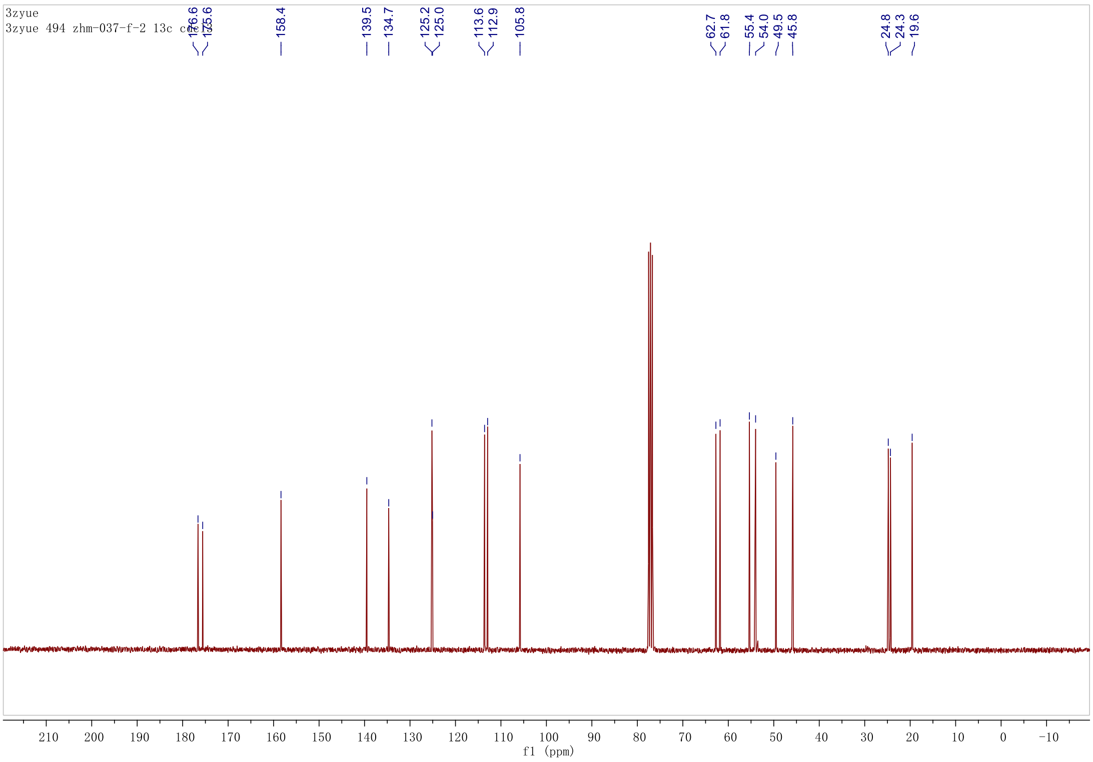
3zyue
3zyue 494 zhm-037-f-2 13c cdcl3
176.6
175.6
158.4
139.5
134.7
125.2
125.0
113.6
112.9
105.8
62.7
61.8
55.4
54.0
49.5
45.8
24.8
24.3
19.6
f1 (ppm)

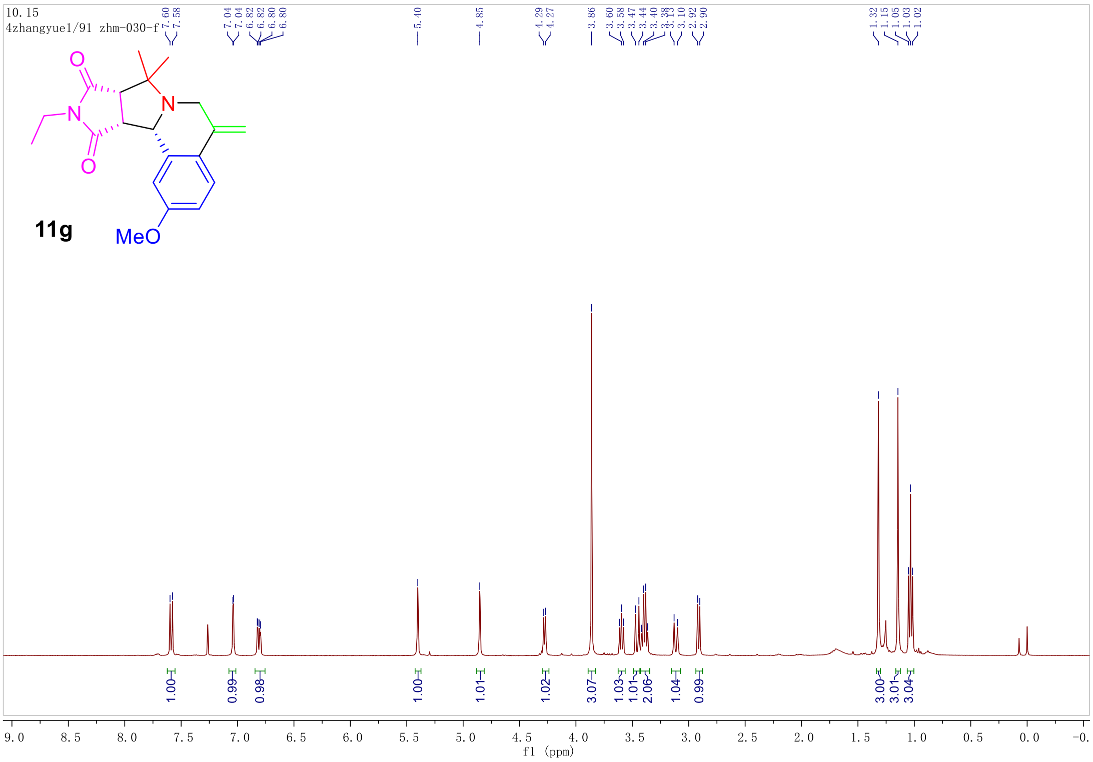
10.15
4zhangyue1/91 zhm-030-f
7.60
7.58
7.04
7.04
6.82
6.82
6.80
6.80
5.40
4.85
4.29
4.27
3.86
3.60
3.58
3.47
3.44
3.40
3.38
3.13
3.10
2.92
2.90
1.32
1.15
1.05
1.03
1.02
11g
MeO
1.00
0.99
0.98
1.00
1.01
1.02
3.07
1.03
1.01
2.06
1.04
0.99
3.00
3.01
3.04
9.0
8.5
8.0
7.5
7.0
6.5
6.0
5.5
5.0
4.5
4.0
3.5
3.0
2.5
2.0
1.5
1.0
0.5
0.0
-0.5
f1 (ppm)

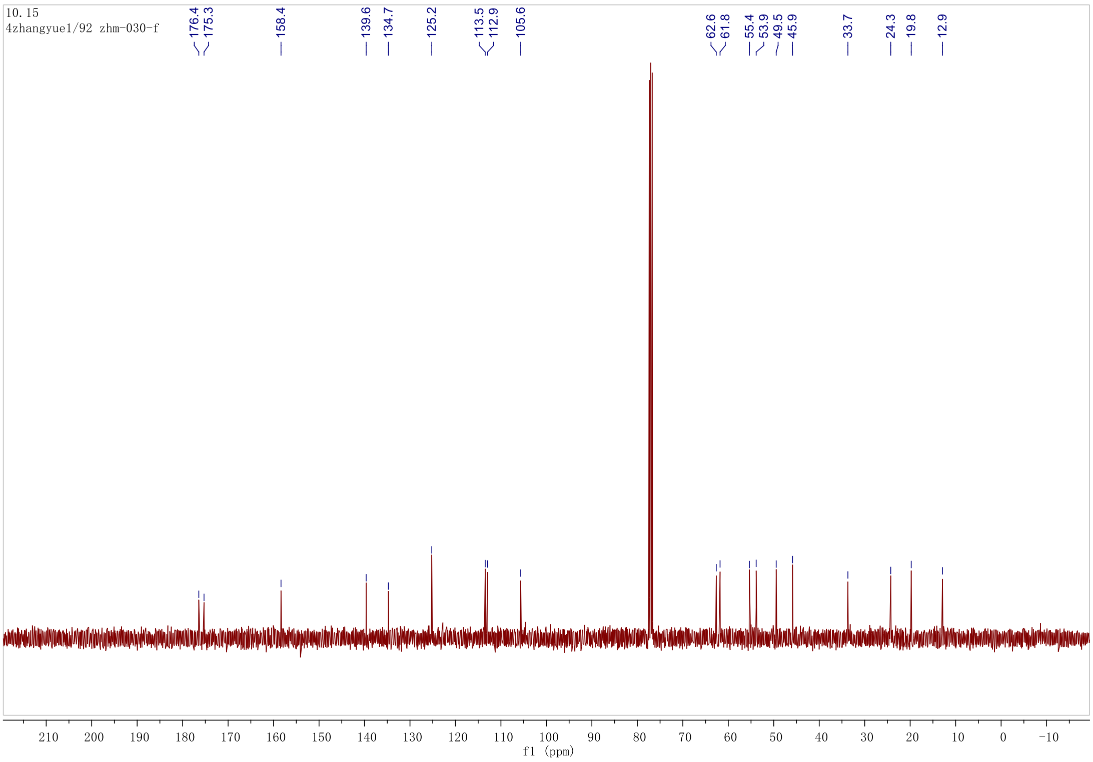
10.15
4zhangyue1/92 zhm-030-f
176.4
175.3
158.4
139.6
134.7
125.2
113.5
112.9
105.6
62.6
61.8
55.4
53.9
49.5
45.9
33.7
24.3
19.8
12.9
210
200
190
180
170
160
150
140
130
120
110
100
90
80
70
60
50
40
30
20
10
0
-10
f1 (ppm)

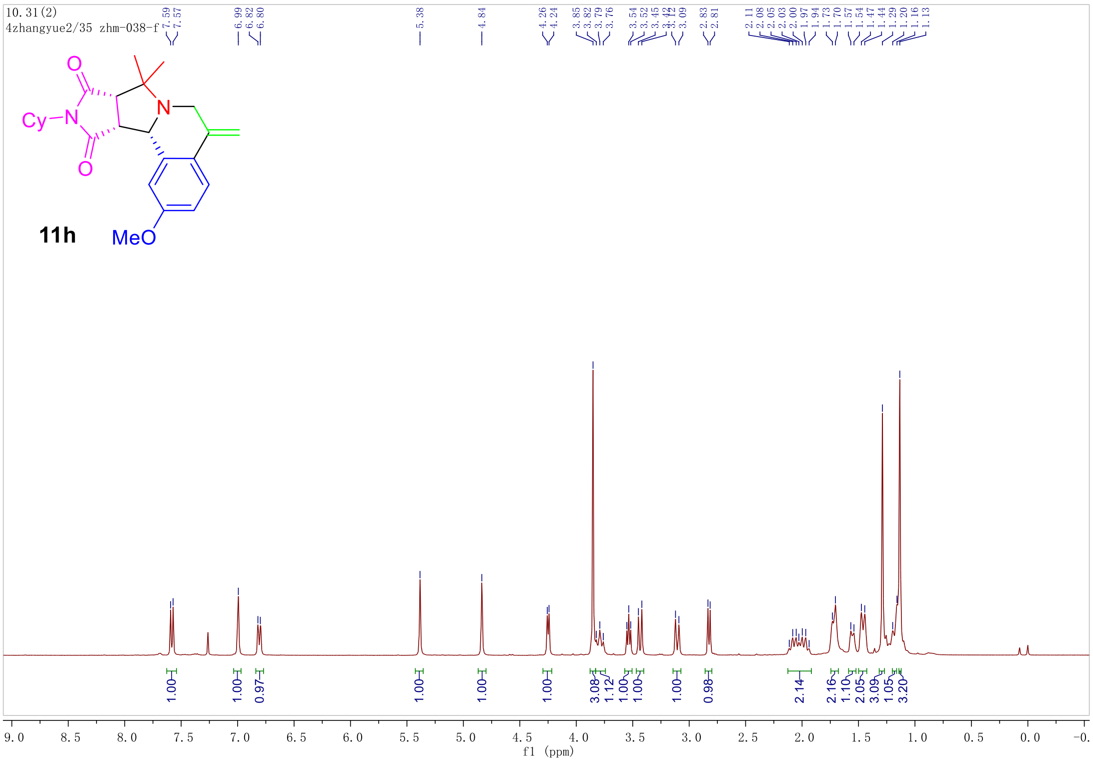
10.31(2)
4zhangyue2/35 zhm-038-f
11h
MeO
Cy
N
O
f1 (ppm)

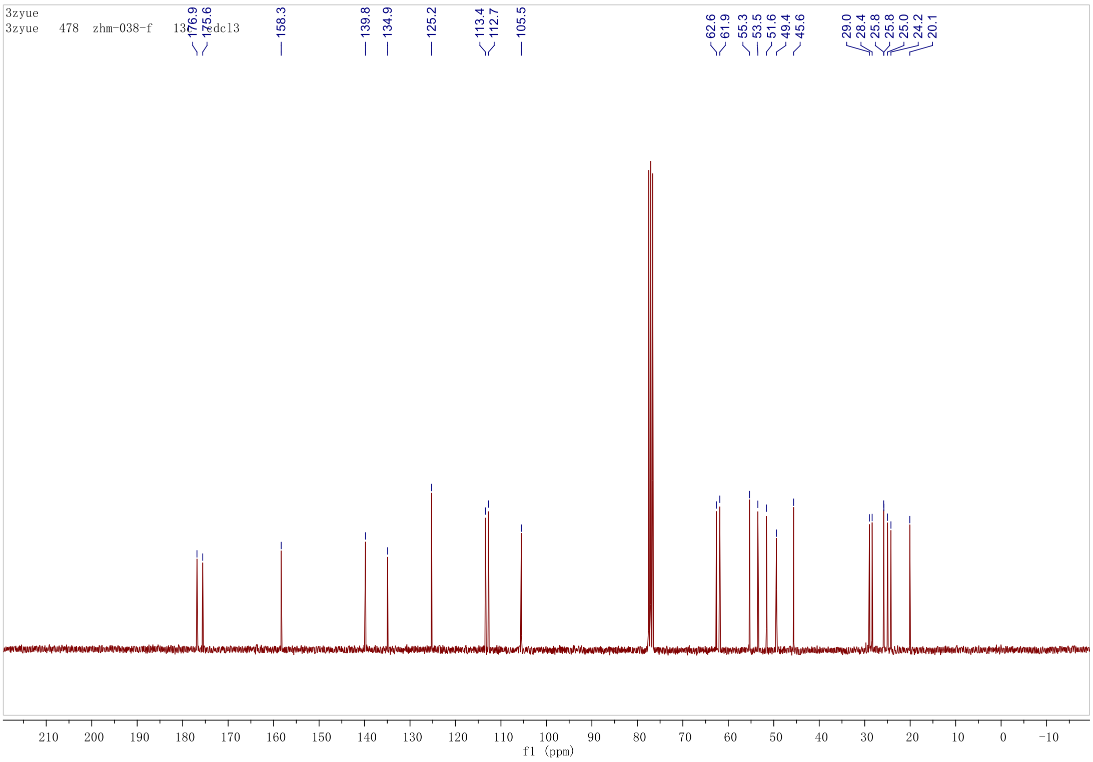
3zyue
3zyue 478 zhm-038-f 13C cdcl3
176.9
175.6
158.3
139.8
134.9
125.2
113.4
112.7
105.5
62.6
61.9
55.3
53.5
51.6
49.4
45.6
29.0
28.4
25.8
25.8
25.0
24.2
20.1
f1 (ppm)

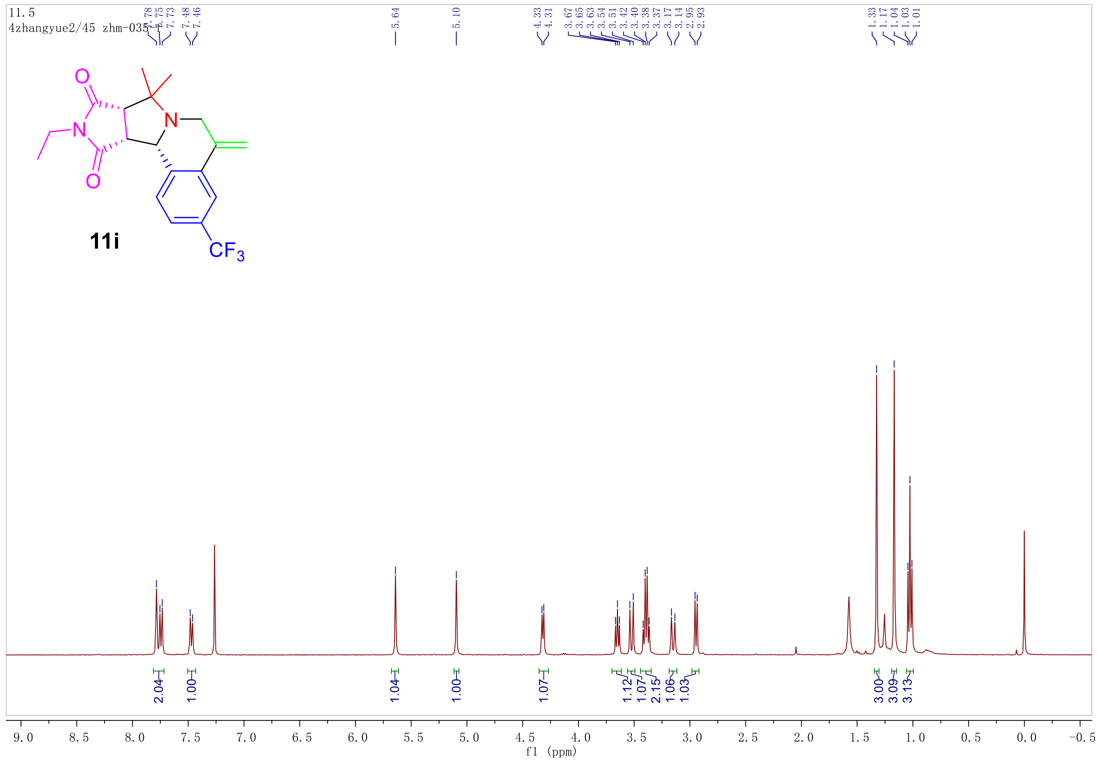
11.5
4zhangyue2/45 zhm-035
11i
CF3
f1 (ppm)

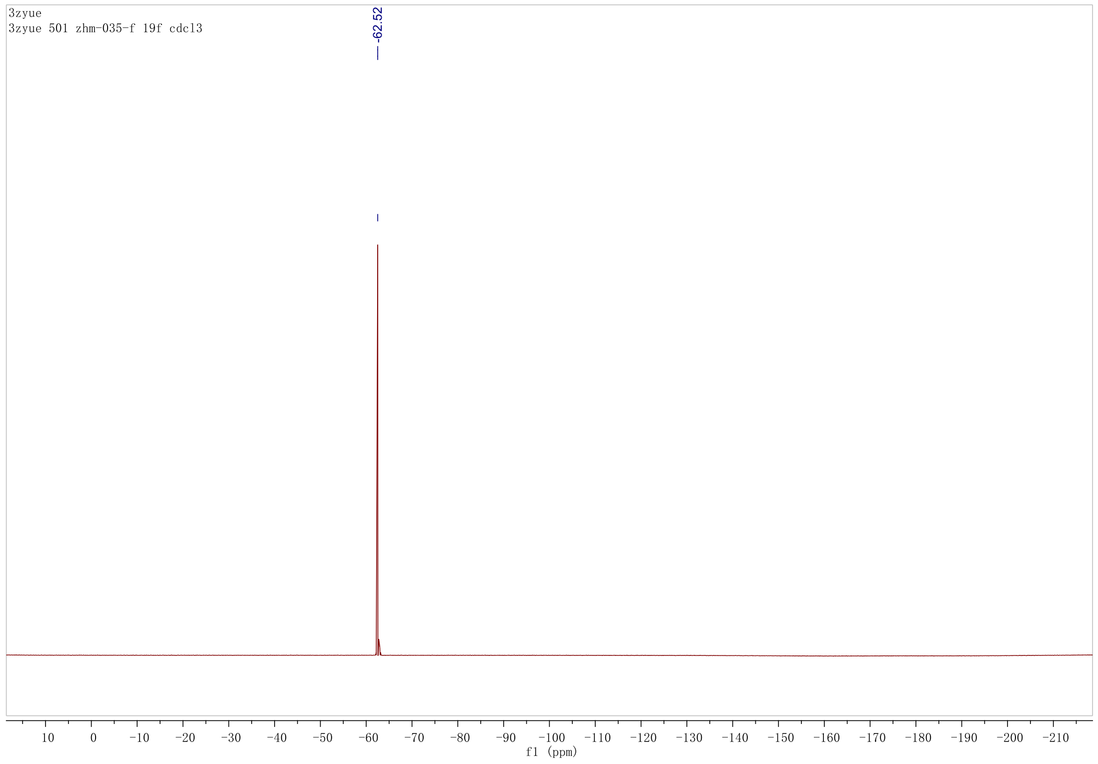
3zyue
3zyue 501 zhm-035-f 19f cdcl3
-62.52
f1 (ppm)

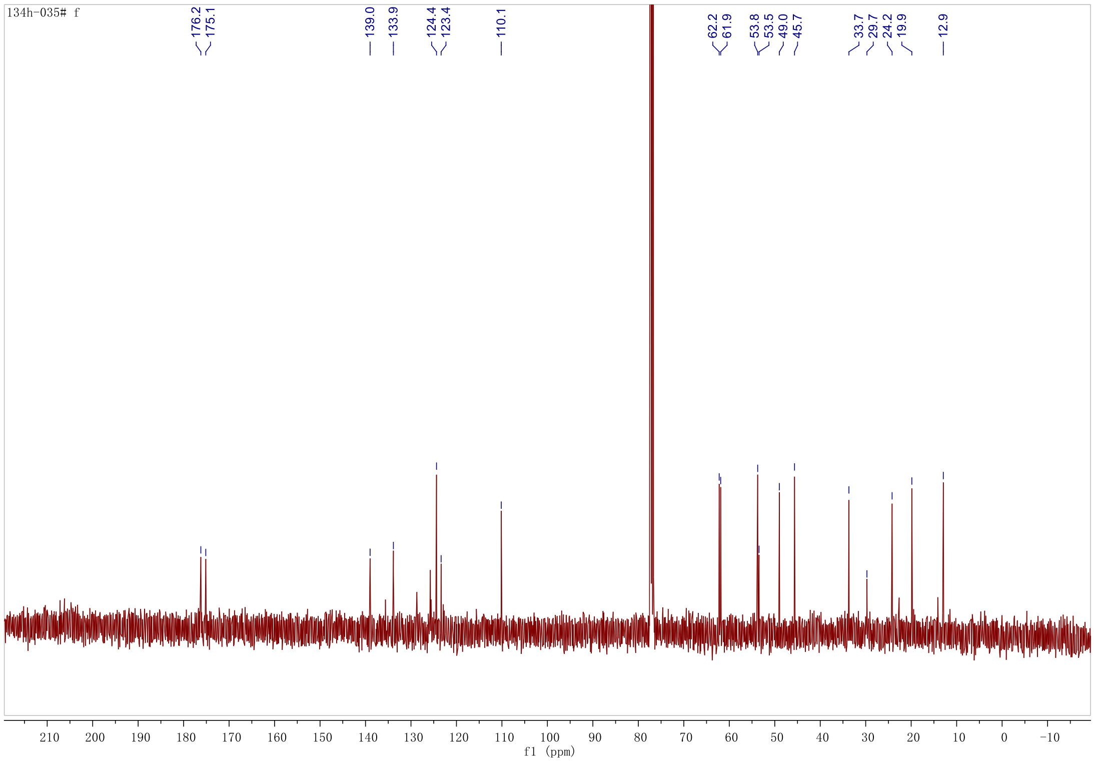
134h-035# f
176.2
175.1
139.0
133.9
124.4
123.4
110.1
62.2
61.9
53.8
53.5
49.0
45.7
33.7
29.7
24.2
19.9
12.9
210
200
190
180
170
160
150
140
130
120
110
100
90
80
70
60
50
40
30
20
10
0
-10
f1 (ppm)

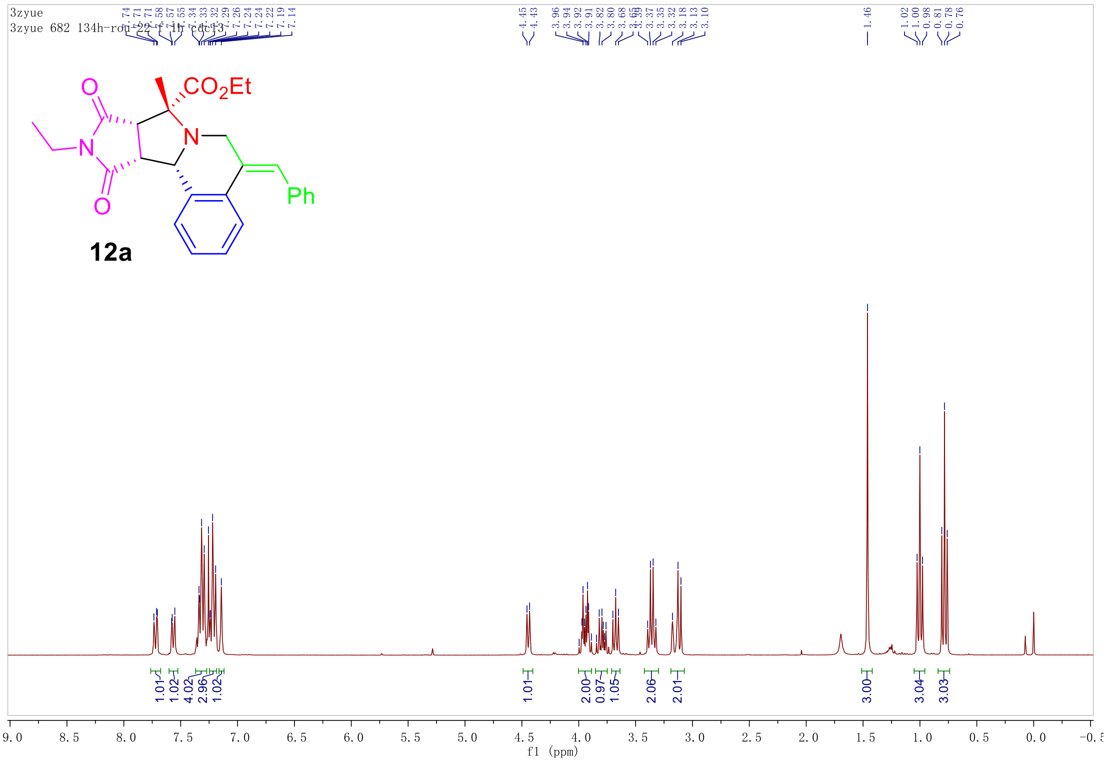

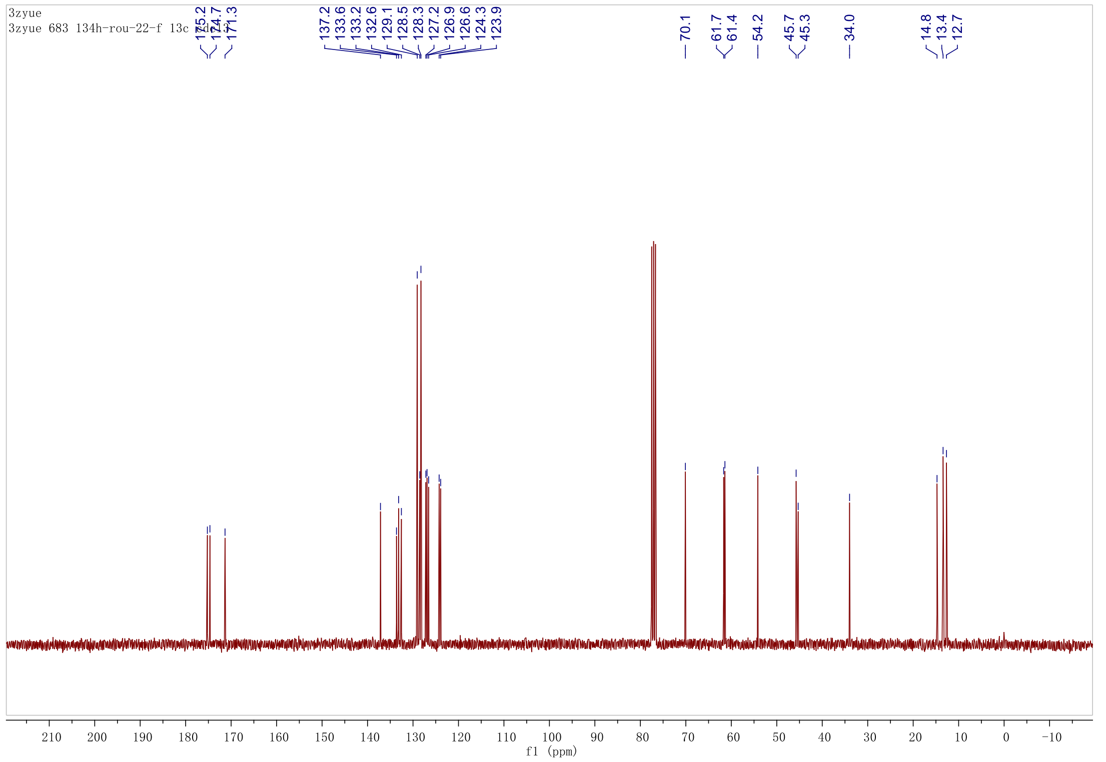

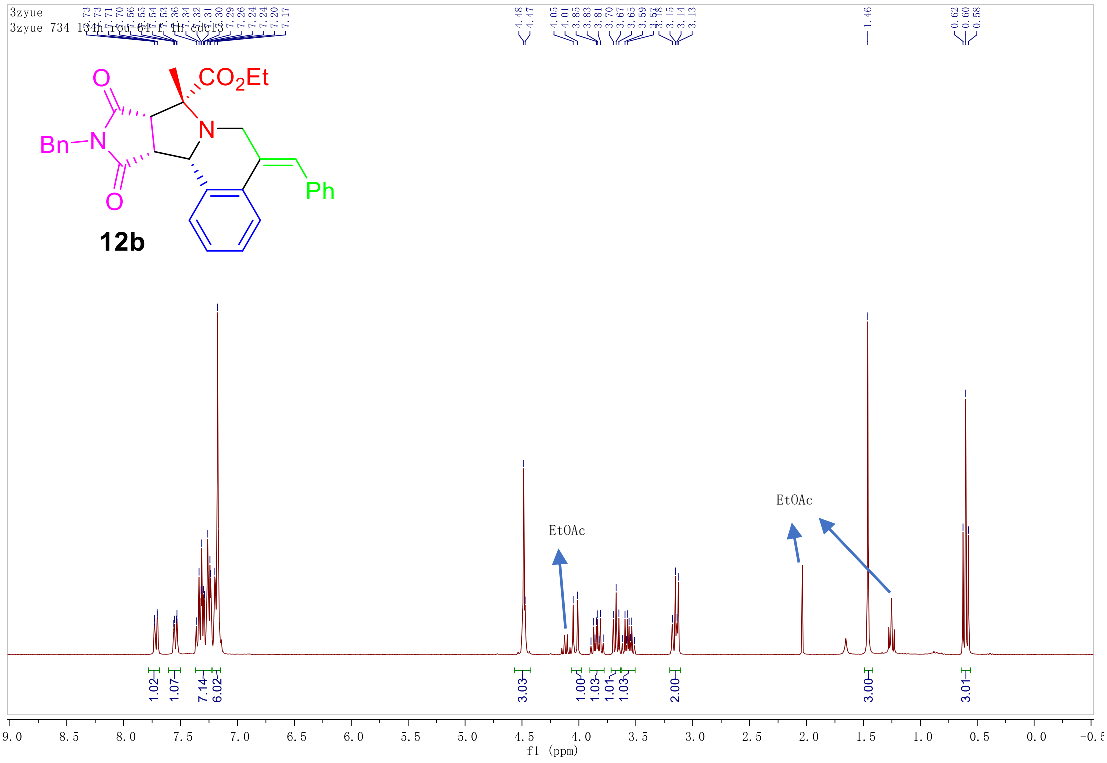
3zyue
12b
CO2Et
Bn
N
O
Ph
EtOAc
EtOAc
f1 (ppm)

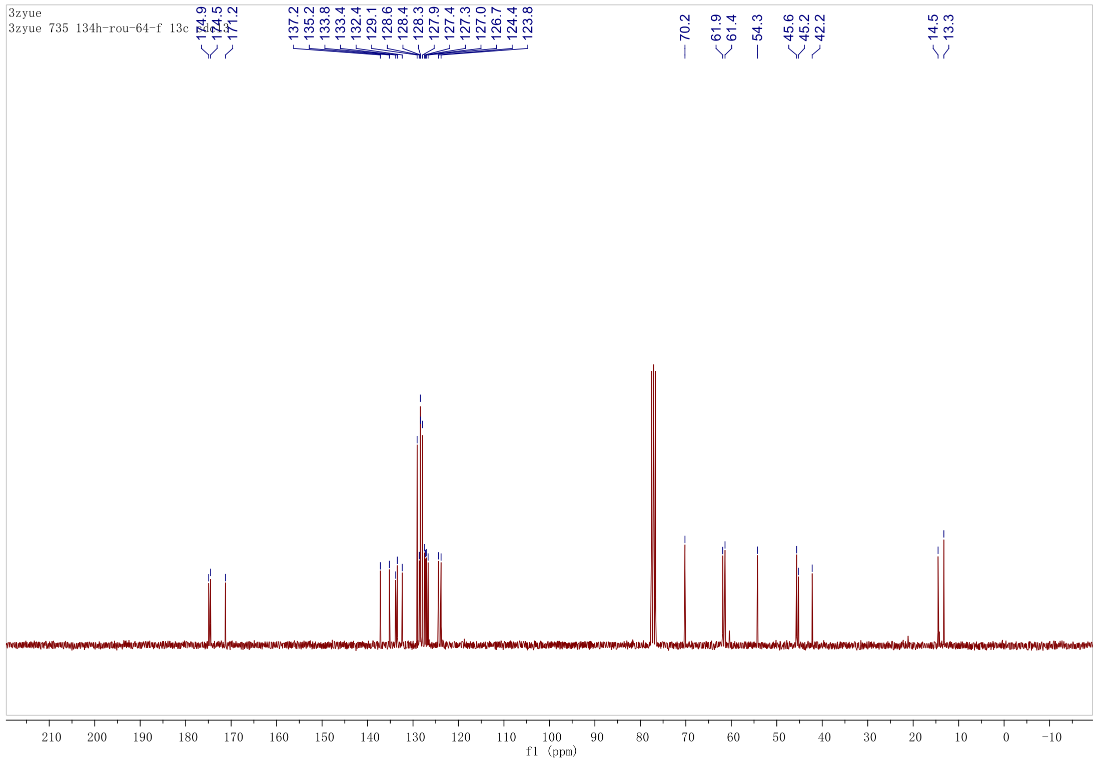
3zyue
3zyue 735 134h-rou-64-f 13c
174.9
174.5
171.2
137.2
135.2
133.8
133.4
132.4
129.1
128.6
128.4
128.3
127.9
127.4
127.3
127.0
126.7
124.4
123.8
70.2
61.9
61.4
54.3
45.6
45.2
42.2
14.5
13.3
f1 (ppm)

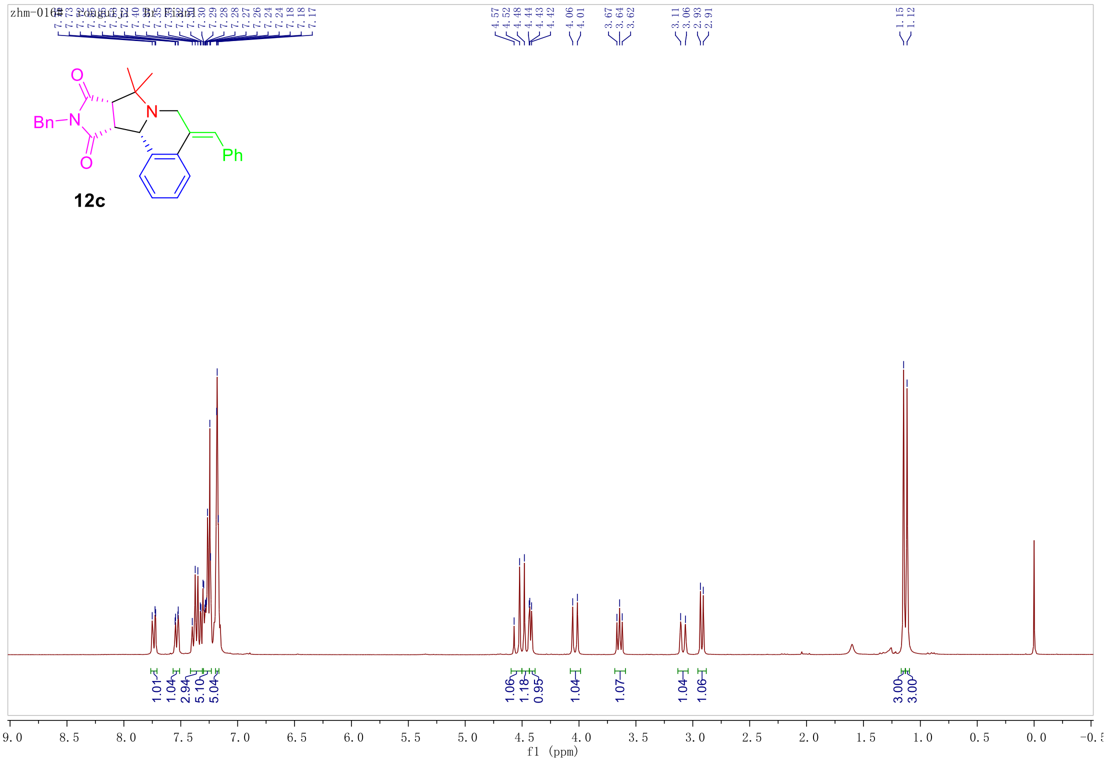
zhm-016#
12c
Bn
O
N
Ph
4.57
4.52
4.48
4.44
4.43
4.42
4.06
4.01
3.67
3.64
3.62
3.11
3.06
2.93
2.91
1.15
1.12
1.01
1.04
2.94
5.10
5.04
1.06
1.18
0.95
1.04
1.07
1.04
1.06
3.00
3.00
f1 (ppm)

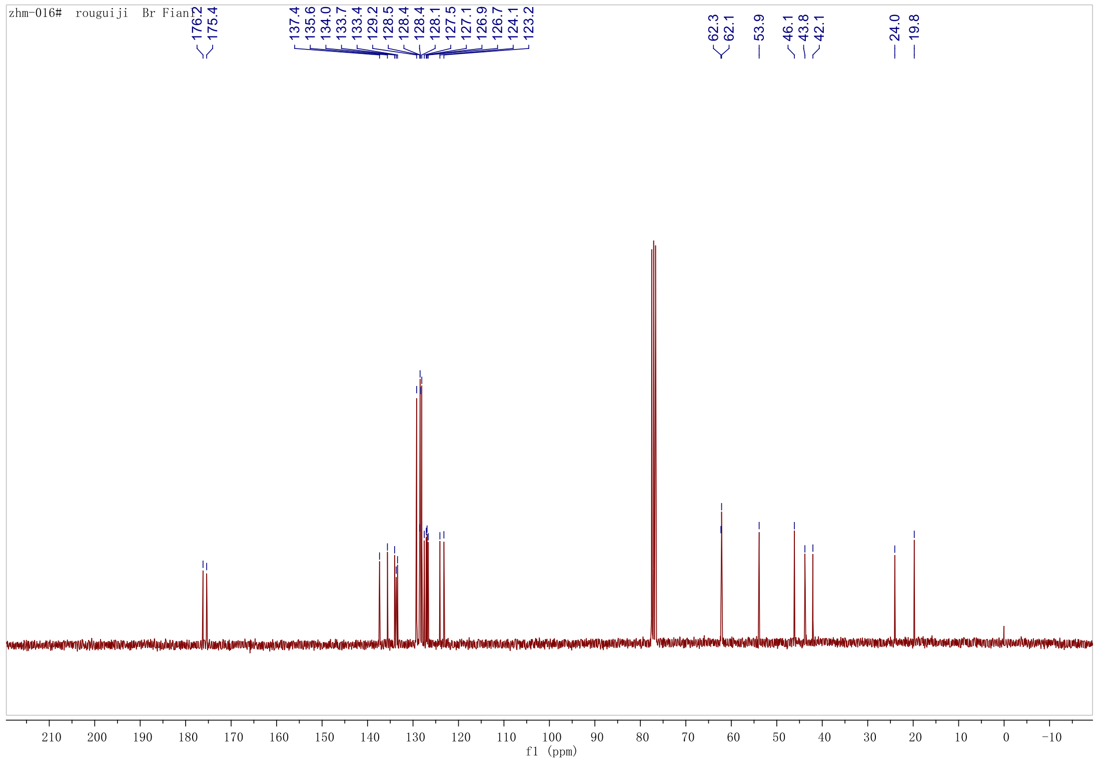
zhm-016# rouguiji Br Fian
176.2
175.4
137.4
135.6
134.0
133.7
133.4
129.2
128.5
128.4
128.4
128.1
127.5
127.1
126.9
126.7
124.1
123.2
62.3
62.1
53.9
46.1
43.8
42.1
24.0
19.8
f1 (ppm)

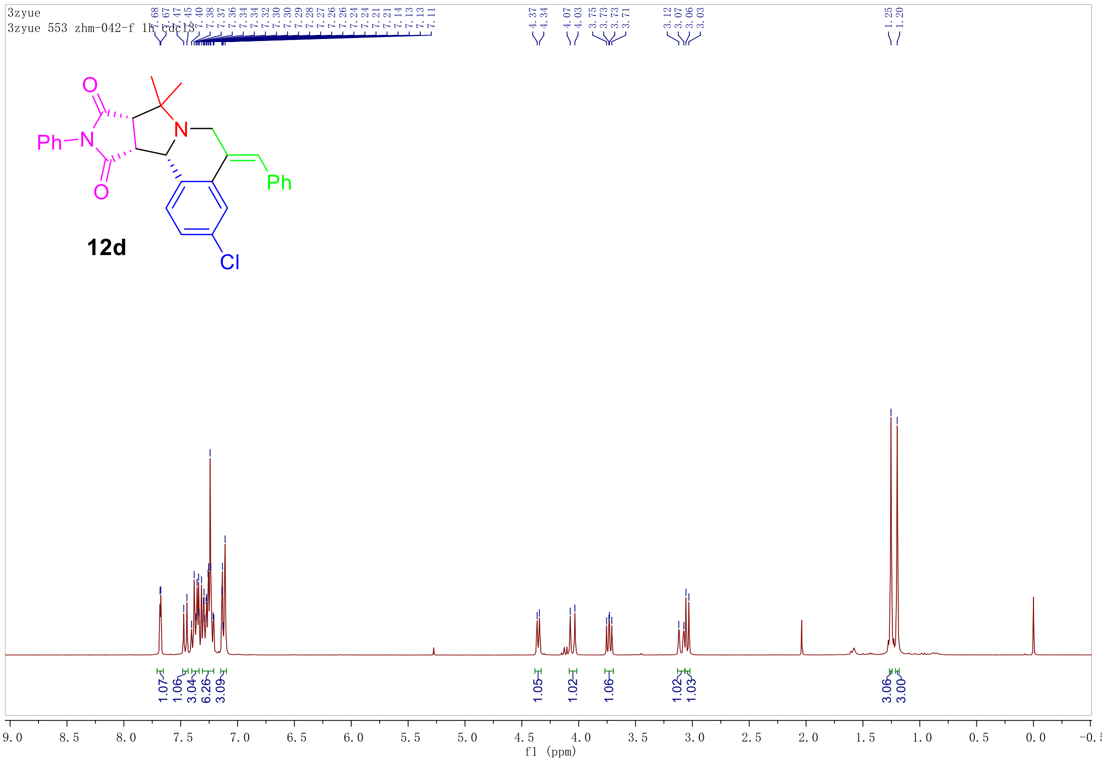
3zyue
3zyue 553 zhm-042-f 1h cdcl3
7.68 7.67 7.47 7.45 7.40 7.38 7.37 7.36 7.34 7.34 7.32 7.30 7.30 7.29 7.28 7.27 7.26 7.26 7.24 7.24 7.24 7.21 7.21 7.14 7.13 7.13 7.11
4.37 4.34 4.07 4.03 3.75 3.73 3.73 3.71 3.12 3.07 3.06 3.03 1.25 1.20
O
O
Ph
N
N
Ph
Cl
12d
1.07 1.06 3.04 6.26 3.09 1.05 1.02 1.06 1.02 1.03 3.06 3.00
9.0 8.5 8.0 7.5 7.0 6.5 6.0 5.5 5.0 4.5 4.0 3.5 3.0 2.5 2.0 1.5 1.0 0.5 0.0
f1 (ppm)

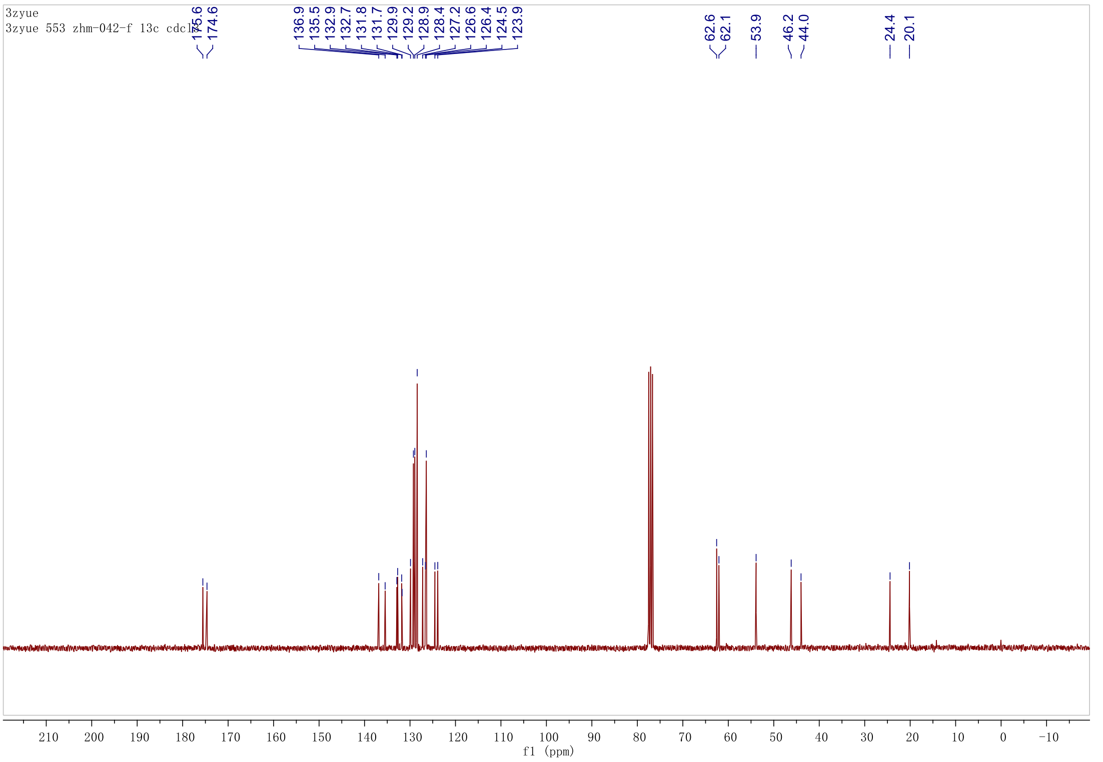
3zyue
3zyue 553 zhm-042-f 13c cdcl3
175.6 174.6
136.9 135.5 132.9 132.7 131.8 131.7 129.9 129.2 128.9 128.4 127.2 126.6 126.4 124.5 123.9
62.6 62.1 53.9 46.2 44.0 24.4 20.1
210 200 190 180 170 160 150 140 130 120 110 100 90 80 70 60 50 40 30 20 10 0 -10
f1 (ppm)
